# Patients With Bicuspid Aortic Stenosis Undergoing Transcatheter Aortic Valve Replacement: A Systematic Review and Meta-Analysis

**DOI:** 10.3389/fcvm.2022.794850

**Published:** 2022-03-16

**Authors:** Yi Zhang, Tian-Yuan Xiong, Yi-Ming Li, Yi-Jun Yao, Jing-Jing He, Hao-Ran Yang, Zhong-Kai Zhu, Fei Chen, Yuanweixiang Ou, Xi Wang, Qi Liu, Xi Li, Yi-Jian Li, Yan-Biao Liao, Fang-Yang Huang, Zhen-Gang Zhao, Qiao Li, Xin Wei, Yong Peng, Sen He, Jia-Fu Wei, Wen-Xia Zhou, Ming-Xia Zheng, Yun Bao, Xuan Zhou, Hong Tang, Wei Meng, Yuan Feng, Mao Chen

**Affiliations:** ^1^Department of Cardiology, West China Hospital, Sichuan University, Chengdu, China; ^2^Department of Radiology, West China Hospital, Sichuan University, Chengdu, China; ^3^Department of Cardiovascular Surgery, West China Hospital, Sichuan University, Chengdu, China

**Keywords:** transcatheter aortic valve replacement (TAVR), meta-analysis, bicuspid aortic valve (BAV), aortic stenosis (AS), systematic review

## Abstract

**Objective:**

We sought to conduct a systematic review and meta-analysis of clinical adverse events in patients undergoing transcatheter aortic valve replacement (TAVR) with bicuspid aortic valve (BAV) vs. tricuspid aortic valve (TAV) anatomy and the efficacy of balloon-expandable (BE) vs. self-expanding (SE) valves in the BAV population. Comparisons aforementioned will be made stratified into early- and new-generation devices. Differences of prosthetic geometry on CT between patients with BAV and TAV were presented. In addition, BAV morphological presentations in included studies were summarized.

**Method:**

Observational studies and a randomized controlled trial of patients with BAV undergoing TAVR were included according to the Preferred Reporting Items for Systematic Reviews and Meta-Analyses (PRISMA) guideline.

**Results:**

A total of 43 studies were included in the final analysis. In patients undergoing TAVR, type 1 BAV was the most common phenotype and type 2 BAV accounted for the least. Significant higher risks of conversion to surgical aortic valve replacement (SAVR), the need of a second valve, a moderate or severe paravalvular leakage (PVL), device failure, acute kidney injury (AKI), and stroke were observed in patients with BAV than in patients with TAV during hospitalization. BAV had a higher risk of new permanent pacemaker implantation (PPI) both at hospitalization and a 30-day follow-up. Risk of 1-year mortality was significantly lower in patients with BAV than that with TAV [odds ratio (OR) = 0.85, 95% CI 0.75–0.97, *p* = 0.01]. BE transcatheter heart valves (THVs) had higher risks of annular rupture but a lower risk of the need of a second valve and a new PPI than SE THVs. Moreover, BE THV was less expanded and more elliptical in BAV than in TAV. In general, the rates of clinical adverse events were lower in new-generation THVs than in early-generation THVs in both BAV and TAV.

**Conclusions:**

Despite higher risks of conversion to SAVR, the need of a second valve, moderate or severe PVL, device failure, AKI, stroke, and new PPI, TAVR seems to be a viable option for selected patients with severe bicuspid aortic stenosis (AS), which demonstrated a potential benefit of 1-year survival, especially among lower surgical risk population using new-generation devices. Larger randomized studies are needed to guide patient selection and verified the durable performance of THVs in the BAV population.

## Introduction

Transcatheter aortic valve replacement (TAVR) is now a well-established treatment option for patients with symptomatic severe aortic stenosis (AS) in all spectrums of surgical risk (1). According to surgical experience, bicuspid aortic valve (BAV) anatomy may comprise up to 50% of low-risk patients (2). Therefore, when expanded to patients of lower risks and younger age, TAVR procedures are anticipated to treat more patients with BAV. However, all pivotal randomized controlled trials comparing TAVR with surgical aortic valve replacement (SAVR) excluded patients with BAV due to a higher risk of procedural complications, such as paravalvular leakage (PVL), stroke, new permanent pacemaker implantation (PPI), and annular rupture (3). Anatomical features such as the nontubular shape from the annulus to the leaflet tips and heavier calcification in patients with BAV often result in more common malposition of transcatheter heart valves (THVs) than patients with tricuspid aortic valve (TAV), as well as in conduction disturbances or PVL (4, 5). Previous meta-analyses of cohort studies have reported that, compared to patients with TAV, patients with BAV were at a higher risk of procedural complications, such as the conversion to SAVR, the implantation of a second valve, a moderate or severe PVL, and the device failure (6). In addition, new-generation devices were reported to have a lower risk of adverse events compared to early-generation devices in BAV, while balloon-expandable (BE) valves were associated with the lower need of a second valve and a new PPI than self-expanding (SE) valves (6).

With the accumulation of experience and an iteration of prosthesis, TAVR is now used more frequently for patients with BAV (7–10), enabling detailed comparisons to be updated. Because of the lack of the corresponding guideline and normative practical guidance for TAVR in the BAV population, pressing the need for a reliable assessment on the efficacy and safety of TAVR procedures in patients with BAV existed. Therefore, we systematically reviewed related researches and hereby summarized the BAV morphological presentations, clinical adverse events of TAVR in patients with BAV vs. TAV, as well as the efficacy of BE vs. SE valves in patients with BAV. Comparisons of early- vs. new-generation devices were performed where available. Moreover, the geometry of THV on CT after TAVR was compared between patients with BAV and TAV.

## Method

### Search Strategy, Selection Criteria, and Data Extraction

The composition of this current review was in line with an evidence-based set of items in the Preferred Reporting Items for Systematic Reviews and Meta-Analyses (PRISMA) (11). Associated checklist is presented in Supplementary Table S9. The search of original articles was conducted by two independent investigators, YZ and TYX, on Medline, Embase, Cochrane Central Register of Controlled Trials (CENTRAL), conference proceedings for the Scientific Sessions of the American College of Cardiology, American Heart Association, European Society of Cardiology, Transcatheter Cardiovascular Therapeutics, EuroPCR, and Transcatheter Valve Therapeutics. Search code included TAVI OR TAVR OR “percutaneous aortic valve” OR “transcatheter aortic valve”) AND (bicuspid OR BAV) on Medline, Embase and conference proceedings; #1 TAVI, #2 TAVR, #3 percutaneous aortic valve, #4 transcatheter aortic valve, #5 bicuspid, #6 BAV, #7 (#1 OR #2 OR #3 OR #4) AND (#5 OR #6) on CENTRAL. The search was last updated on September 22, 2021. Exclusion criteria were: (1) duplicate publication; (2) articles without primary data; and (3) non-English research. Inclusion criteria were one of the followings: (1) a comparison of clinical adverse events of TAVR between BAV and TAV, or a comparison of BE and SE valve outcomes in patients with BAV; (2) a comparison of THV geometry on CT after TAVR between BAV and TAV; both with the availability of binary primary outcome data. The assessment of article quality and extraction of relevant data were done by YZ and YML independently. Data extracted from the included studies and used for all analyses in the review are presented in Supplementary Material.

The aim of this study was set to answer: (1) the proportion of different phenotypes of BAV in the included studies; (2) a comparison of clinical outcomes and procedural complications after TAVR in patients with BAV vs. TAV, including a subgroup analysis stratified by early- and new-generation devices; (3) a comparison of clinical outcomes and procedural complications in patients with BAV after TAVR between BE and SE valves, including a subgroup analysis stratified into early- and new-generation devices; and (4) differences of BE and SE THV geometry on CT after TAVR in patients with BAV.

Early-generation TAVR devices included Sapien (Edwards Lifesciences), Sapien XT (Edwards Lifesciences), CoreValve (Medtronic), and Venus A-Valve (Venus MedTech Inc.). New-generation devices included Sapien 3 (Edwards Lifesciences), Lotus (Boston Scientifics), Evolut R and Pro (Medtronic), Acurate Neo (Boston Scientific), and Portico (Abbott). BE devices included Sapien, Sapien XT, and Sapien 3 valves (Edwards Lifesciences); SE devices included CoreValve, Evolut R and Pro (Medtronic), Accurate Neo (Boston Scientifics), Portico (Abbott), Venus A-Valve (Venus MedTech), and Lotus (Boston Scientifics). The year of publication, study design, the number of enrolled centers, countries, the mean or median age of population, the mean or median score of surgical risks, and the number of enrolled patients were collected from each study. Overlapping population of the included articles was screened. The publication of a smaller sample size in studies with overlapping population was then excluded from the subsequent meta-analysis. Discrepancies in the selection of relevant studies and data extraction were solved by a discussion with a third evaluator (YML).

### Outcomes of Interest

Bicuspid aortic valve was subclassified as type 0, type 1 (grouped by left–right coronary cusp fusion, left noncoronary cusp fusion, and right noncoronary cusp fusion), and type 2 according to Sievers' classification (12). The proportions of each subtype were compared among regions grouped into the USA, Europe, China, and multiregional areas (data from multicenter studies including Europe, North America, and other Asia-Pacific regions).

Transcatheter aortic valve replacement-specific outcomes were defined according to the Valve Academic Research Consortium 3 (VARC-3), while study-specific definitions remained as they were based on the corresponding articles (13). Adverse events of interest at hospitalization included the conversion to SAVR, coronary obstruction, the need of a second valve, device failure (procedural mortality, the incorrect positioning of a single prosthetic heart valve into the proper anatomical location, prosthesis-patient mismatch, mean aortic valve gradient > 20 mmHg, peak velocity > 3 m/s, or moderate/severe prosthetic valve regurgitation), annular rupture, new-onset atrial fibrillation (NO-AF), life-threatening or major bleeding, major vascular complications, acute kidney injury (AKI), myocardial infarction (MI), a moderate or severe PVL, stroke, a new PPI, MI, and mortality; adverse events of interest at a 30-day follow-up included life-threatening or major bleeding, major vascular complications, AKI, and MI; and adverse events of interest at a 1-year follow-up included a moderate or severe PVL, stroke, a new PPI, MI, and mortality.

Transcatheter heart valve geometry and position were demonstrated by: (1) THV expansion, i.e., (the observed THV external area/device labeled size) × 100% at inflow, annulus, and the outflow of the valve frame; (2) THV eccentricity index = [1–(minimum external THV diameter/maximum external THV diameter)] × 100%; and (3) THV implantation depth, i.e., the distance from the inflow of the prosthesis to the floor of right, left, and non-coronary cusps.

### Statistical Analysis

The results of meta-analysis were summarized as odds ratios (ORs) or mean difference (MD) and 95% CIs. Heterogeneity across studies was tested by the Cochran's *Q* statistic and Higgins' and Thompson's *I*^2^ statistics (14). The Freeman–Tukey Double Arcsine method were used for each pooled event rate (%) according to valve generations and aortic valve morphologies. *I*^2^ > 50% and *p* ≤ 0.1 was considered to be a significant heterogeneity, where random-effect models were used. Otherwise, fixed-effect model was used for an analysis. *p* < 0.05 was considered as statistically significant for other results. All analyses were conducted using Review Manager version 5.3 (available from http://tech.cochrane.org/revman).

### Quality Assessment

All included studies [except one (15)] were non-randomized studies, so study qualities were evaluated by the ROBINS-I tool (16). Publication bias was presented in funnel plots. The conduction and composition of this review were conformed to the PRISMA 2020 guideline (17).

## Results

**Figure 1:**
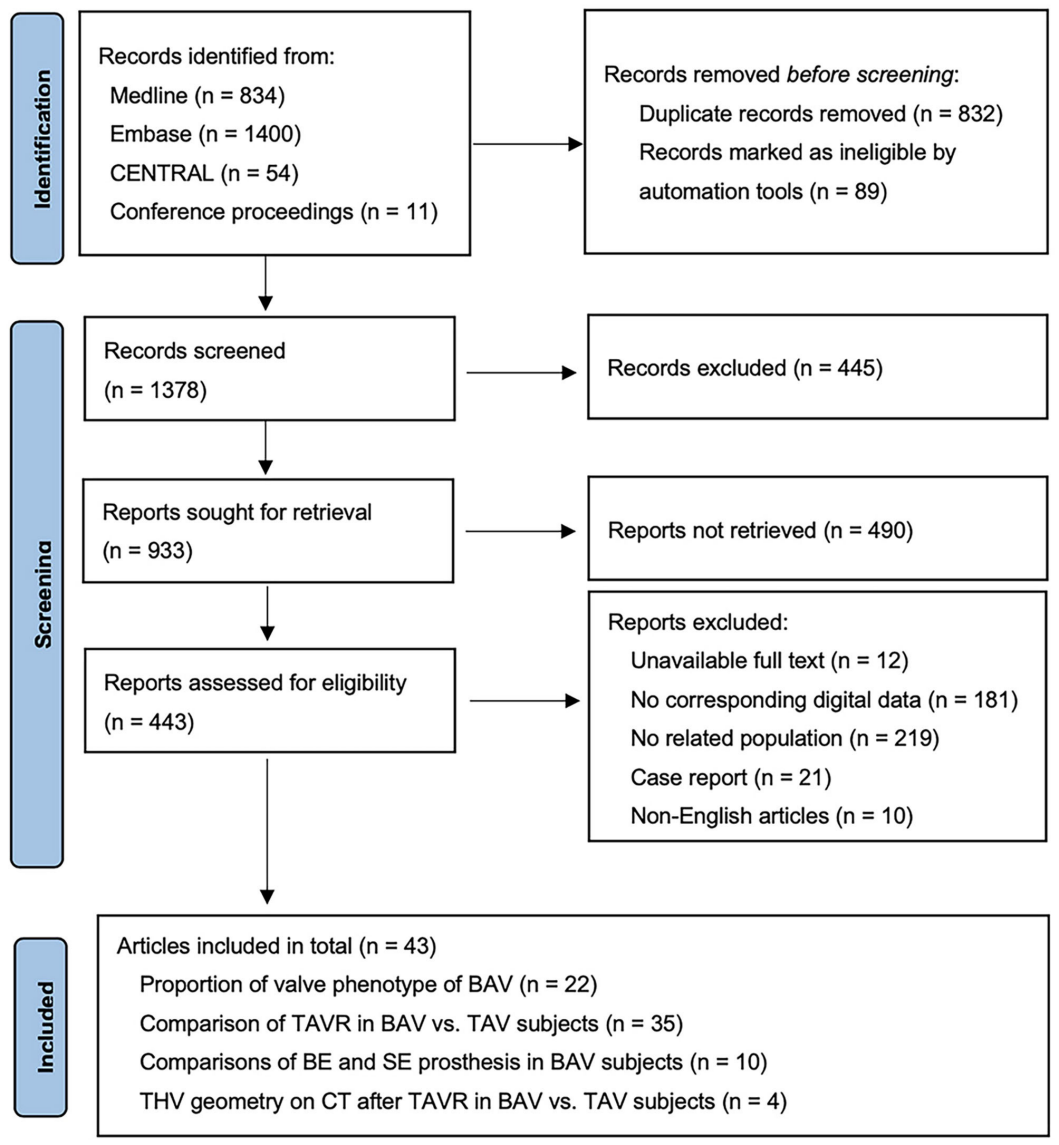
Preferred Reporting Items for Systematic Reviews and Meta-Analyses (PRISMA) 2020 flow diagram.

The study flow is presented as the PRISMA 2020 flow diagram (Figure 1). A total of 22 studies (2,546 patients with BAV) were included for the analysis of BAV phenotypes (8, 10, 18–37). A total of 35 studies (including 139,058 patients: 15,700 BAV and 123,358 TAV) were analyzed for comparisons between BAV and TAV (7–10, 15, 18–30, 38–54), while 10 studies (including 1,294 patients: 805 BE and 489 SE) were analyzed for the difference of BE vs. SE in patients with BAV after TAVR (32–38, 40, 55). In addition, four studies (including 551 patients: 149 patients with BAV and 402 patients with TAV) were analyzed for the difference of THV geometry between BAV and TAV after TAVR (21, 31, 41, 53).

### Proportion of the Different Types of BAV in the Included Studies

**Figure 2:**
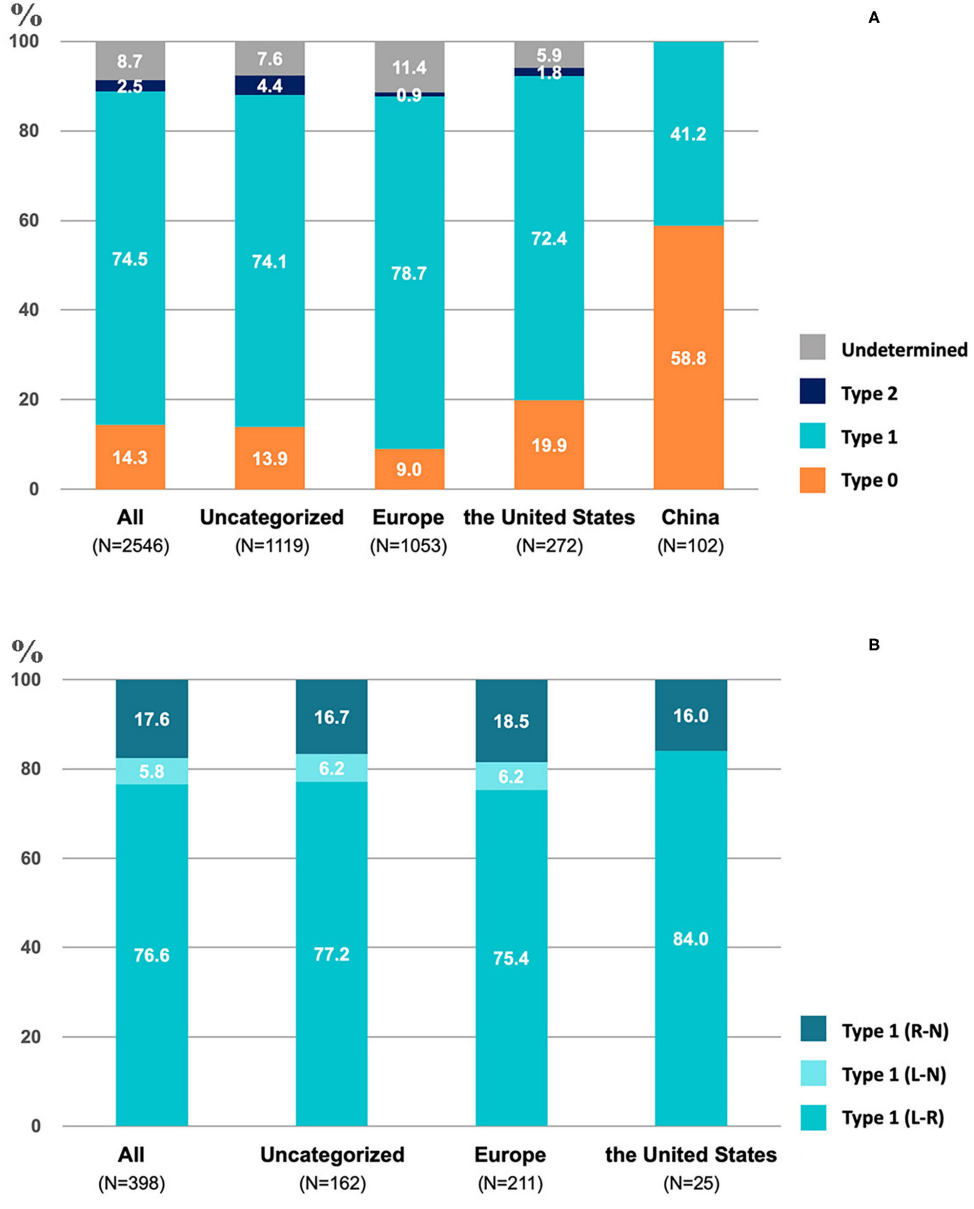
The proportion of different valve phenotypes of bicuspid aortic valve (BAV) **(A)** and type-1 **(B)** in included studies.

Type 1 BAV accounted for 74.5% (1,897/2,546) of patients, being the most frequently encountered BAV subtype (Figure 2A). The predominance of type 1 BAV was presented in Europe, the USA, and multiregional studies, accounting for 78.7% (829/1,053), 72.4% (197/272), and 74.1% (829/1,119) of patients, respectively. However, Chinese patient population demonstrated a different distribution, with 58.8% (60/102) of type 0 and 41.2% (42/102) of type 1 BAV. In addition, type 2 BAV was least commonly seen in all studies with a proportion of 2.5% (64/2,546) in total, 4.4% (49/1,119), 0.9% (9/1,053), 1.8% (5/272), and 0, respectively, in multiregional studies, Europe, the USA, and China. A total of 398 patients with type 1 BAV were included for further analysis of fusion patterns (Figure 2B). The L-R coronary cusp fusion was the most common pattern with a proportion of 76.6% (305/398), and the L-N coronary cusp fusion was the least common pattern with a proportion of 5.8% (23/398). Similar distributions of the L-R and L-N fusion was presented in type 1 BAV from Europe, the USA, and multiregional studies.

### Comparisons Between BAV and TAV

**Figure 3:**
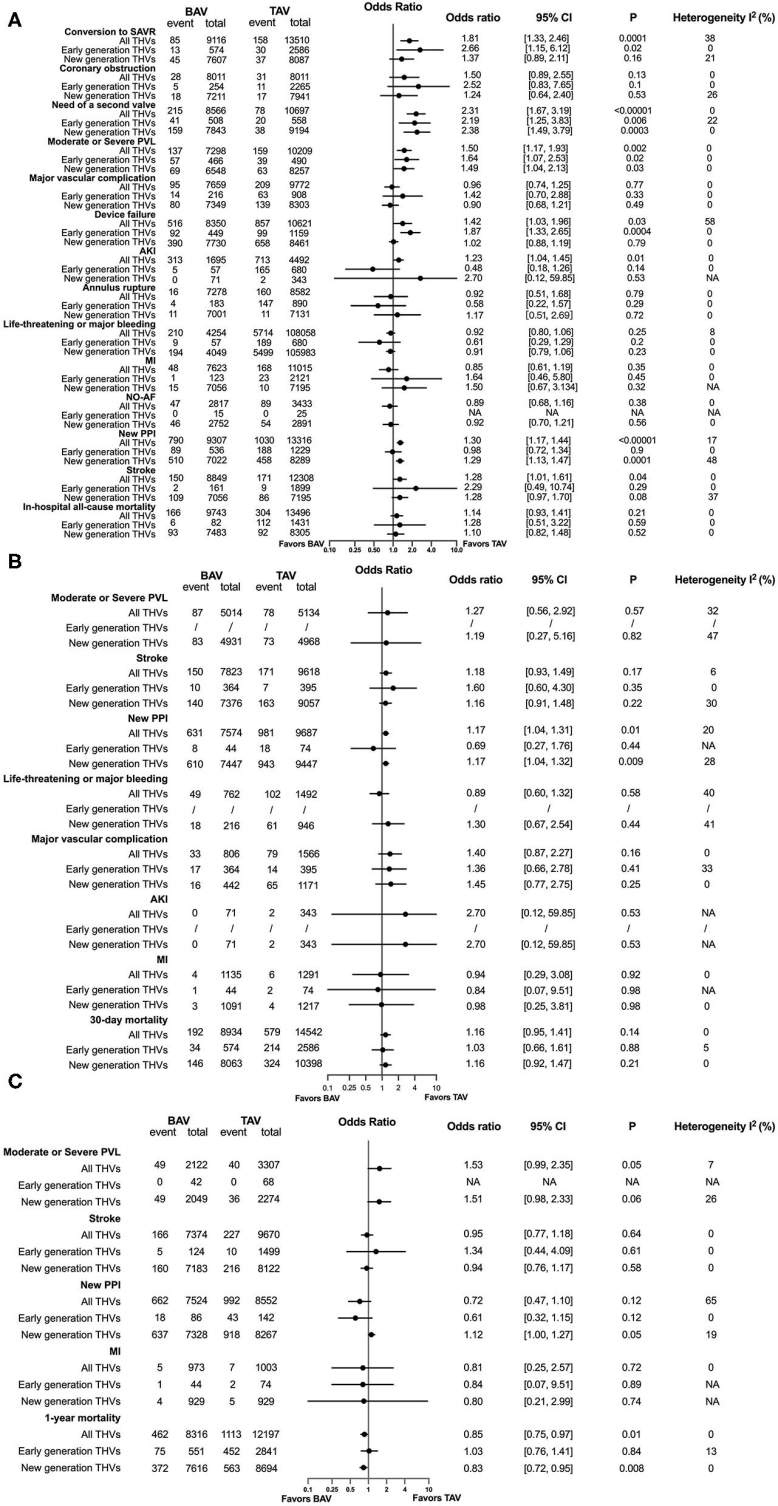
Procedural complications and outcomes between BAV and tricuspid aortic valve (TAV) at in-hospital time **(A)**, in a 30-day **(B)**, and in a 1-year **(C)** follow-up. SAVR, surgical aortic valve replacement; PVL, paravalvular leakage; AKI, acute kidney injury; MI, myocardial infarction; NO-AF, new-onset atrial fibrillation; PPI, permanent pacemaker implantation.

Baseline of patients and the characteristics of the included studies are summarized in Supplementary Table S1. In-hospital, 30-day and 1-year procedural complications and outcomes are presented in Figures 3A–C, respectively. All original records of meta-analysis are presented in Supplementary Figure S1. In terms of in-hospital analysis, patients with BAV treated by TAVR were at a higher risk of the need of a second valve (OR = 2.31, 95% CI 1.67–3.19, *p* < 0.00001) and a moderate or severe PVL (OR = 1.50, 95% CI 1.17–1.93, *p* = 0.002) than patients with TAV, with consistent results stratified by early- and new-generation devices. Moreover, patients with BAV were at an increased risk of the conversion to SAVR (OR = 1.81, 95% CI 1.33–2.46, *p* = 0.0001) and device failure (OR = 1.42, 95% CI 1.03–1.96, *p* = 0.03), with a consistent result in patients receiving early-generation devices. A new PPI (OR = 1.30, 95% CI 1.17–1.44, *p* < 0.00001) was more common in patients with BAV than patients with TAV, as well as in new-generation devices receivers. Patients with BAV were at a higher risk of AKI (OR = 1.23, 95% CI 1.04–1.45, *p* = 0.01) and stroke (OR = 1.28, 95% CI 1.01–1.61, *p* = 0.04) than patients with TAV, but no significant differences were observed when stratified into early and new-generation devices. At 30-day post TAVR, the new PPI (OR = 1.17, 95% CI 1.04–1.31, *p* = 0.01) tended to be more common in BAV than in TAV, with the results in accordance with new-generation devices (OR = 1.17, 95% CI 1.04–1.32, *p* = 0.009). In addition, no differences were observed in 30-day mortality (OR = 1.16, 95% CI 0.95–1.41, *p* = 0.14). At a 1-year follow-up, patients with BAV demonstrated a lower mortality rate than patients with TAV (OR = 0.85, 95% CI 0.75–0.97, *p* = 0.01), with consistent results presented in patients using early-generation devices (OR = 0.83, 95% CI 0.72–0.95, *p* = 0.008).

**Figure 4:**
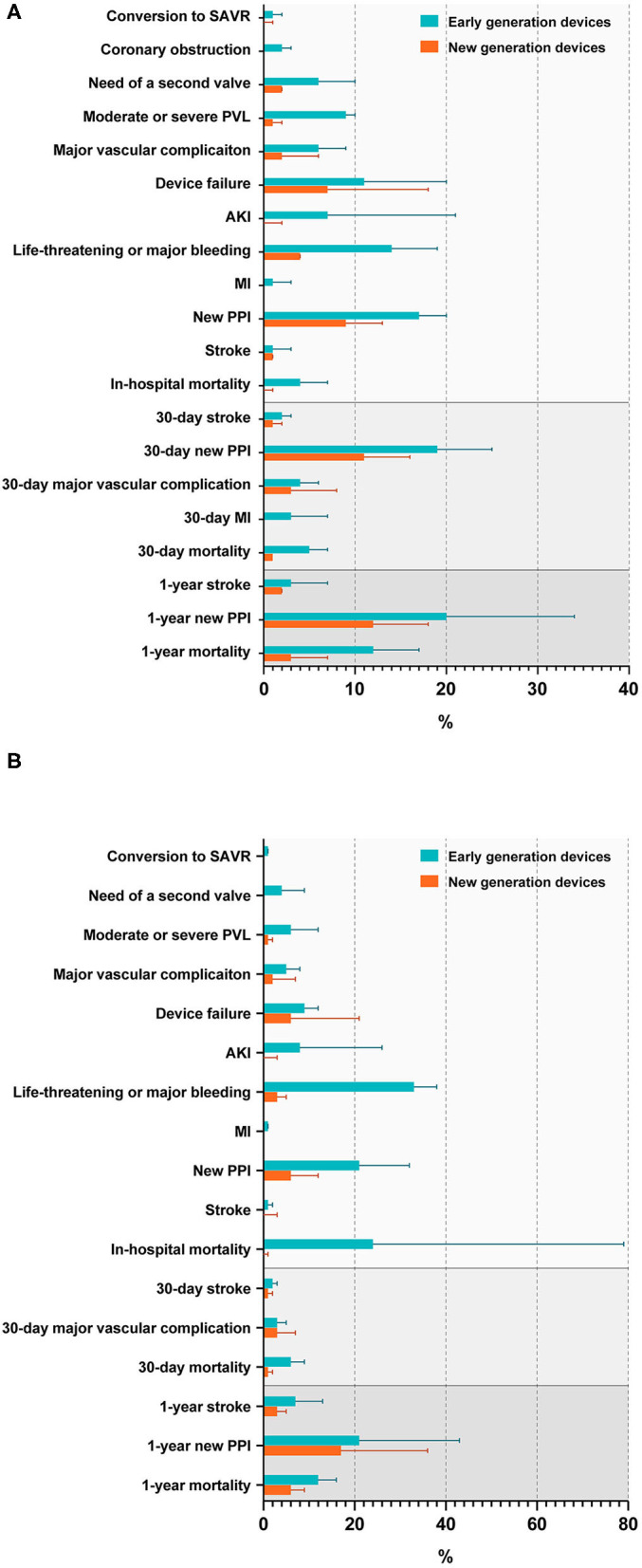
Rates of procedural complications and outcomes in patients with BAV **(A)** and TAV **(B)**. SAVR, surgical aortic valve replacement; PVL, paravalvular leakage; AKI, acute kidney injury; MI, myocardial infarction; PPI, permanent pacemaker implantation.

Rates of complications and adverse outcomes were generally higher in population using early-generation devices than using new-generation devices, including the conversion to SAVR, the need for a second valve, a moderate or severe PVL, major vascular complications, the device failure, AKI, life-threatening or major bleeding, MI, a new PPI, stroke, and mortality in hospital; stroke, major vascular complications, mortality at a 30-day follow-up; stroke, new PPI, mortality at a 1-year follow-up in BAV and TAV subjects, in addition with an in-hospital coronary obstruction, a new 30-day PPI, a 30-day MI in the BAV population (Figures 4A,B, Supplementary Tables S7, S8). A significant heterogeneity existed in the analysis of in-hospital device failure in all THVs (*I*^2^ = 58%, *p* = 0.003) and a 1-year new PPI in all THVs (*I*^2^ = 65%, *p* = 0.006) between patients with BAV and TAV. The risk of bias of the included studies is summarized in Supplementary Table S2, and publication bias is presented as a funnel plot in Supplementary Figure S3.

### Comparisons Between BE and SE Valves in Patients With BAV

**Figure 5:**
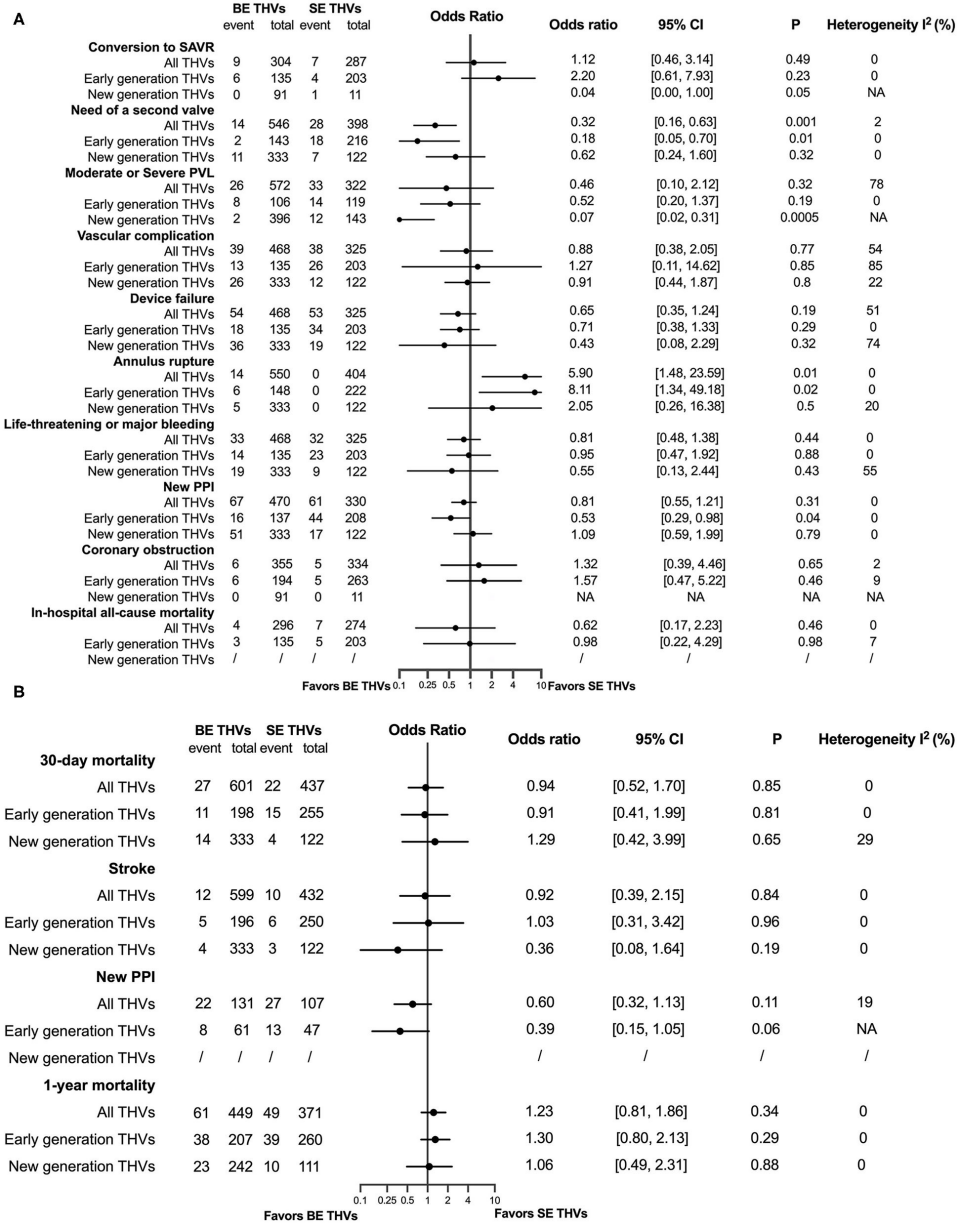
A comparison between balloon-expandable (BE) and self-expanding (SE) valves in patients with BAV at in-hospital time **(A)**, and in a 30-day and a 1-year **(B)** follow-up. SAVR, surgical aortic valve replacement; PVL, paravalvular leakage; PPI, permanent pacemaker implantation.

The characteristics of the included studies and baseline of patients in the subanalysis of the efficacy of BE vs. SE in patients with BAV are presented in Supplementary Table S3. The in-hospital and follow-up results are presented in Figures 5A,B, respectively. Patients with BAV using BE THVs were at a lower risk of the need of a second valve (OR = 0.35, 95% CI 0.17–0.70, *p* = 0.003) than SE THVs, and the consistent trend was also observed in early-generation devices (OR = 0.18, 95% CI 0.05–0.70, *p* = 0.01). A new PPI tended to be less common only in the early generation of BE THVs than SE THVs (OR = 0.53, 95% CI 0.29–0.98, *p* = 0.04), while a moderate or severe PVL was less common in only new-generation BE THVs than SE THVs (OR = 0.07, 95% CI 0.02–0.31, *p* = 0.0005). However, patients with BAV were at a higher risk of annular rupture in BE THVs than in SE THVs (OR = 4.84, 95% CI 1.39–16.85, *p* = 0.01), similarly in early-generation devices (OR = 8.11, 95% CI 1.34–49.18, *p* = 0.02). In addition, the 30-day (OR = 0.96, 95% CI 0.53–1.76, *p* = 0.9) and 1-year mortality (OR = 1.11, 95% CI 0.73–1.71, *p* = 0.62) between BE and SE THVs were not different. All original records of the meta-analysis are presented in Supplementary Figure S2. The pooled results of meta-analyses of in-hospital moderate or severe PVL in all THVs (*I*^2^ = 78%, *p* = 0.001), vascular complications in all THVs and first-generation THVs (*I*^2^ = 54%, *p* = 0.11; *I*^2^ = 85%, *p* = 0.009), device failure in all THVs and new-generation THVs (*I*^2^ = 51%, *p* = 0.13; *I*^2^ = 74%, *p* = 0.05), and a life-threatening or major bleeding one in new-generation THVs (*I*^2^ = 55%, *p* = 0.13) between BE and SE THVs in patients with BAV had a significant heterogeneity. The risk of bias of the included studies is summarized in Supplementary Table S4, and publication bias is presented as a funnel plot in Supplementary Figure S4.

### THV Geometry After TAVR in Patients With BAV vs. TAV

**Figure 6:**
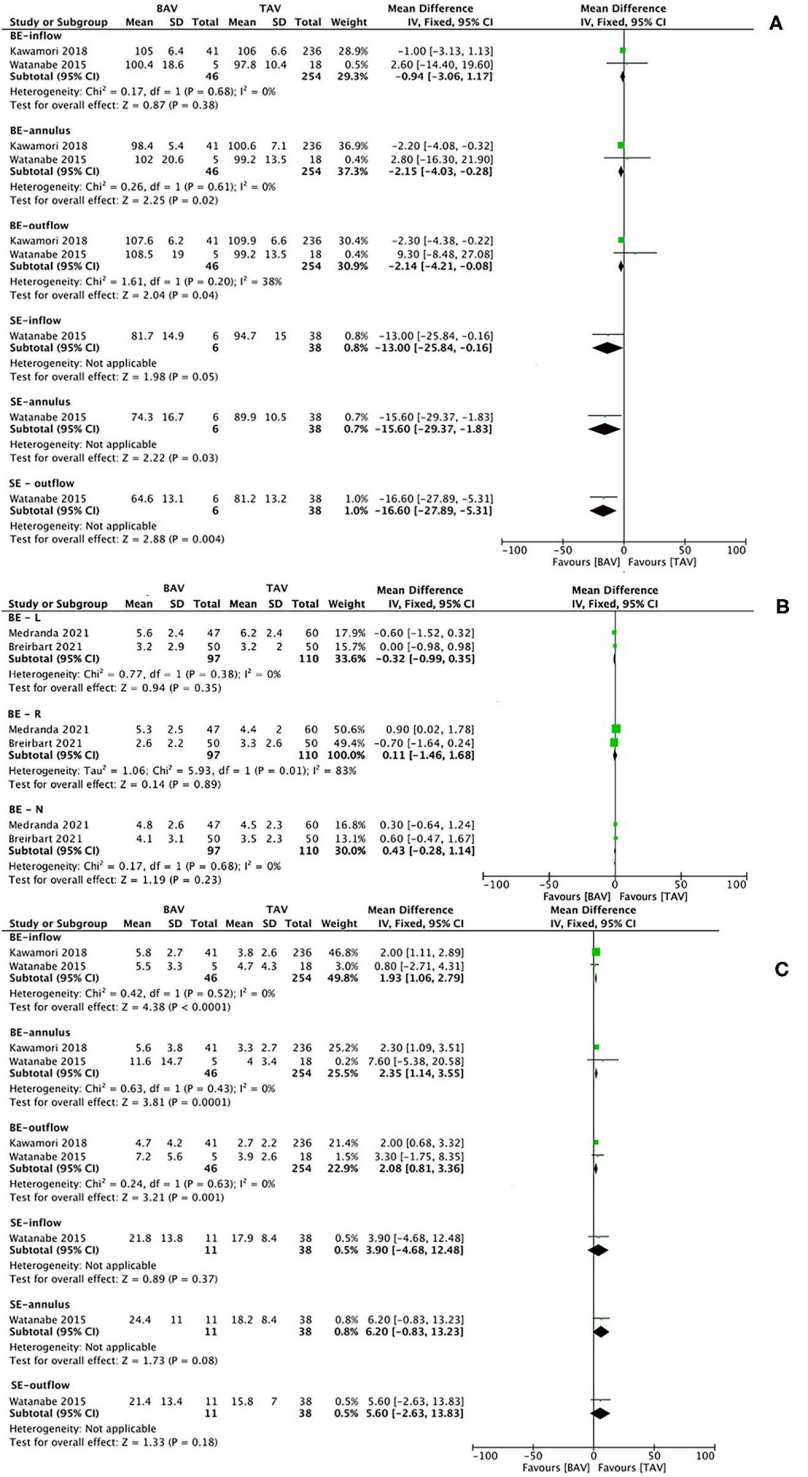
Transcatheter heart valve (THV) expansion **(A)**, implantation depth **(B)**, and eccentricity index **(C)** on CT at different levels after TAVI in patients with BAV vs. TAV. CT image analysis of THVs, dividing into balloon-expandable and SE valves, in terms of the expansion at the inflow, annulus, and outflow level **(A)**; implantation depth below left, right and none coronary sinus **(B)**, and the eccentricity index at the inflow, annulus, and outflow level **(C)**. BE, balloon-expandable; SE, self-expanding; BE-L, balloon-expandable valve—left coronary sinus; BE-R, balloon-expandable valve—right coronary sinus; BE-N, balloon-expandable valve—non-coronary sinus.

The characteristics of studies and baseline of patients for the subanalysis of THV geometry are summarized in Supplementary Table S5, and the results of meta-analysis are presented in Figure 6. The mean BE THV expansion after TAVR at the annulus (MD −2.15, 95% CI −4.03 to −0.28, *p* = 0.02) and outflow level (MD −2.14, 95% CI −4.21 to −0.08, *p* = 0.04) was significantly smaller in patients with BAV than in patients with TAV. According to one original article (41), the mean SE THV expansion of the BAV population on CT at the inflow (MD −13.00, 95% CI −25.84 to −0.16, *p* = 0.05), annulus (MD −15.60, 95% CI −29.37 to −1.83, *p* = 0.03), and outflow level (MD −16.60, 95% CI −27.89 to −5.31, *p* = 0.004) was smaller than that of the TAV population. Moreover, BE THV eccentricity index was larger in patients with BAV than in patients with TAV at the inflow (MD 1.93, 95% CI 1.06–2.79, *p* < 0.0001), annulus (MD 2.35, 95% CI 1.14–3.55, *p* = 0.0001), and outflow level (MD 2.08, 95% CI 0.81–3.36, *p* = 0.01). No significant differences were witnessed in SE THV. In addition, BE THV implantation depth was not different between the two groups. No significant heterogeneity was observed in the pooled analysis. The risk of bias of the included studies is summarized in Supplementary Table S6, and the publication bias is presented as a funnel plot in Supplementary Figure S5.

## Discussion

This meta-analysis represents the up-to-date pooling of most extensive evidence of TAVR in patients with BAV. The major findings are: (1) type 1 BAV accounted for the largest proportion of BAV subtypes in multiregional studies and studies in Europe and the USA, while type 0 was more prevalent than type 1 in China. type 2 BAV was the least common finding in all regions. In terms of type 1 morphology, L-R coronary cusp fusion was the most common pattern while L-N coronary cusp fusion was the least common pattern. (2) Patients with BAV were at a higher risk of the conversion to SAVR, the need of a second valve, a moderate or severe PVL, the device failure, AKI, a new PPI, and stroke during hospitalization than TAV. A new PPI remained more common among patients with BAV than among patients with TAV at a 30-day follow-up. Both in-hospital and 30-day mortality between the two groups were not different, but 1-year mortality was lower in patients with BAV than in patients with TAV. (3) BE THVs were at a higher risk of annular rupture but the lower need for a second valve than SE THVs for patients with BAV. In addition, the incidence of a new PPI was higher in BE THVs than in SE THVs only in case of early-generation valves. (4) In terms of BE THV, it was less expanded at the annular and outflow level in BAV than in TAV, while more elliptical in BAV than in TAV at the inflow, annular, and outflow level. The implantation depth of BE THV was similar in the two morphologies. (5) Adverse events were less in new-generation devices than in early-generation devices in general, for patients with both BAV and TAV.

Bicuspid aortic valve is the most common isolated cause of AS among patients aged 50–70 years (56). Now that a series of randomized controlled trials demonstrate noninferior or superior outcomes of TAVR vs. SAVR irrespective of risk profiles, TAVR is expected to expand its utilization and more and more younger patients with bicuspid AS would become the candidates for TAVR. In addition, the latest guideline for valvular heart disease has recommended TAVR as an alternative to SAVR in patients with symptomatic BAV having severe AS despite no solid evidence (1). However, patients with BAV remain challenging for TAVR given its complex anatomical features such as heavy calcification with or without raphe and a concomitant dilatation of the ascending aorta, thus are still at a high risk of device malposition, underexpansion, and other procedural complications even using new-generation devices (19, 34). Thus, in this meta-analysis, we updated current evidence in TAVR for BAV while exploring regional differences in BAV subtypes, device performance, and THV geometry.

According to the number of cusps and presence of raphes, Sievers et al. have classified BAV into different phenotypes (12). The proportion of type 0 BAV seems to be higher in China than in western countries, which was confirmed by our pooled analysis. Although a previous study on Asian patients has shown a prevalence of type 1 BAV, the differences in imaging modality (i.e., MSCT vs. echocardiography), targeting patient population (i.e., AS being evaluated for TAVR vs. BAV being diagnosed with echocardiography), and the enrollment without Chinese centers might explain the divergence from our result (57–59). Type 0 morphology can pose additional challenges to TAVR. Difficulties exist in determining the virtual annulus with only two hinge points (60). A lower rate of VARC-2 defined device success (72% vs. 86.7%; *p* = 0.07) and a higher rate of mean trans-prosthetic gradient ≥ 20 mmHg (24% vs. 6%, *p* = 0.007) was reported in type 0 BAV than in type 1 (57). Such regional disparities might be a hint for underlying ethnic issues in the development of BAV, while also suggesting the need to consider BAV subtypes when interpreting TAVR results from different countries.

The in-hospital and 30-day mortality between patients with BAV and TAV receiving TAVR were not different, but patients with TAV (*n* = 12,197) seemed to have 1-year mortality higher than patients with BAV (*n* = 8,316), as well as in patients with TAV (*n* = 8,694) and BAV (*n* = 7,616) who received new-generation devices. The significance of survival risk differences in all THV receivers was presented when verified by fixed- (as presented in our results) and random-effect models (OR = 0.86, 95% CI = 0.76–0.98, *p* = 0.02; *I*^2^ = 0%, *p* = 0.80), which indicated the validity of the result. Most patients included in this analysis were from a latest propensity score matched research (including 6,995 BAV and 6,995 TAV; weighted 74.5% in overall meta-analysis), which analyzed consecutive patients undergoing TAVR with third-generation SAPIEN 3 and fourth-generation SAPIEN 3 Ultra valve in the STS/TVT Registry from June 2015 to October 2020, with a relatively low STS-PROM (4.0 ± 3.7 in BAV and 4.0 ± 3.5 in TAV) (54). Although the result in the original research did not show significant differences in 1-year survival (HR = 0.90, 95% CI = 0.78–1.04), the 1-year mortality of BAV (8.6%, 357/6,995) was numerically lower than that of TAV (9.8%, 417/6,995). Consequently, patients with BAV showed better 1-year survival than patients with TAV in the pooled results, indicating the potential survival benefit of the latest BE THVs applied in relatively low-risk patients with TAVR.

Although the rates of procedural complications decreased significantly with the improvement of devices, patients with BAV were still at a higher risk of the conversion to SAVR, the need of a second valve, a moderate or severe PVL, the device failure, AKI, stroke, and a new PPI. Anatomical features (i.e., longer leaflets, more severe valve calcification, and unequal-sized leaflets) and practical challenges (i.e., difficulty in valve sizing and determining the virtual annulus with only two hinge points) in BAV As subjects might bring the THV eccentricity and an incomplete prosthesis expansion during the procedure, as shown in our results, resulting in THV malposition or even aortic root injury (61). Therefore, there were higher risks of the implantation of two valves, PVL and urgent conversion to SAVR, consequently leading to a higher device failure. More AKIs in patients with BAV might be related to the volume of contrast used and the longer procedural time (7). A higher risk of stroke in patients with BAV was demonstrated during hospitalization but not at a 30-day and 1-year follow-up. This might be related to a heavier calcium burden in BAV and more usage of balloon pre-dilation during the procedure. Therefore, the cautious usage of balloon pre-dilation and limitation of the dilation times might be considered during the TAVR procedure in BAV subjects to achieve lower risk of stroke. In addition, a new PPI in hospital and in a 30-day follow-up were more common in patients with BAV than in patients with TAV, particularly in subjects receiving new-generation THVs, which might be caused by the compression on the conduction system beneath the membranes part of interventricular septum by the inflow stent of THVs, leading to conduction disturbances. Newly developed retrievable new-generation devices seemed to be invalid in lowering the risk of a new PPI in patients with BAV even with a potential advantage of implanting in the target landing zone. However, clinical adverse events were comprehensively reduced when devices were iterated into new generations, in both BAV and TAV population, indicating the importance of an improvement in the device design.

The need of a second valve were higher in self-expanding valves than in BE valves. The anchoring of BE THVs is achieved by actively pushing away native structures through balloon dilatation, which is easier to be implanted in the target landing zone. However, the SE THVs are more likely to be malpositioned because of the passive adaptation of native valve structures. New generations of SE valves have largely overcome malposition by the ability of recapturing and repositioning. Additionally, BE THVs demonstrated a higher risk of annular rupture than SE THVs, which indicated the preference of SE THVs in patients with BAV with risk factors for annular rupture such as asymmetric calcification. Moreover, less aggressive inflating of balloons should be taken into consideration in these patients if BE THV is used. A new PPI was more common in the early generation of SE THVs than BE THVs in BAV but not in new generations of devices, which was related to an inherent difference of the designation of SE and BE THVs. However, the risk of a moderate and severe PVL still seemed to be higher in SE than in BE THVs in BAV even with new-generation devices in one study (36). Valve sizing (i.e., discretion of supra-annular sizing vs. annular sizing) for patients with BAV undergoing TAVR is important, which is frequently encountered in clinical practice. Further analysis in this aspect was not conducted because of limited original articles. There was one published meta-analysis elucidating the outcomes of supra-annular sizing for TAVR in the BAV population (62).

Our result updated new findings of higher risks of AKI and a 30-day new PPI in patients with BAV than in patients with TAV undergoing TAVR when compared with previous meta-analyses. Moreover, 1-year mortality was firstly demonstrated to be significantly higher in TAV than in BAV TAVR receivers. We also identified a higher risk of in-hospital new PPI in patients with early-generation SE THVs than BE THVs in patients with BAV. In addition, the pooled results for the proportion of BAV subtypes being treated by TAVR in different regions and the THV geometry on CT in patients with BAV vs. TAV were displayed, which were not covered previously. Although the use of TAVR in BAV is promising, to further expand indications for TAVR in bicuspid AS, large randomized trials comparing TAVR and SAVR in this population are needed, especially for low-risk patients. So far, the only RCT enrolling low-risk patients with BAV treated by TAVR is “Notion-2 trial” (NCT02825134). A good practice of patient selection, preprocedural planning, intraprocedural techniques, and the prevention of complications are still prerequisites to achieve good outcomes. Advances in device design and treatment strategies should further improve the results of TAVR in patients with BAV.

## Limitations

There were some limitations in this article. Firstly, the majority of the included studies were not randomized trials in design, neither had core laboratory adjudications. The choice of prosthesis was not randomized but up to the operator's discretion. A significant heterogeneity existed in some analyses. Secondly, although consecutive patients were enrolled, a plenty of articles did not use propensity score matching to eliminate an inherent baseline difference. Patients with BAV with different anatomical phenotypes and a varying degree of calcification might lead to disparate outcomes but was not further delineated in many studies. Thirdly, the absence of long-term survival and hemodynamic results of patients with BAV makes it difficult to explore some questions of interest, e.g., THV durability. Fourthly, patients with BAV in our included population were not representable enough for all symptomatic patients with BAV because those who were not suitable for TAVR had already been excluded. Moreover, some studies only enrolled patients with BAV using BE or SE THVs alone were not included. Both resulted in a selection bias in our report. Fifthly, although we have been cautious in overlapping population, it may still present in our result when single-center data were reported both alone and among multicenter studies. Sixthly, we divided the Lotus valve into self-expandable THVs when analyzing although they are mechanically expandable valves academically. However, the sample size is small (about 11 patients). Seventhly, in 37 of 49 funnel plots of our meta-analyses, the number of original studies was <10, leading to insufficient power of test of the funnel plots. Finally, we only screened the articles in English.

## Conclusion

Despite higher risks of conversion to SAVR, the need of a second valve, moderate or severe PVL, device failure, AKI, stroke and new PPI, TAVR seems to be a viable option for selected patients with bicuspid severe AS, which had a potential benefit of 1-year survival, especially among lower surgical risk population using new-generation devices. Larger randomized studies were needed to guide candidate selection and verified the durable performance of THVs in the BAV population.

## Data Availability Statement

The original contributions presented in the study are included in the article/Supplementary Material, further inquiries can be directed to the corresponding author/s.

## Author Contributions

YZ, T-YX, Y-ML, and MC participated in the design of the study. YZ, T-YX, and Y-ML were responsible for the coordination and acquisition of the data. YZ and T-YX performed the statistical analysis. All authors contributed to the preparation, critical review, and approved the final manuscript.

## Conflict of Interest

MC and YF are consultants/proctors of Venus MedTech, MicroPort, and Peijia Medical. The remaining authors declare that the research was conducted in the absence of any commercial or financial relationships that could be construed as a potential conflict of interest.

## Publisher's Note

All claims expressed in this article are solely those of the authors and do not necessarily represent those of their affiliated organizations, or those of the publisher, the editors and the reviewers. Any product that may be evaluated in this article, or claim that may be made by its manufacturer, is not guaranteed or endorsed by the publisher.
