# Supplementary material for: Patients With Bicuspid Aortic Stenosis Undergoing Transcatheter Aortic Valve Replacement: A Systematic Review and Meta-Analysis
Source: Front Cardiovasc Med. 2022 Mar 16;9:794850. doi: 10.3389/fcvm.2022.794850 (PMC8965870; doi:10.3389/fcvm.2022.794850)
Supplement: Supplementary file 1 [file Data_Sheet_1.docx]

**Supplementary File**

Figure 1. Original records of meta-analysis of comparisons between **BAV and TAV** patients.

1. In-hospital mortality

1.1 Conversion to SAVR

All THVs


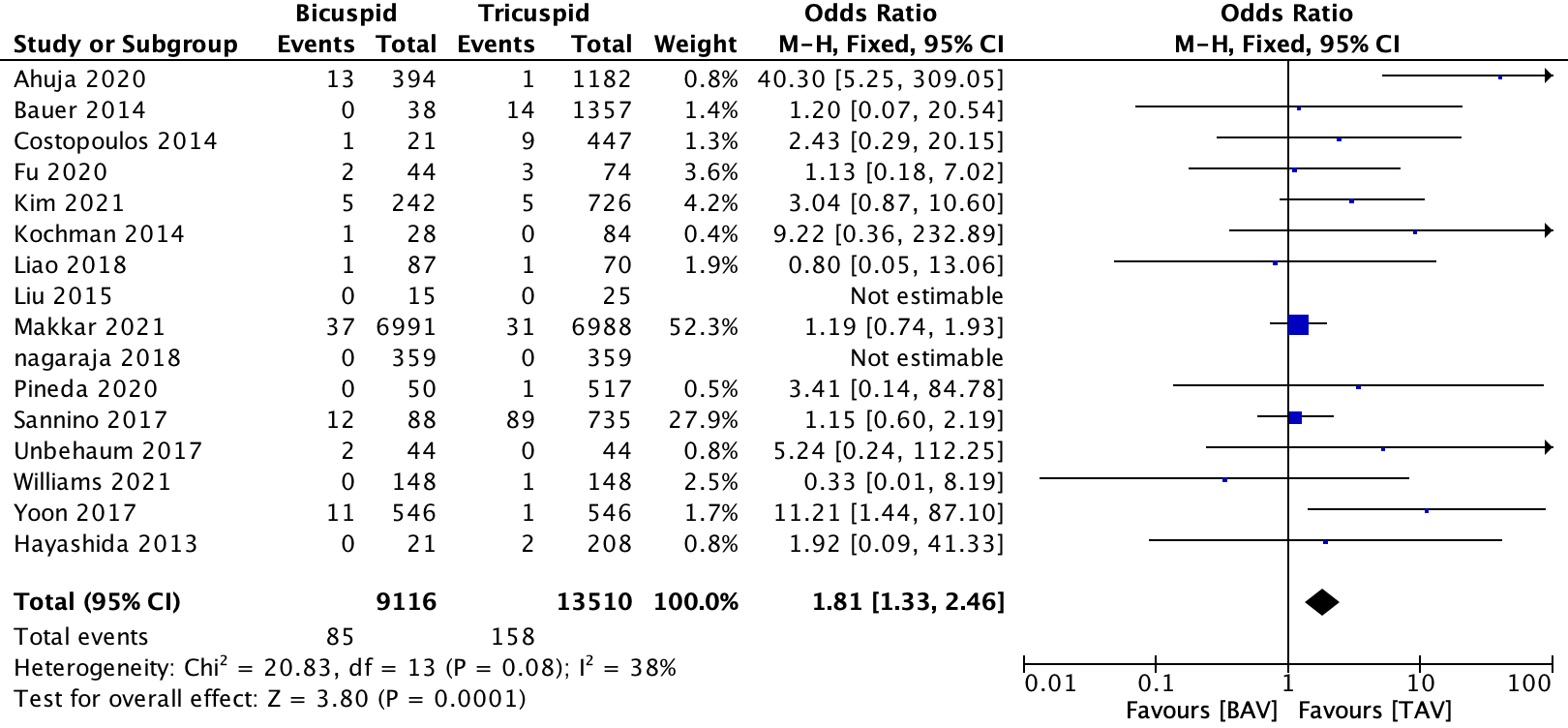


Early generation THVs


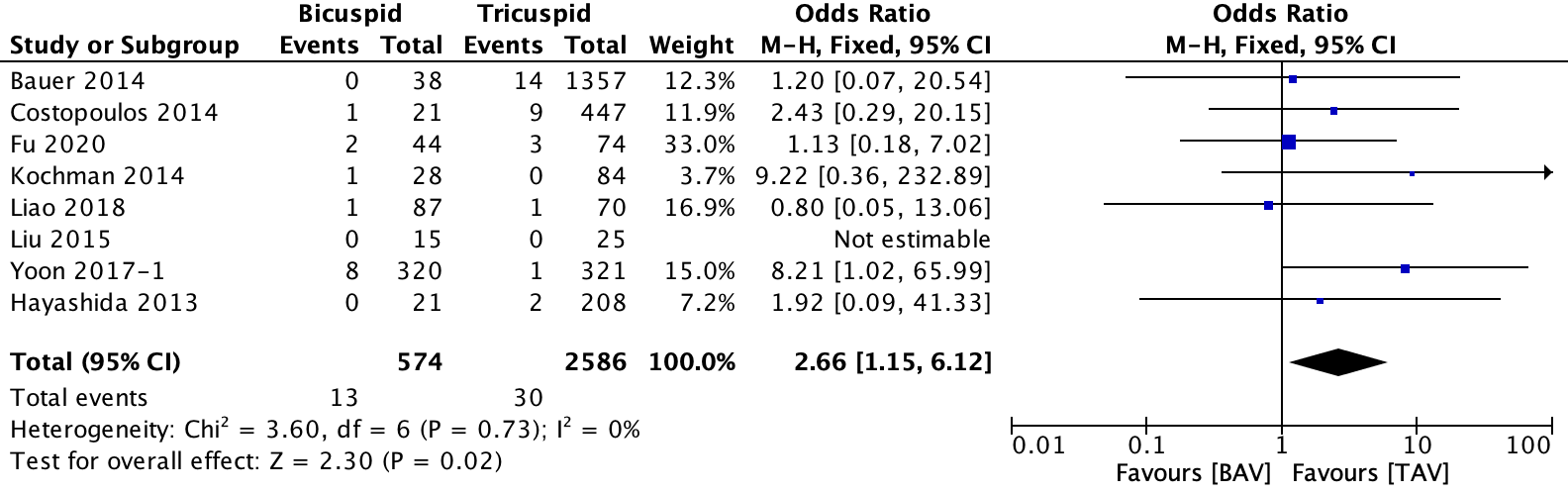


New generation THVs


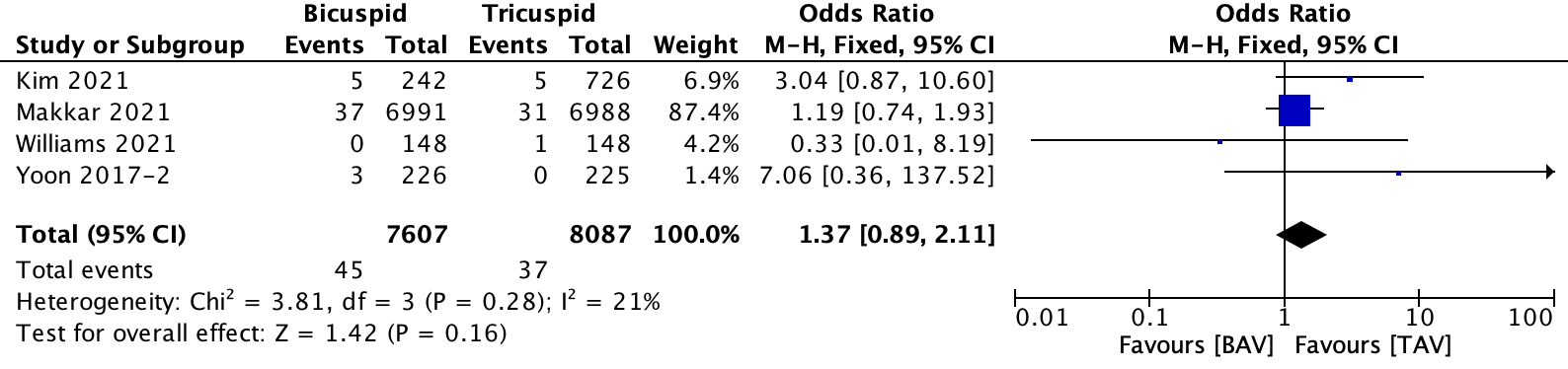


1.2 Coronary obstruction

All THVs


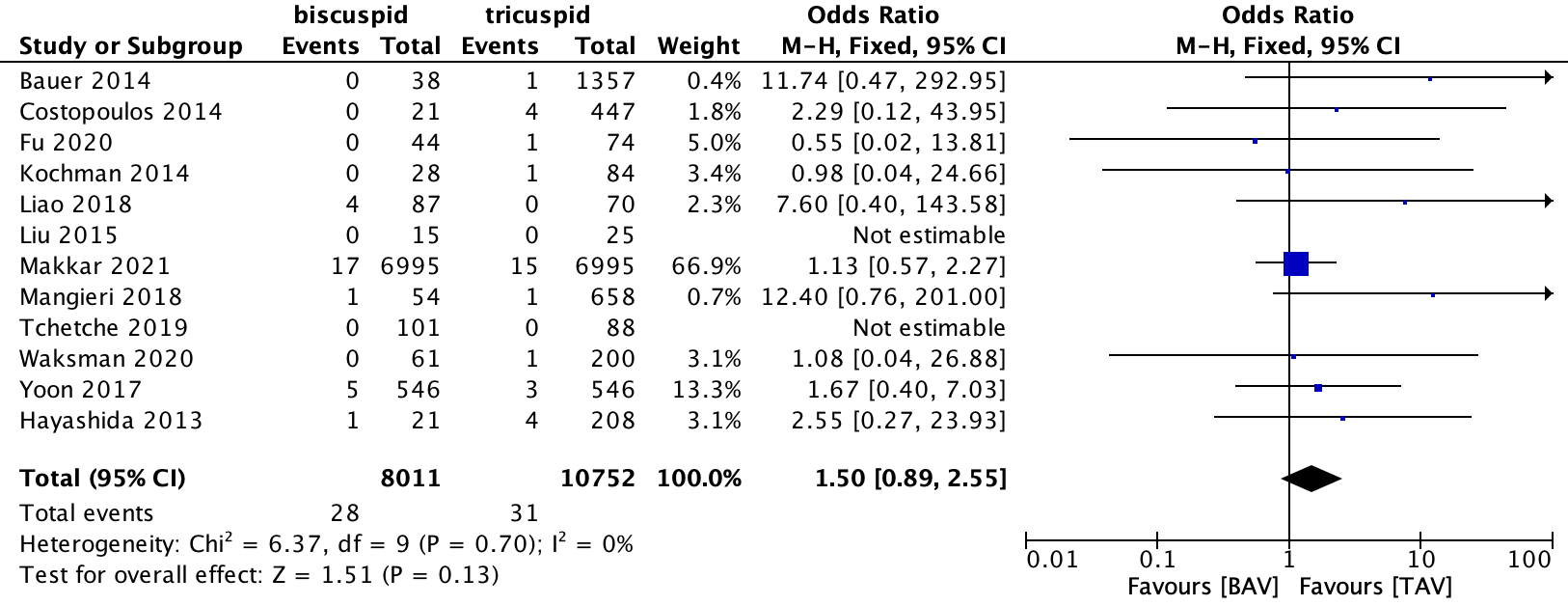


Early generation THVs


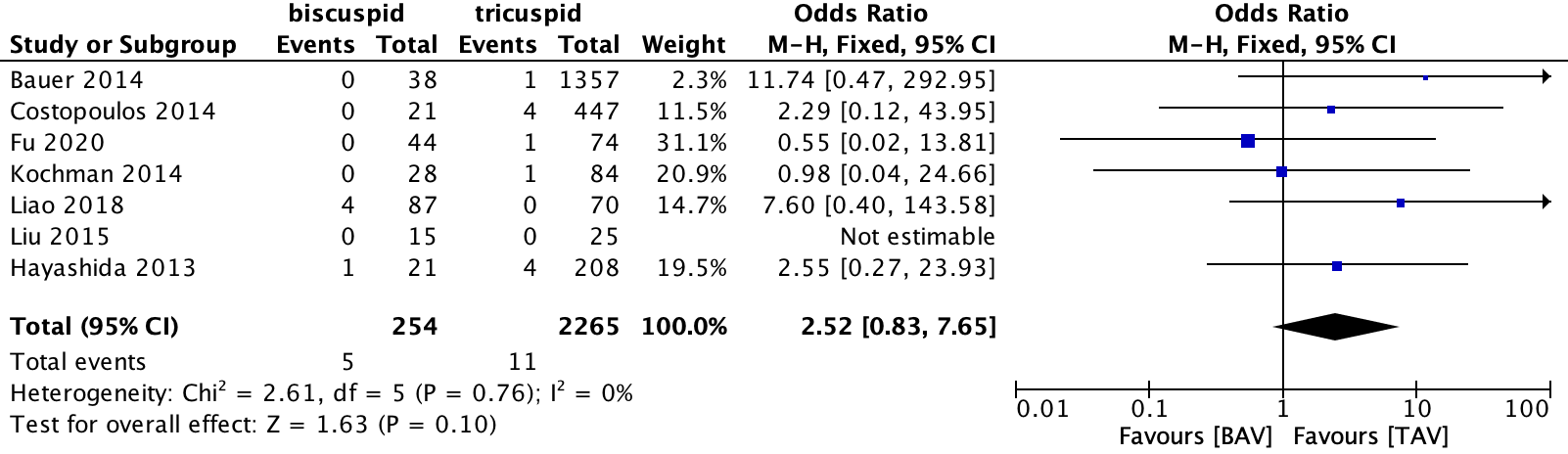


New generation THVs


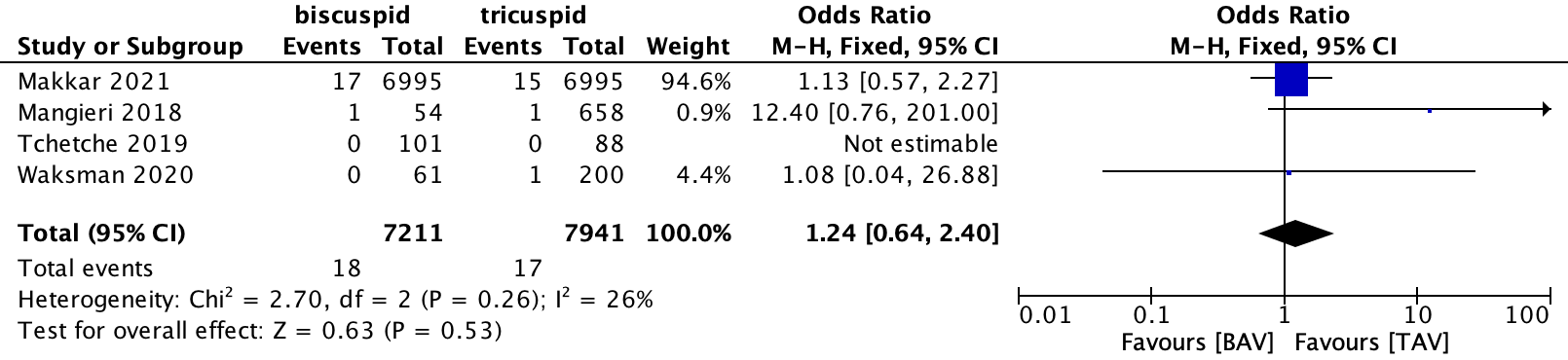


1.3 The need of a second valve

All THVs


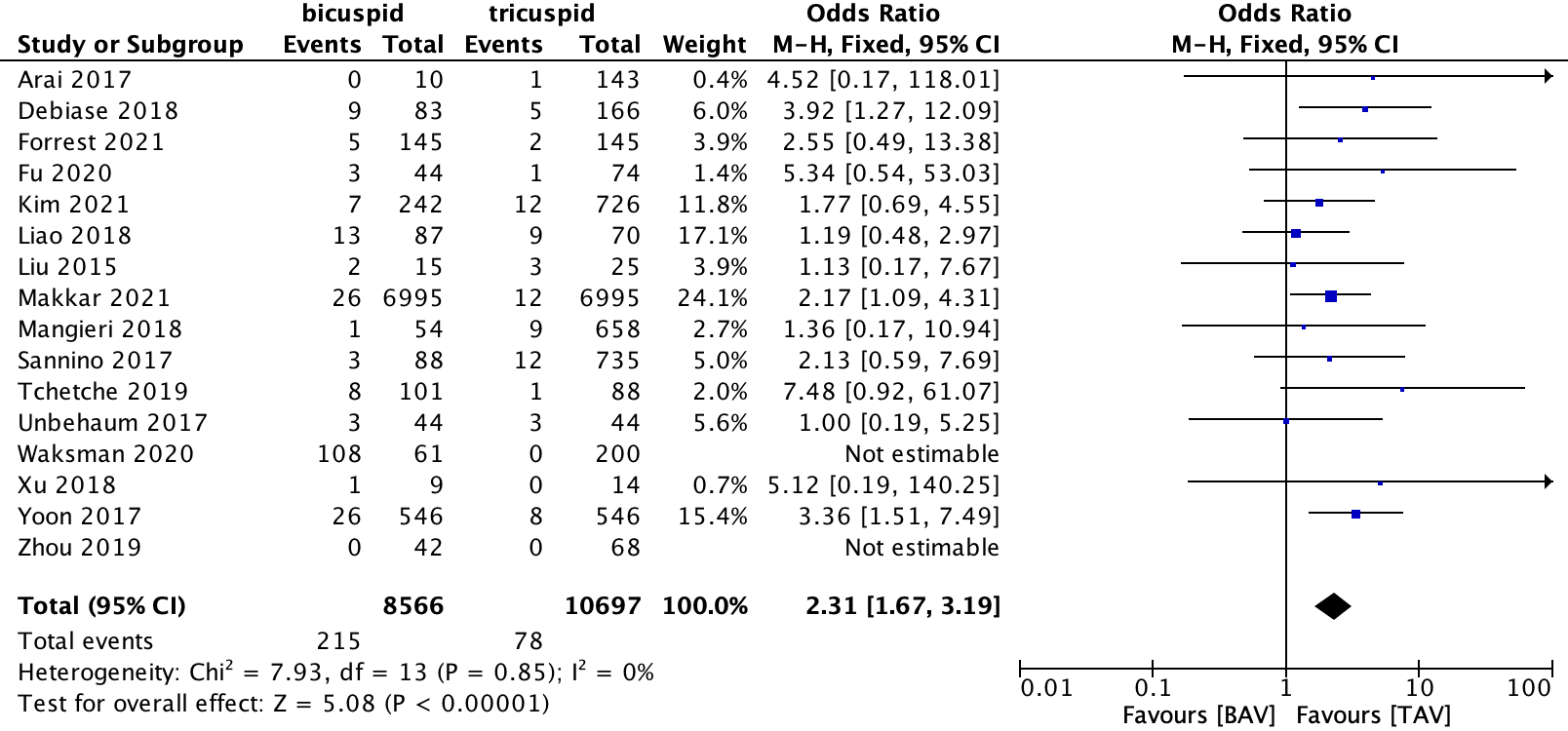


Early generation THVs


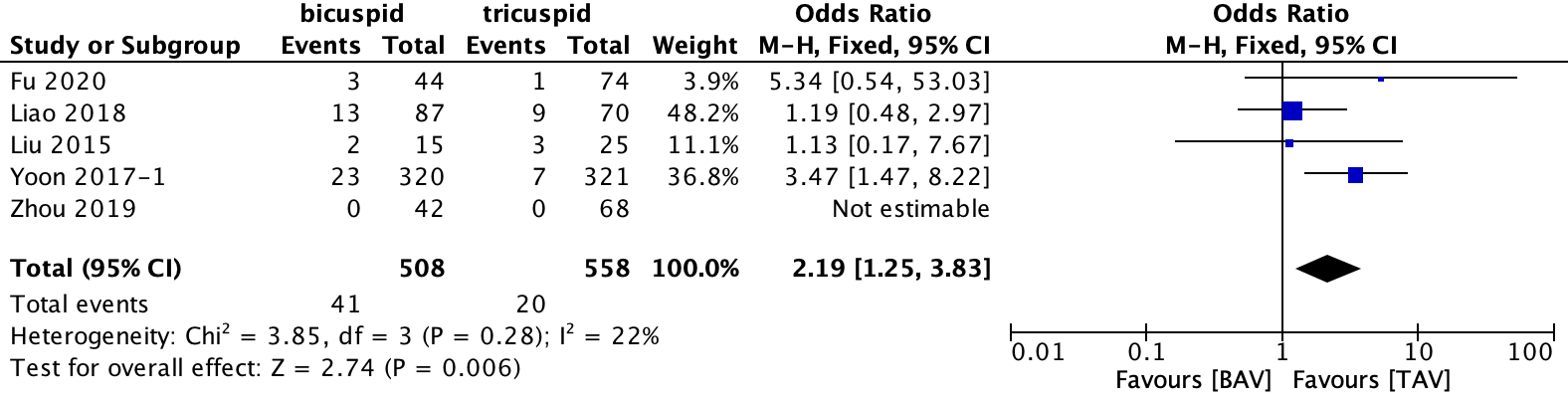


New generation THVs


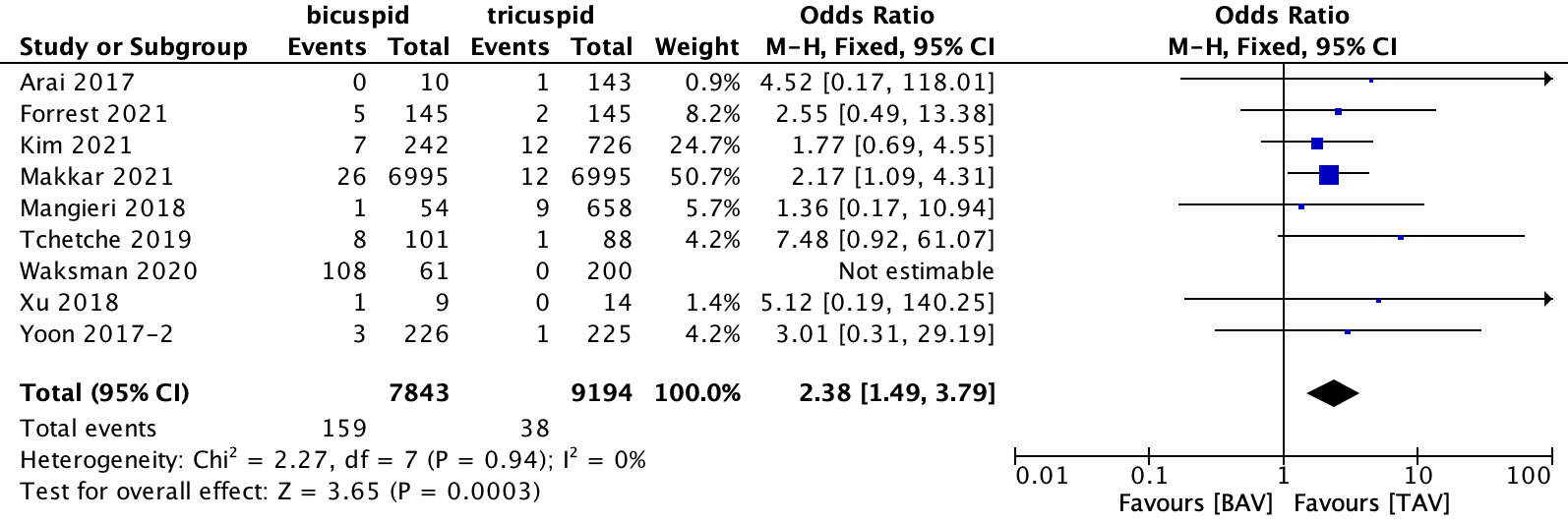


1.4 Moderate or Severe PVL

All THVs


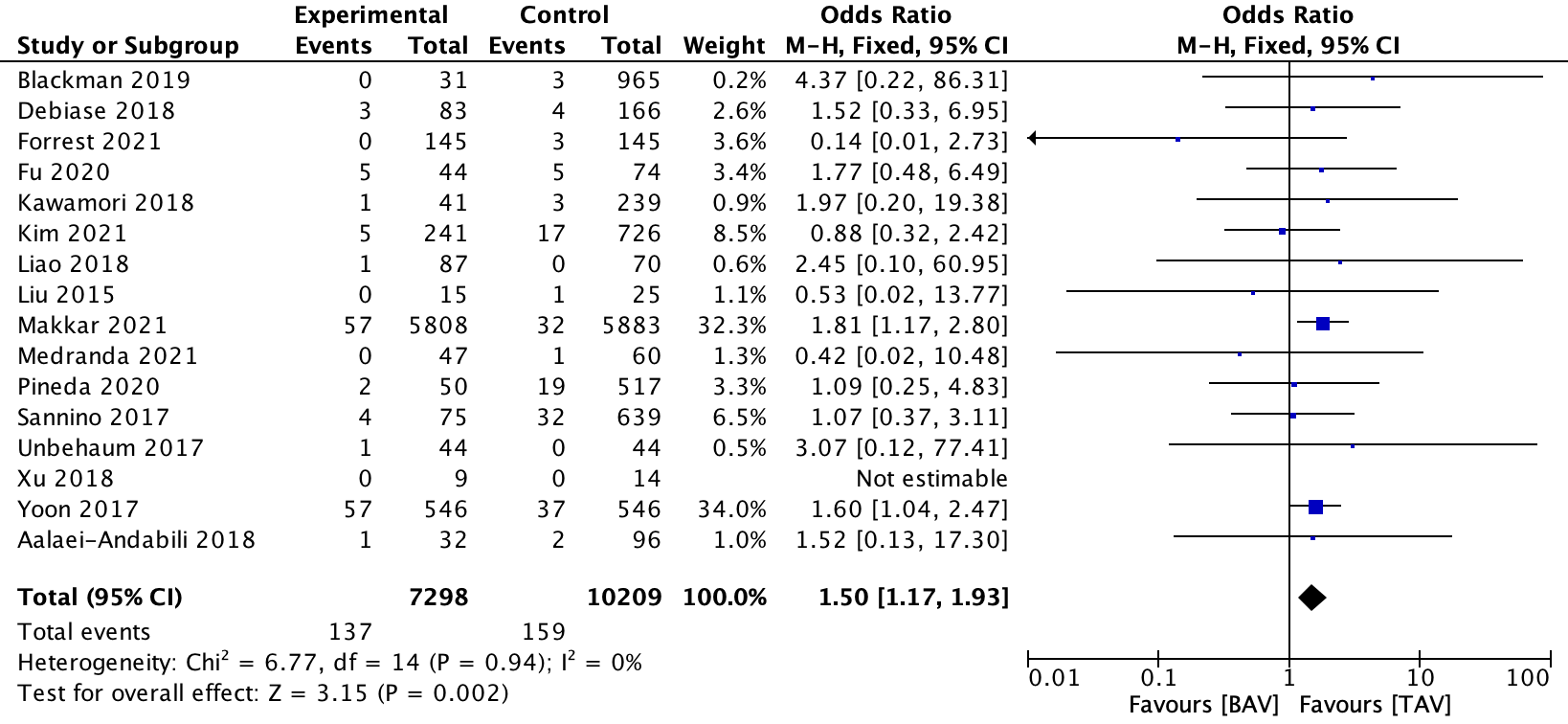


Early generation THVs


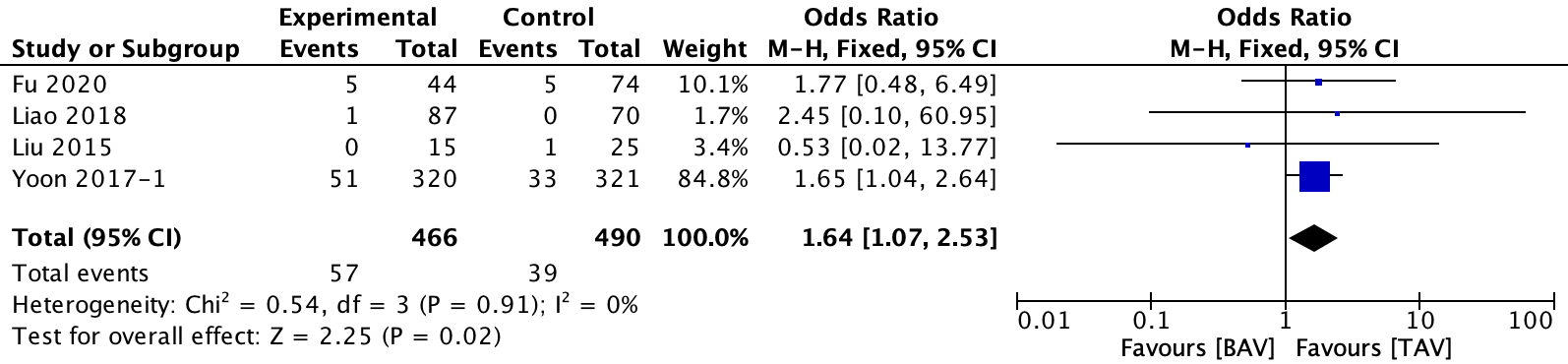


New generation THVs


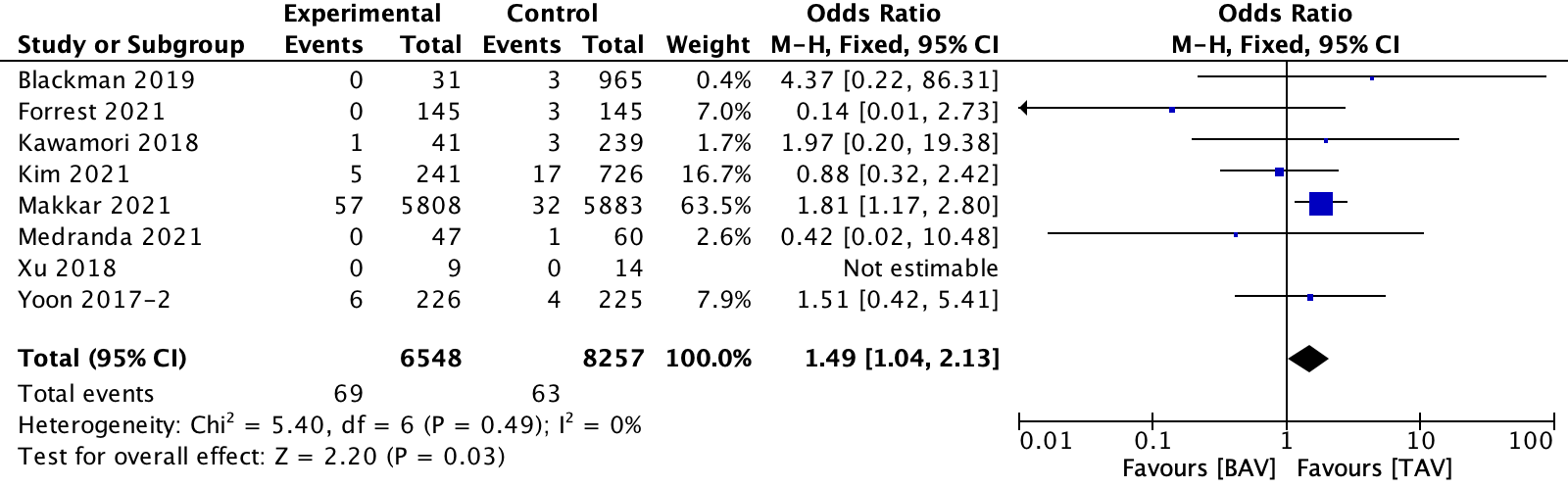


1.5 Major vascular complication

All THVs


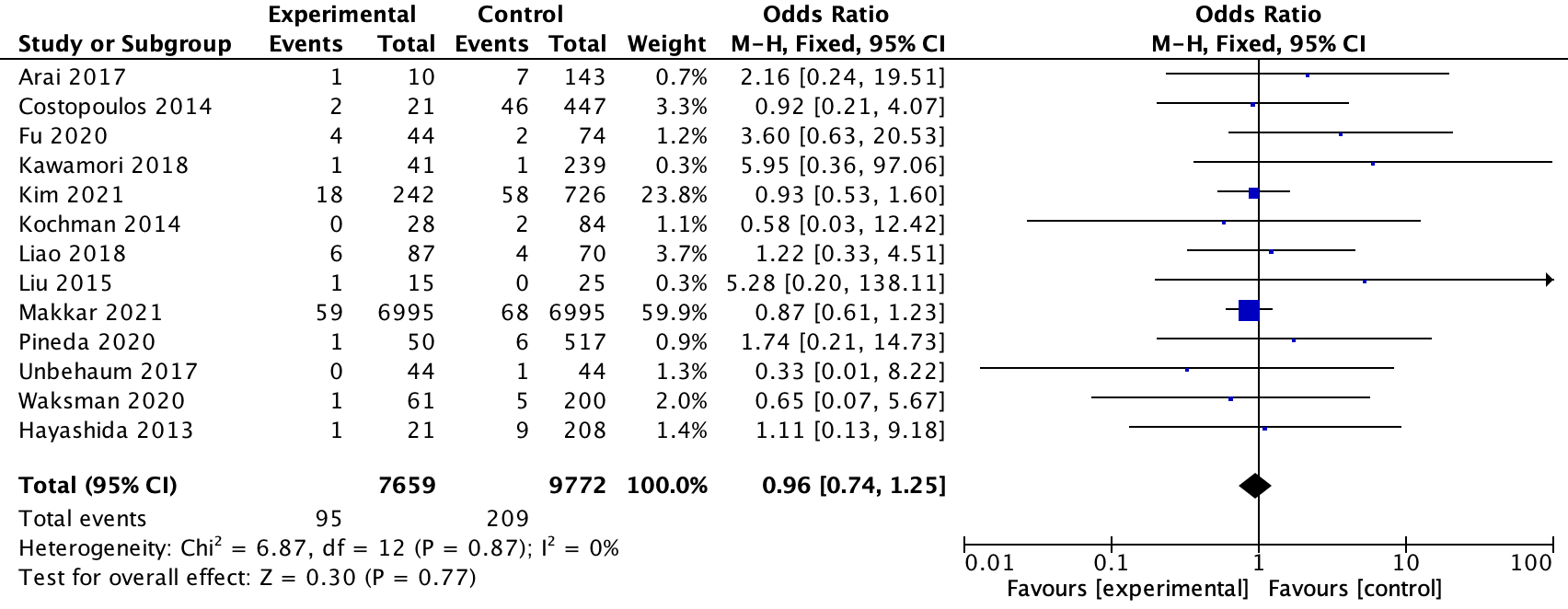


Early generation THVs


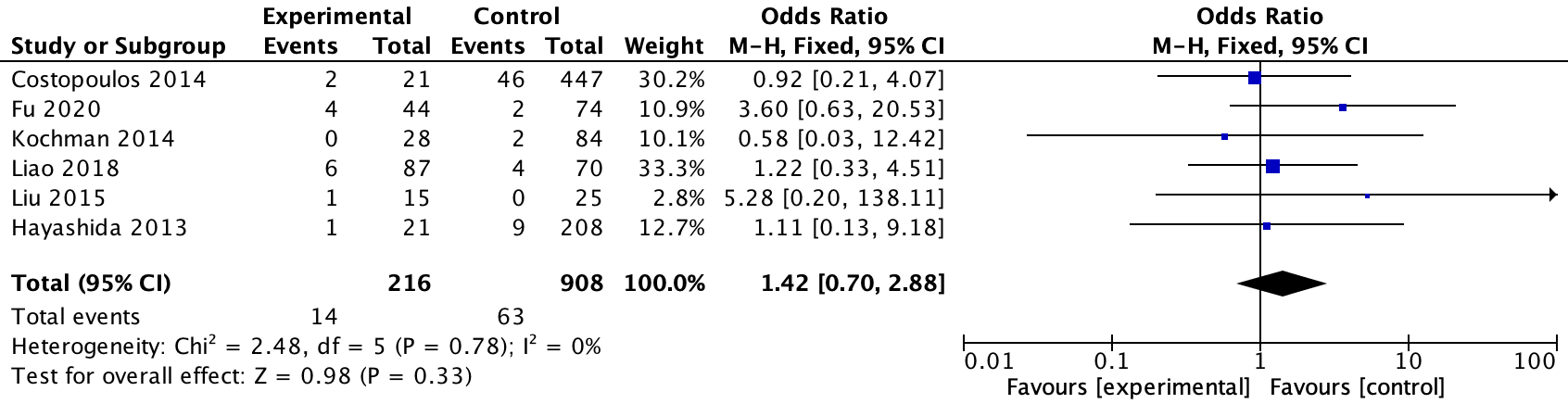


New generation THVs


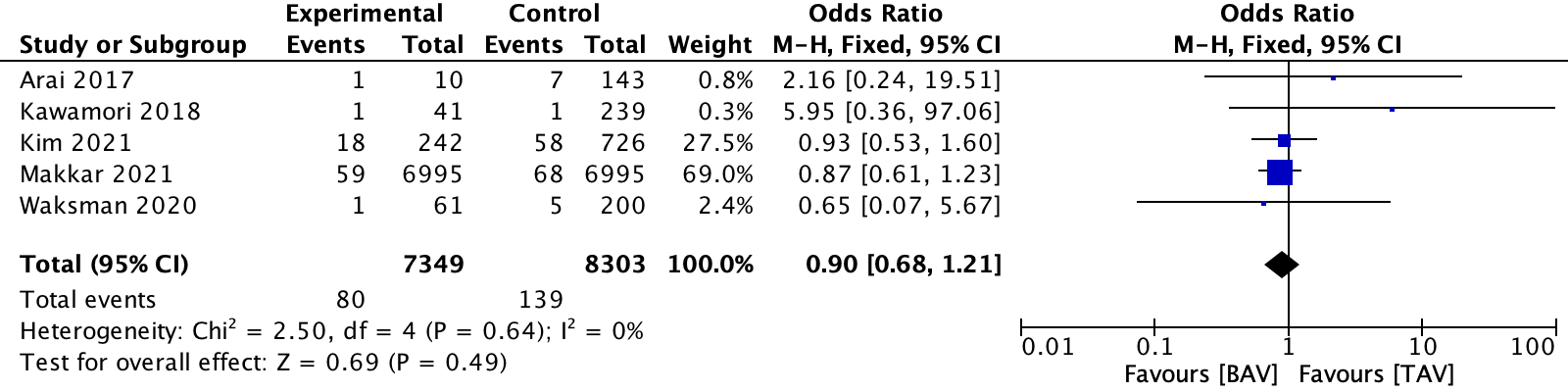


1.6 Device failure

All THVs


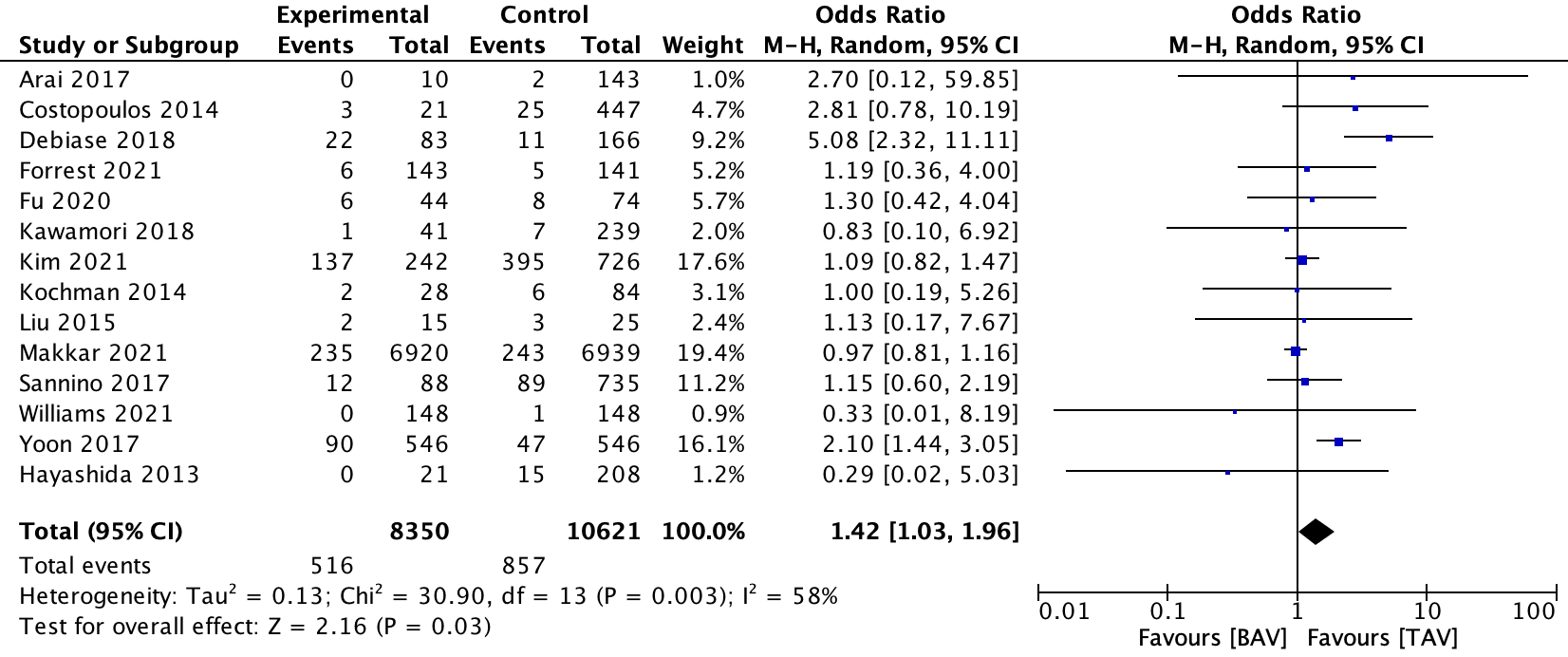


Early generation THVs


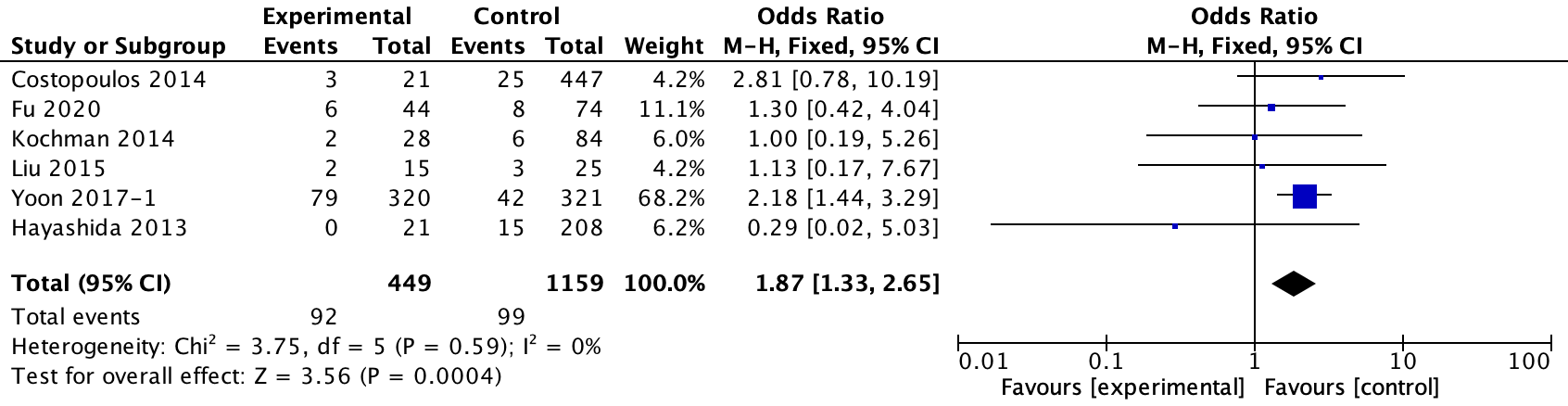


New generation THVs


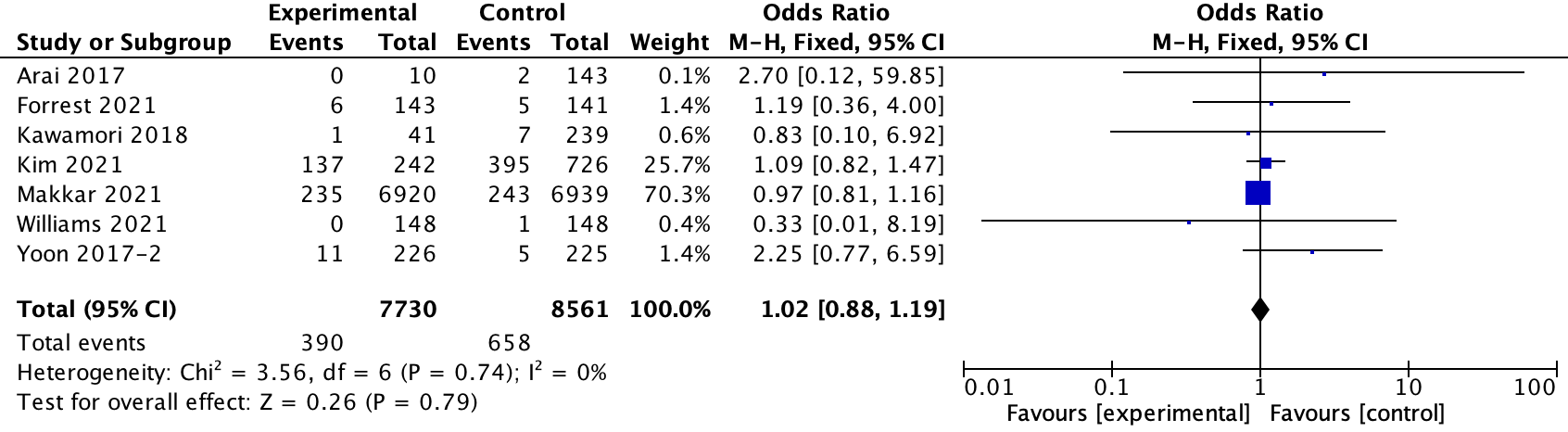


1.7 AKI

All THVs


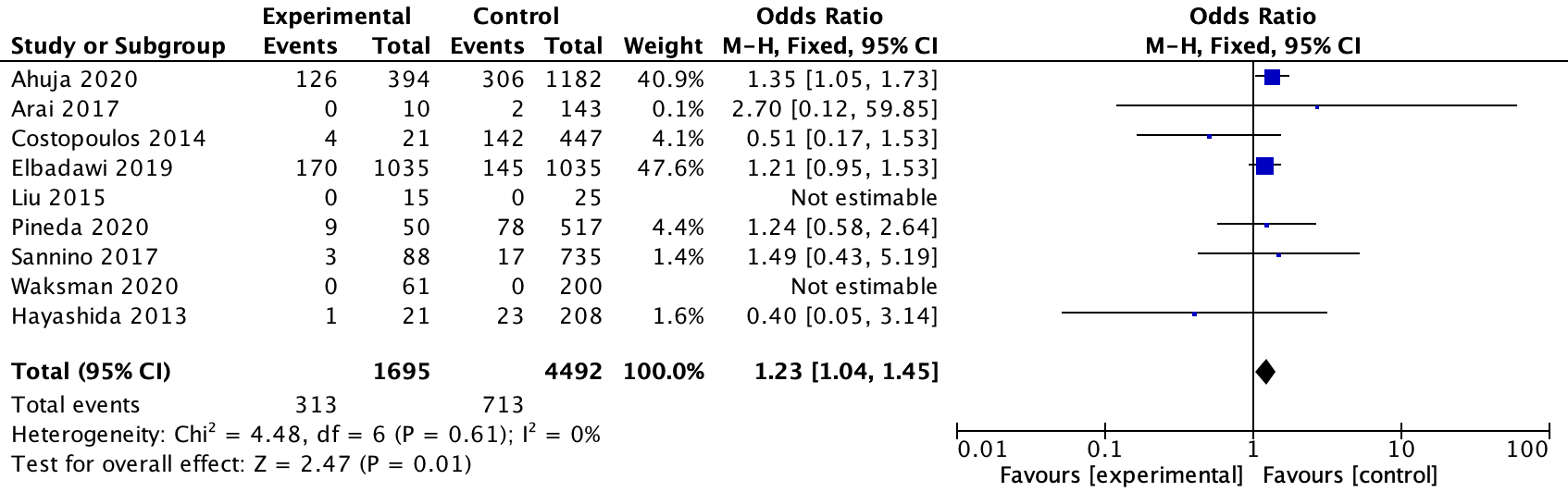


Early generation THVs


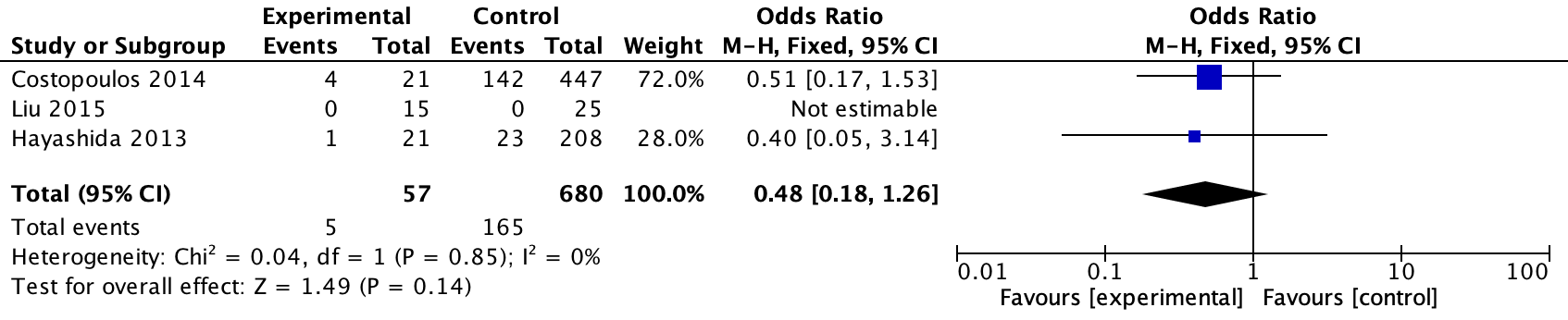


New generation THVs


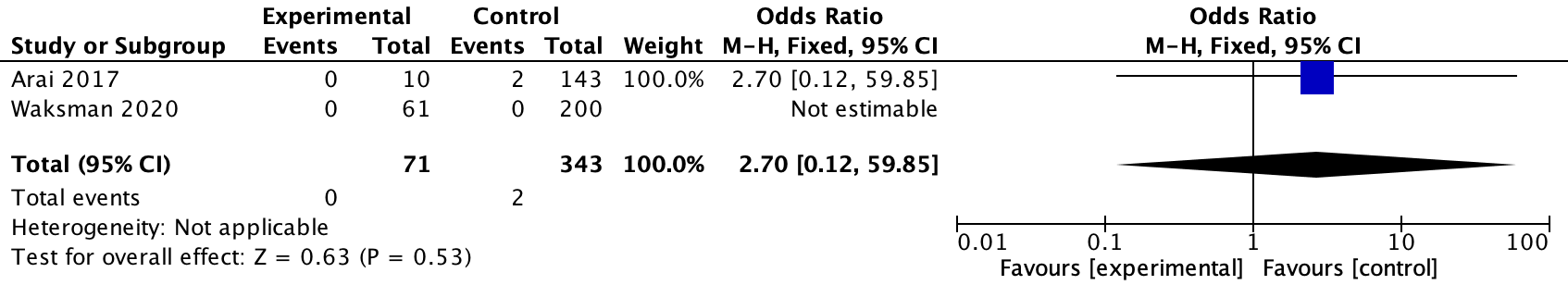


1.8 Annulus rupture

All THVs


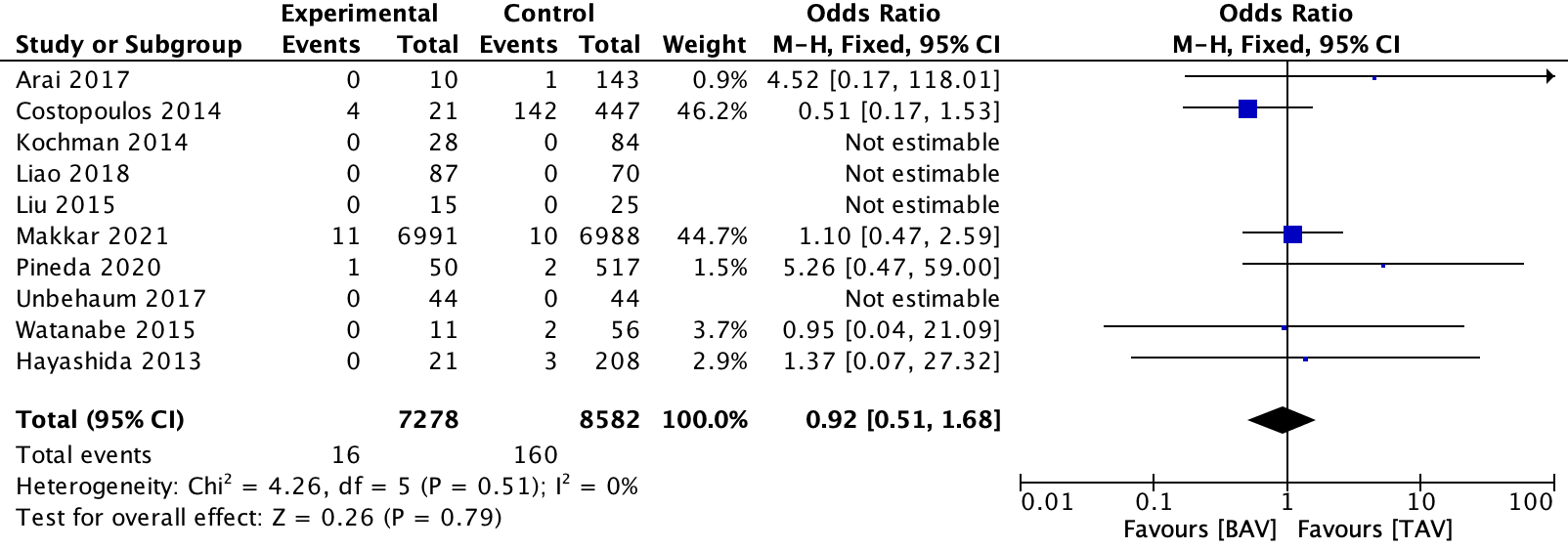


Early generation THVs


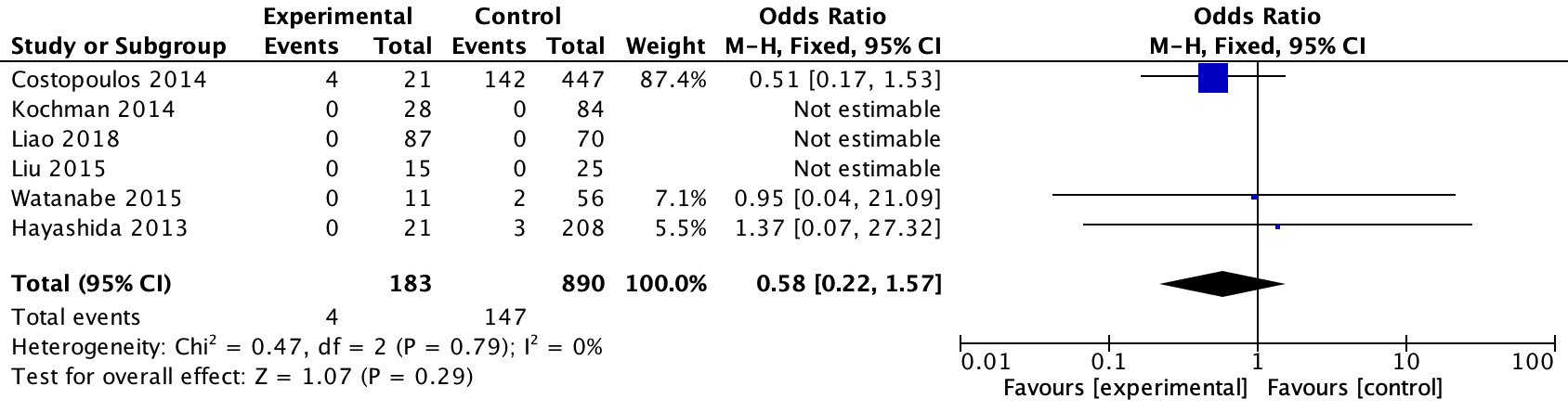


New generation THVs


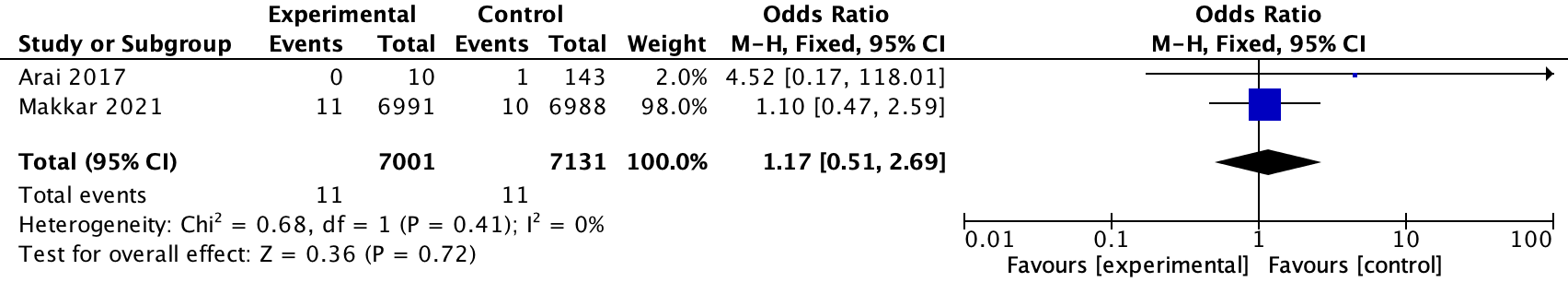


1.9 Life-threatening or major bleeding

All THVs


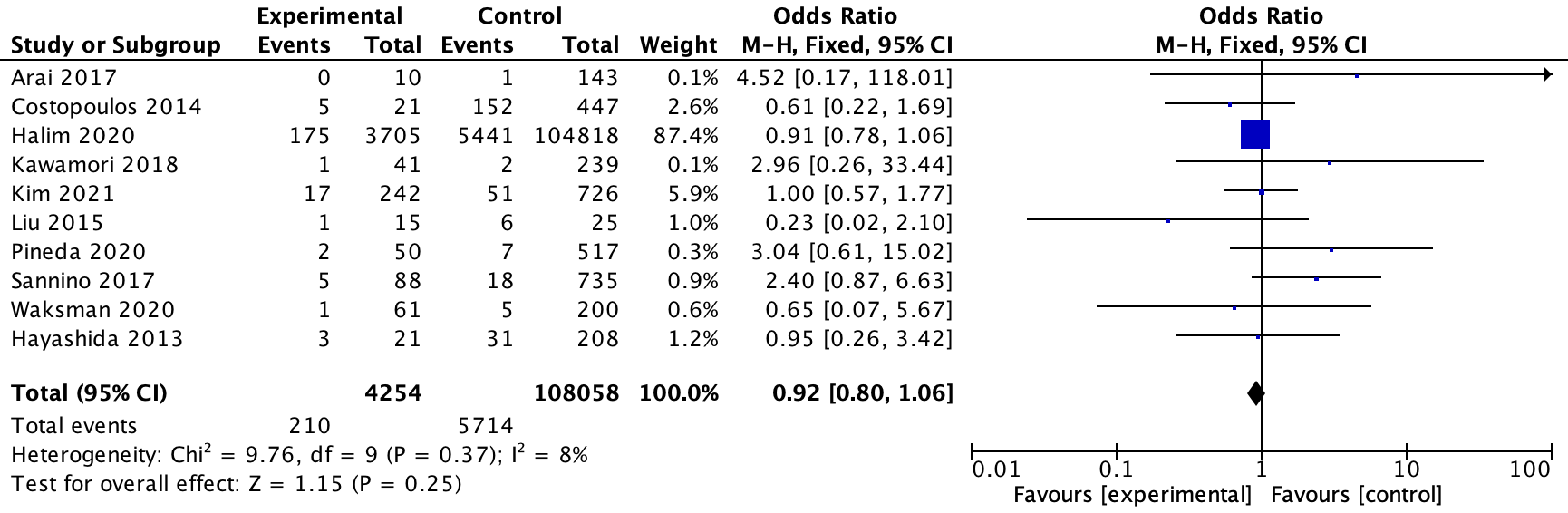


Early generation THVs


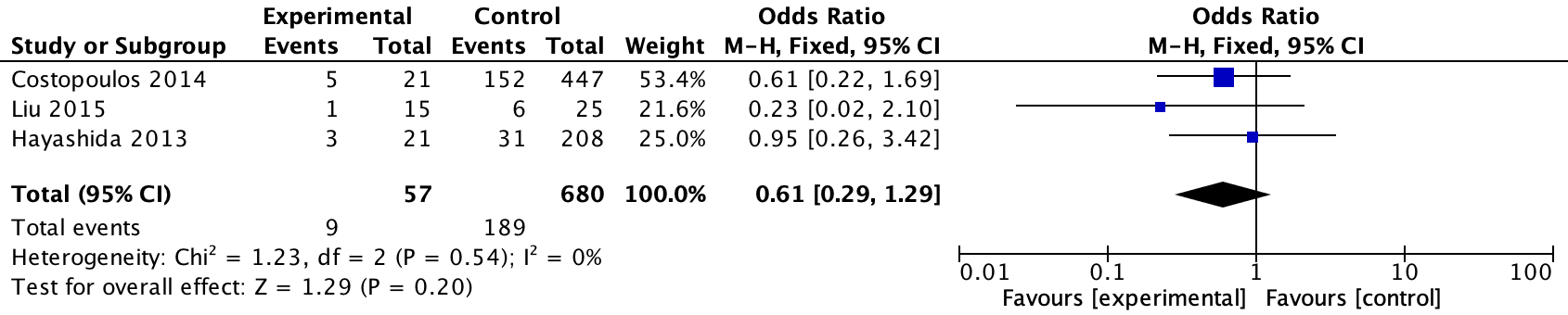


New generation THVs


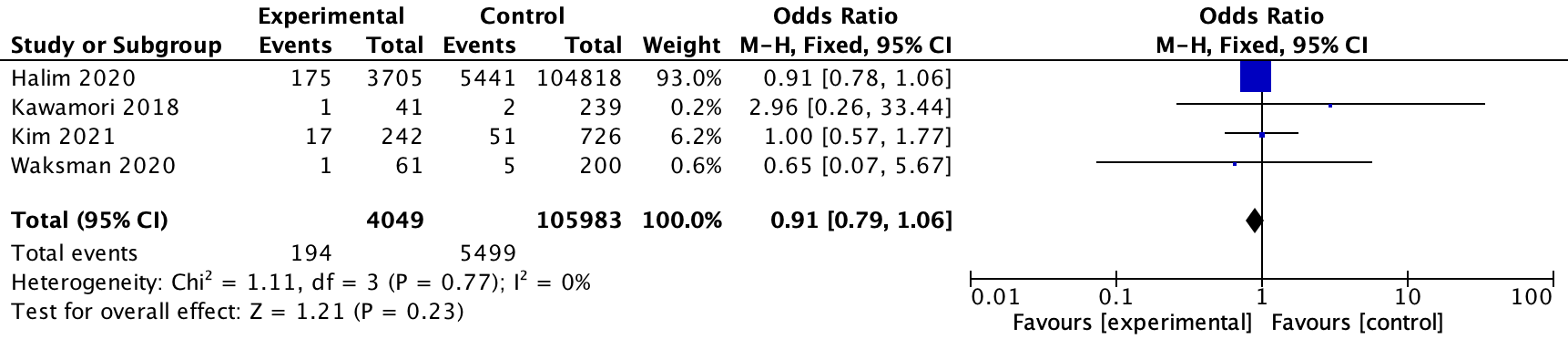


1.10 MI

All THVs


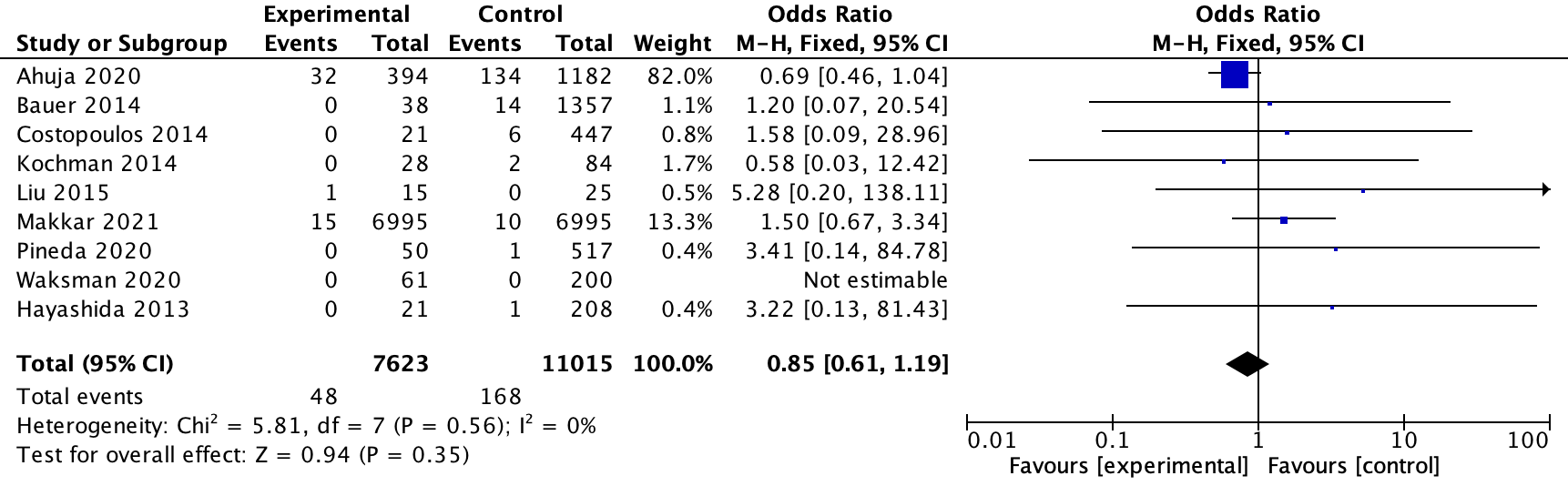


Early generation THVs


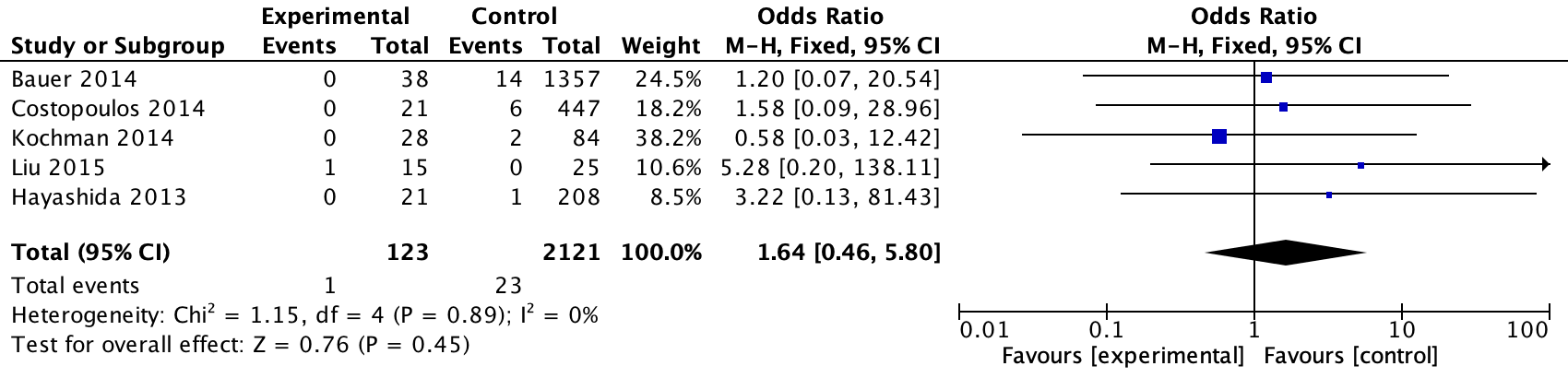


New generation THVs


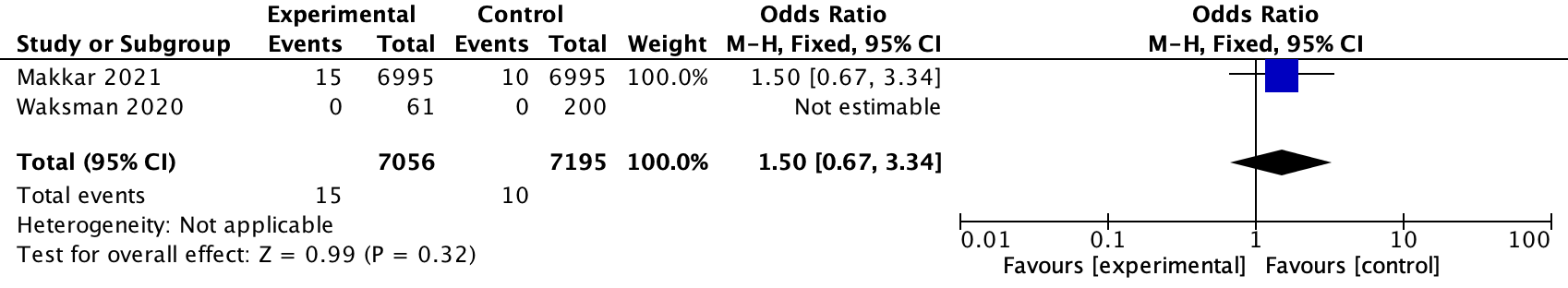


1.11 NO-AF

All THVs


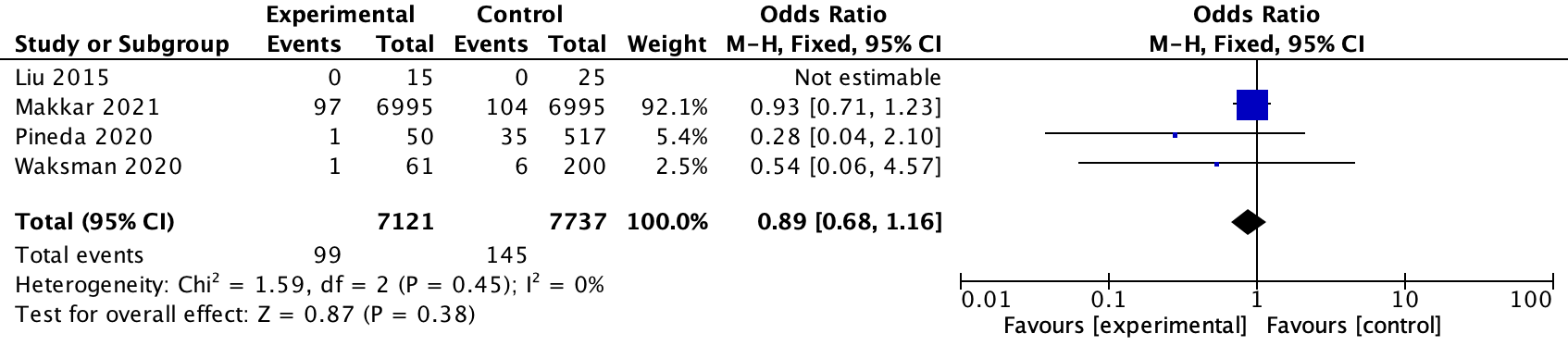


Early generation THVs


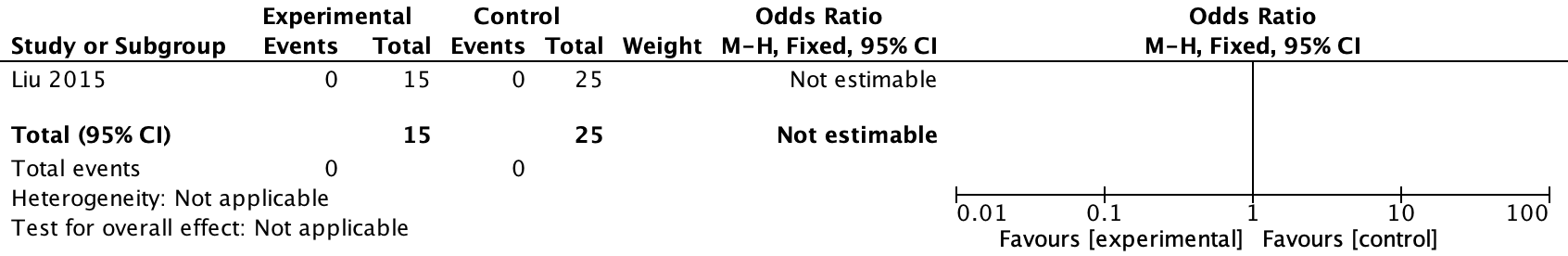


New generation THVs


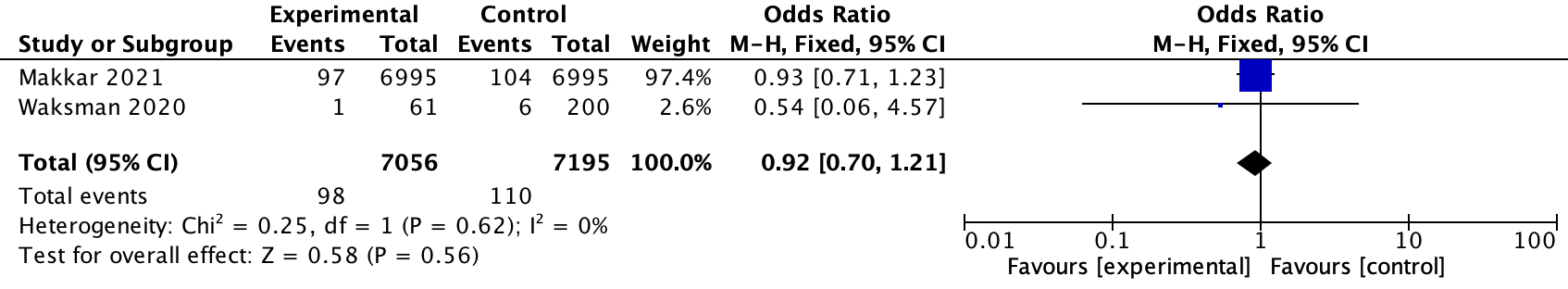


1.12 New PPI

All THVs


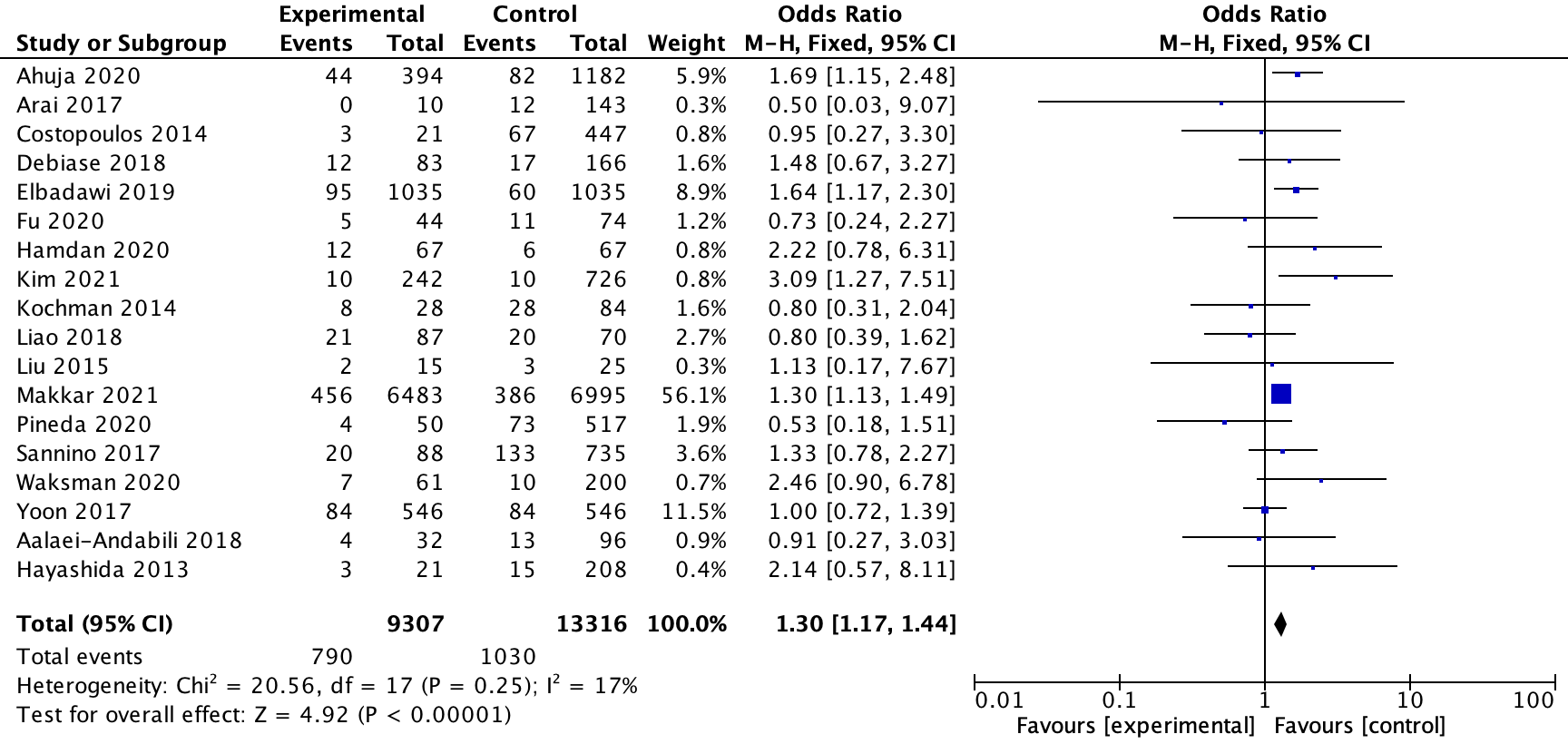


Early generation THVs


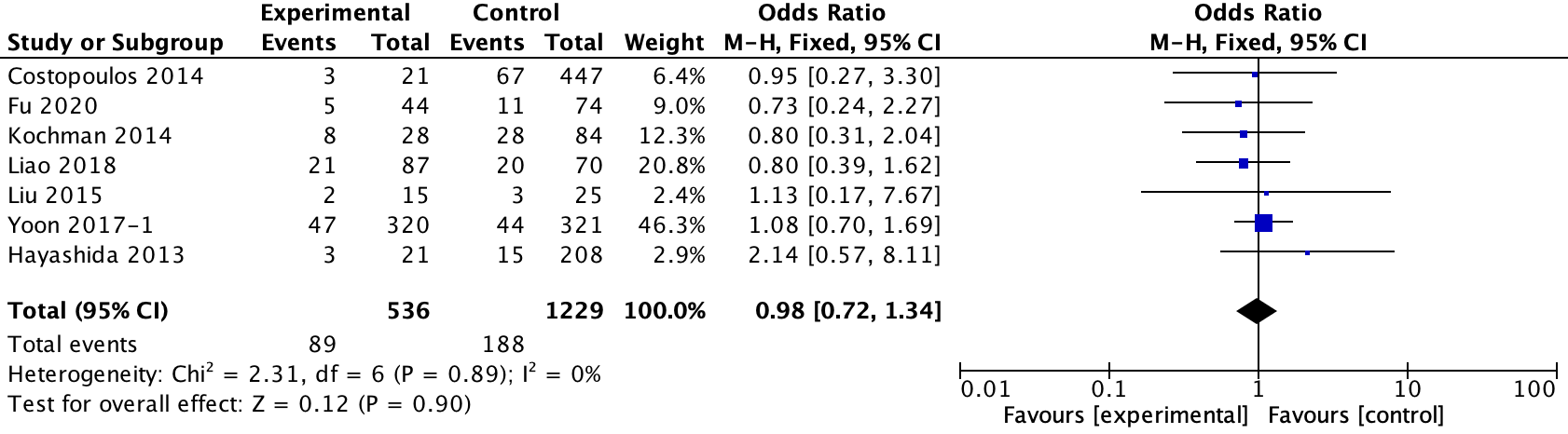


New generation THVs


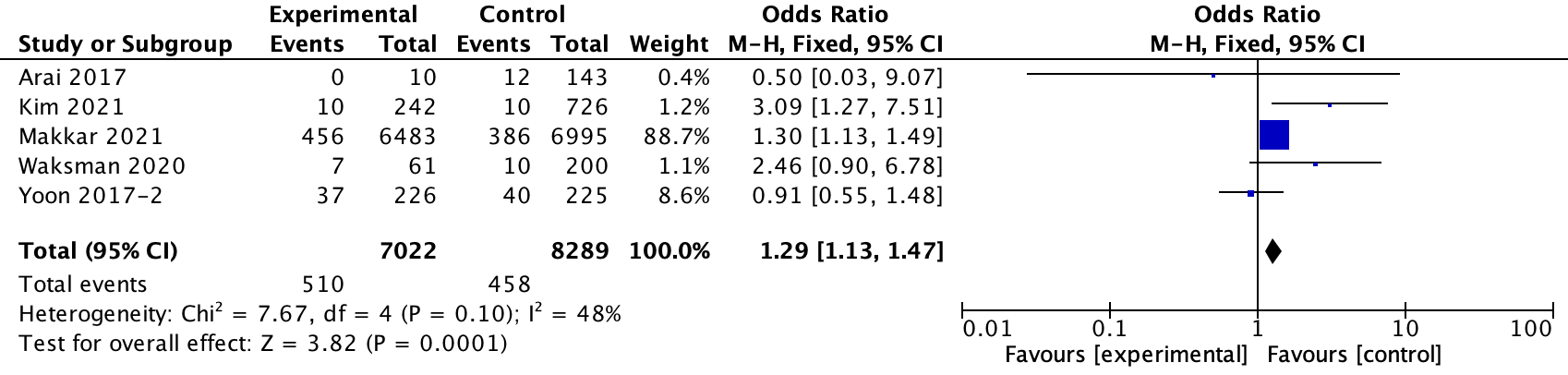


1.13 Stroke

All THVs


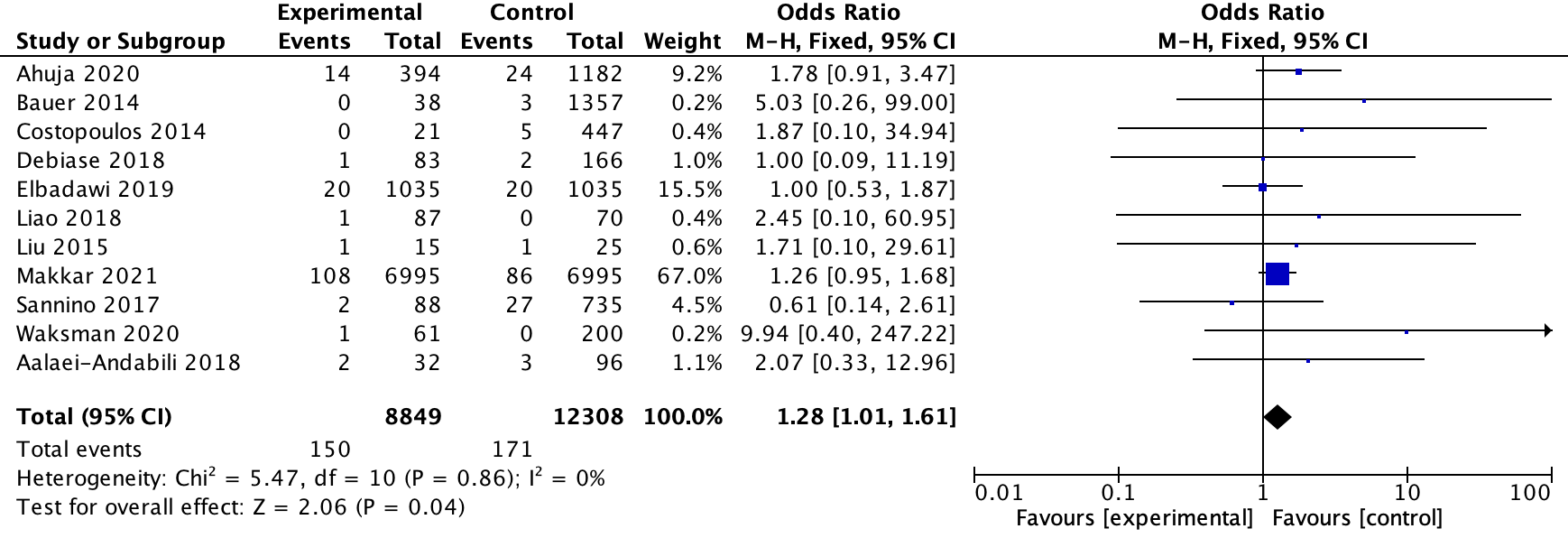


Early generation THVs


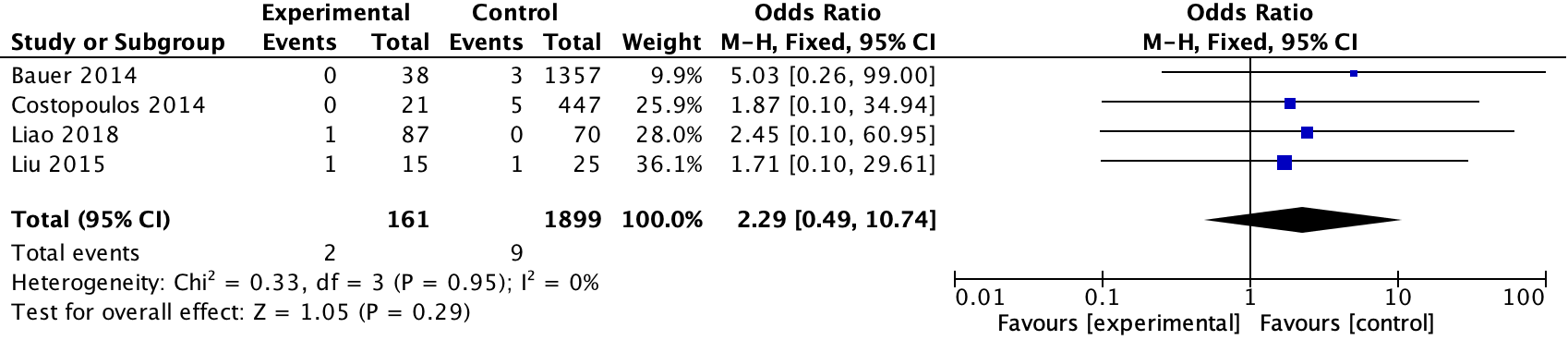


New generation THVs


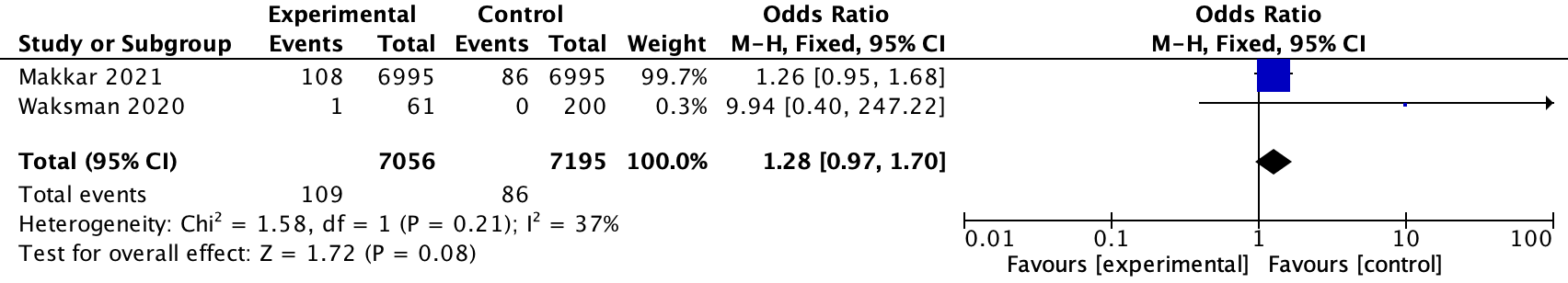


1.14 In-hospital all-cause mortality

All THVs


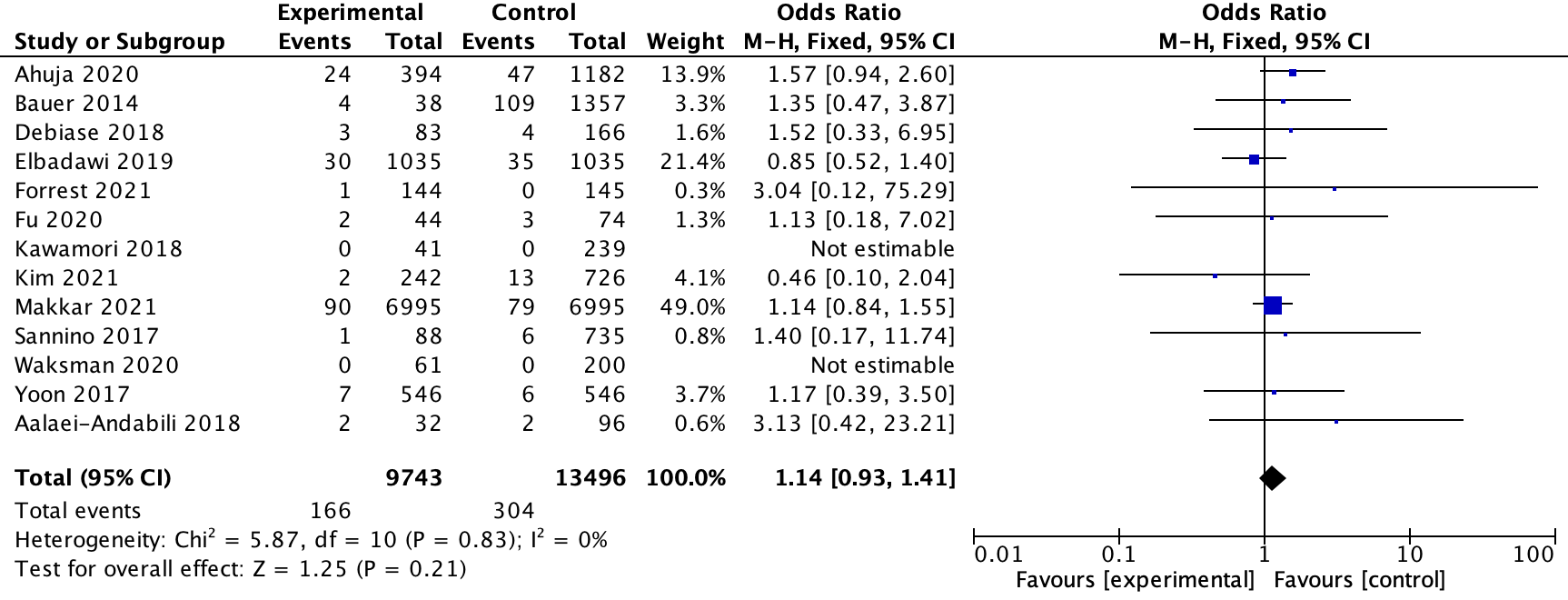


Early generation THVs


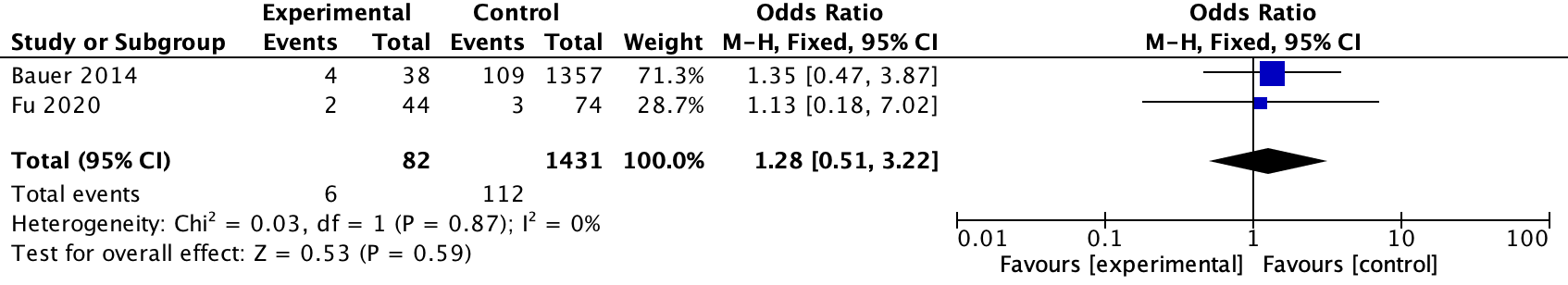


New generation THVs


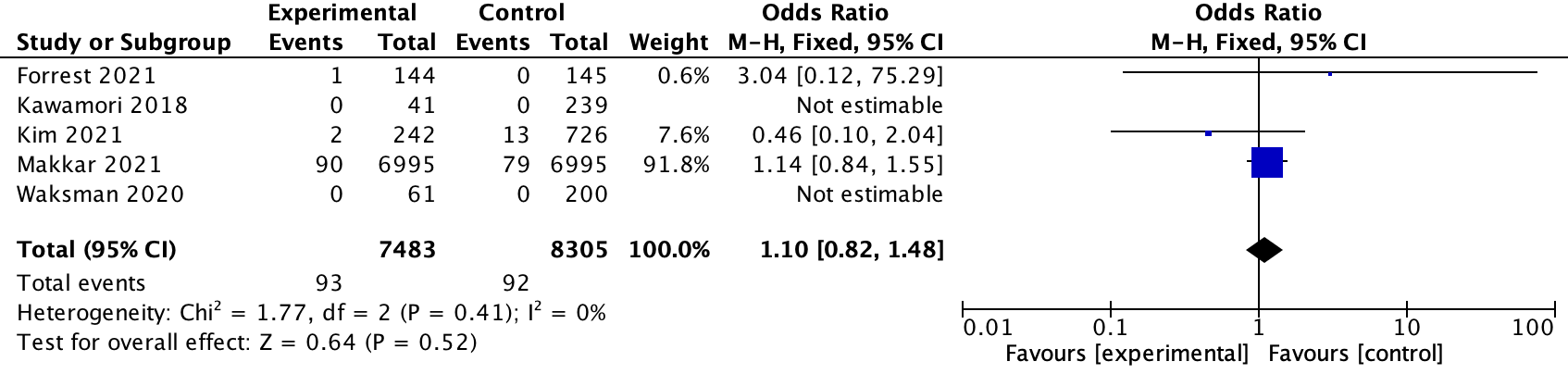


2. 30-day follow-up

2.1 Moderate or Severe PVL

All THVs


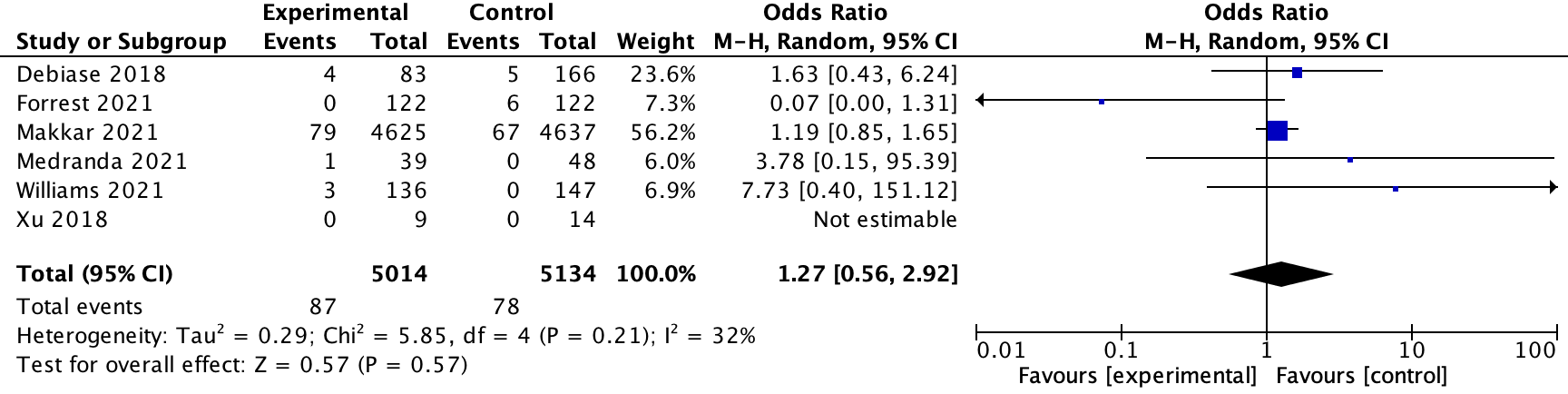


Early generation THVs

NA

New generation THVs


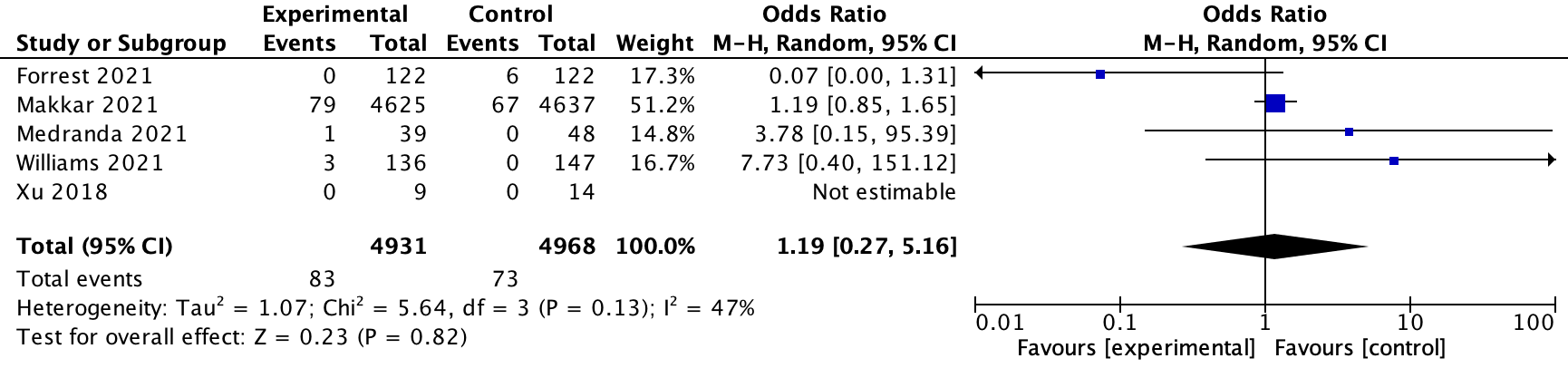


2.2 Stroke

All THVs


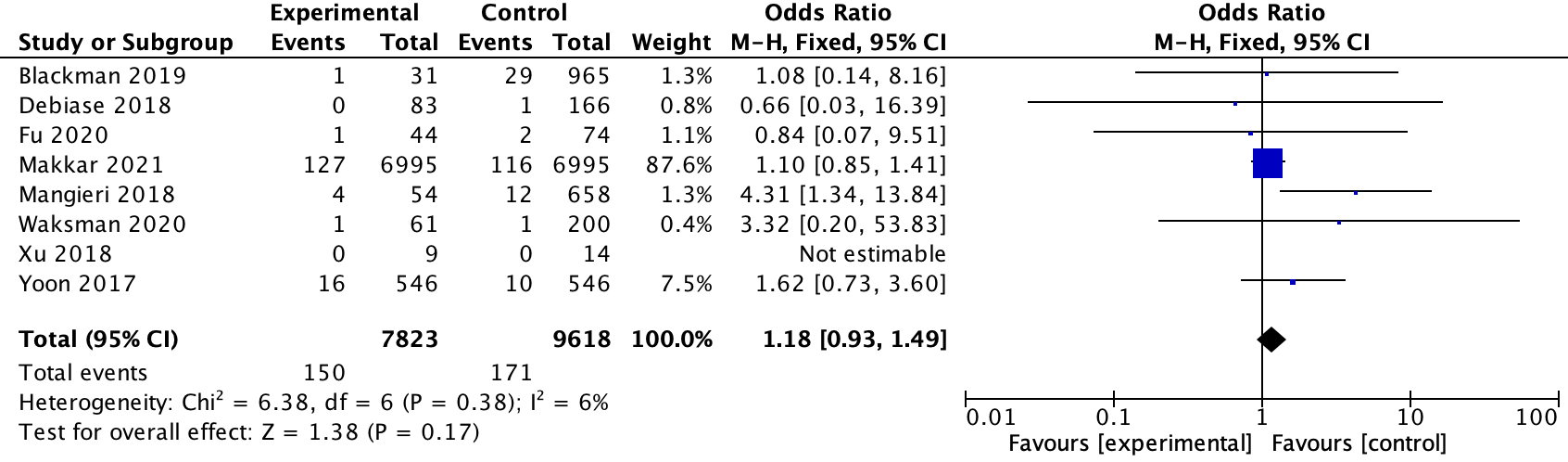


Early generation THVs


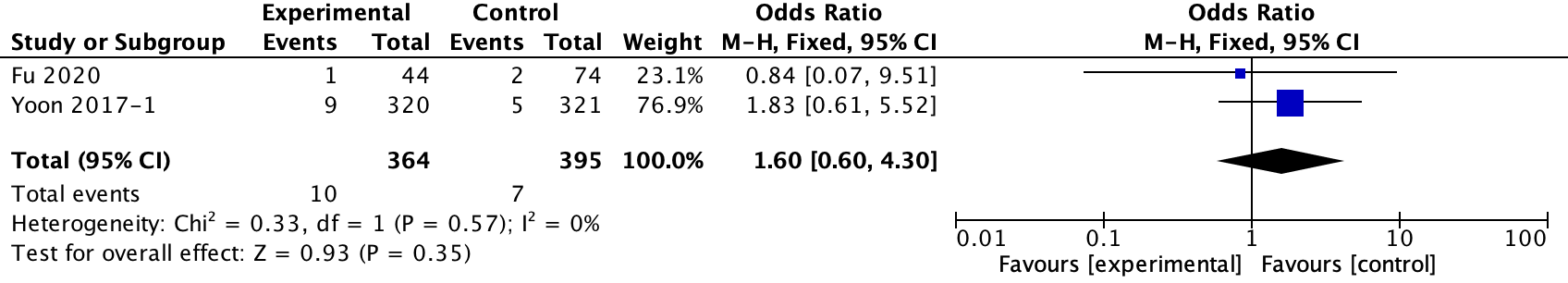


New generation THVs


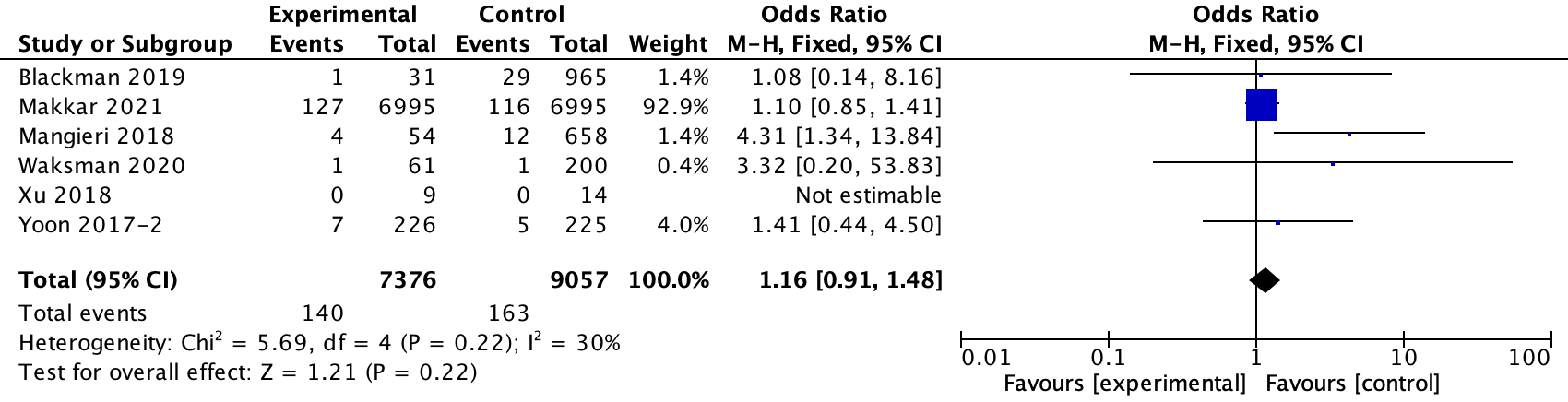


2.3 New PPI

All THVs


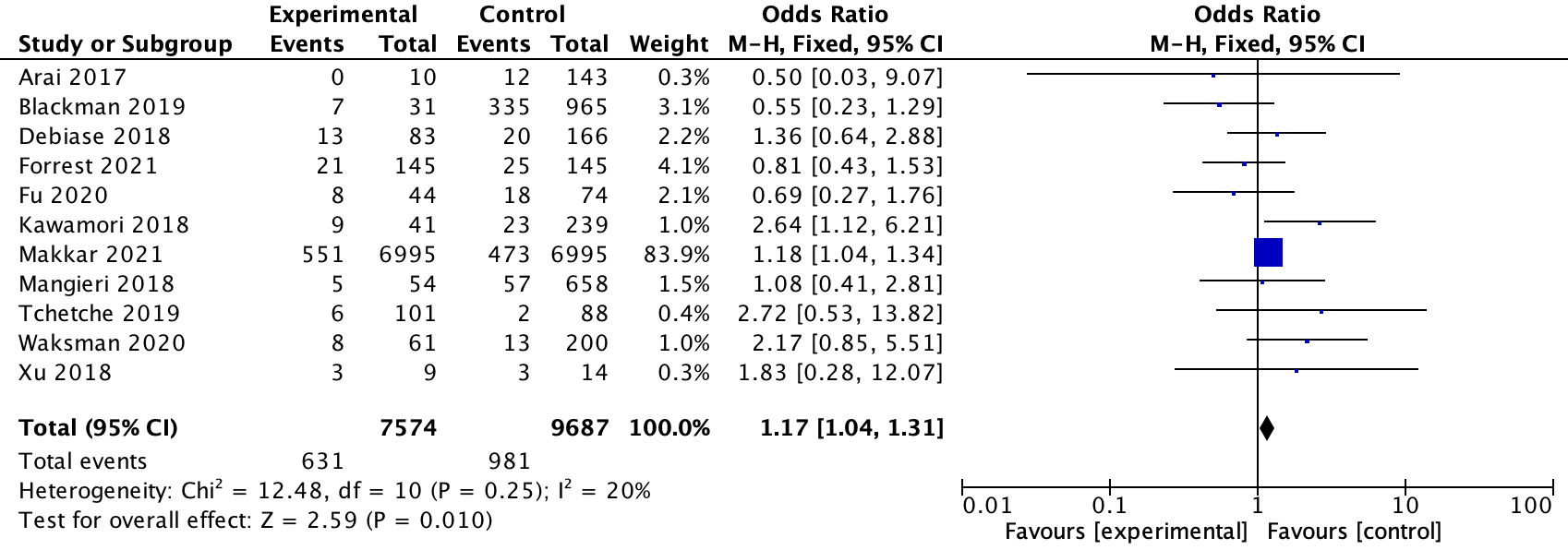


Early generation THVs


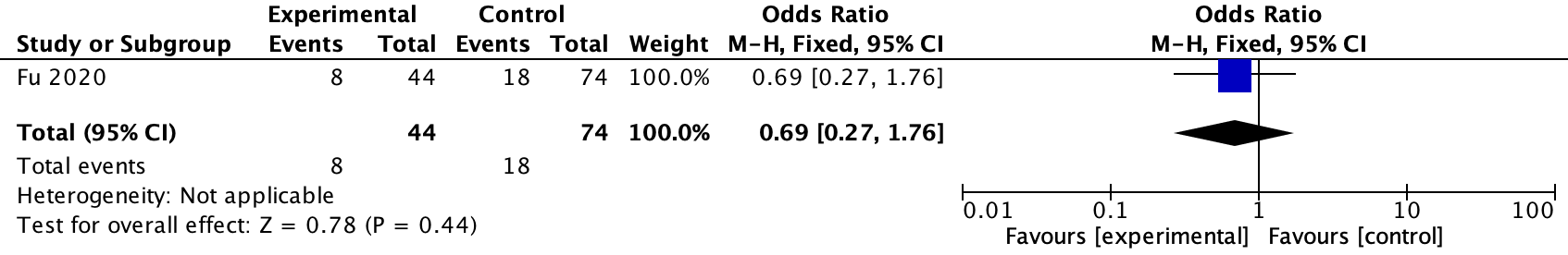


New generation THVs


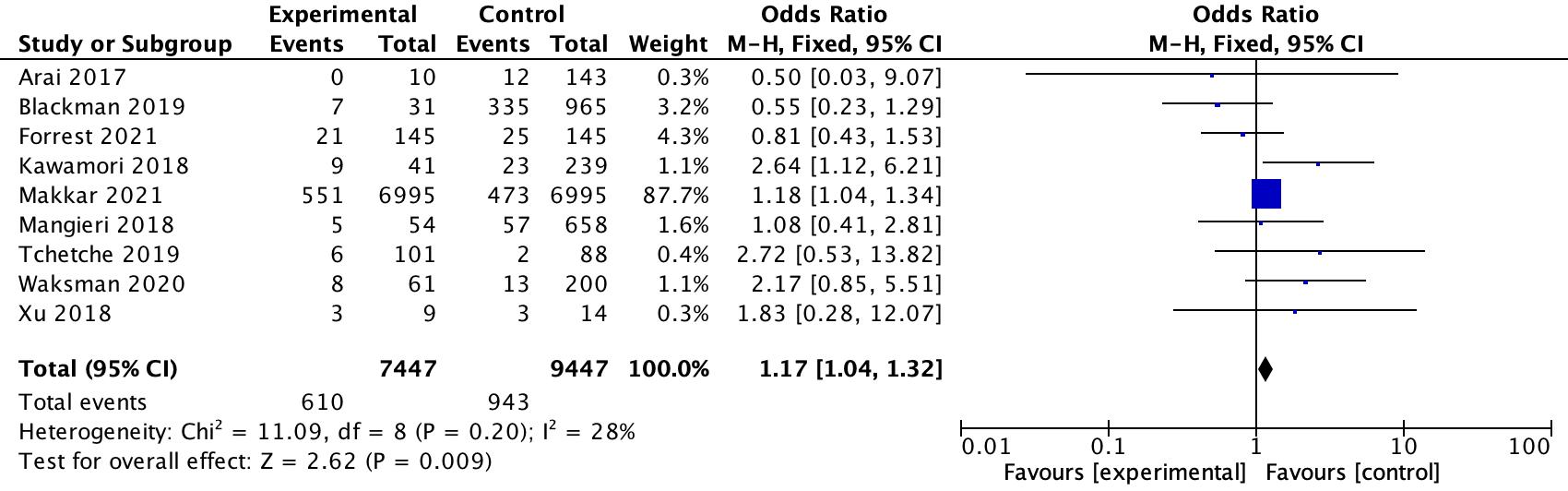


2.4 Life-threatening or major bleeding

All THVs


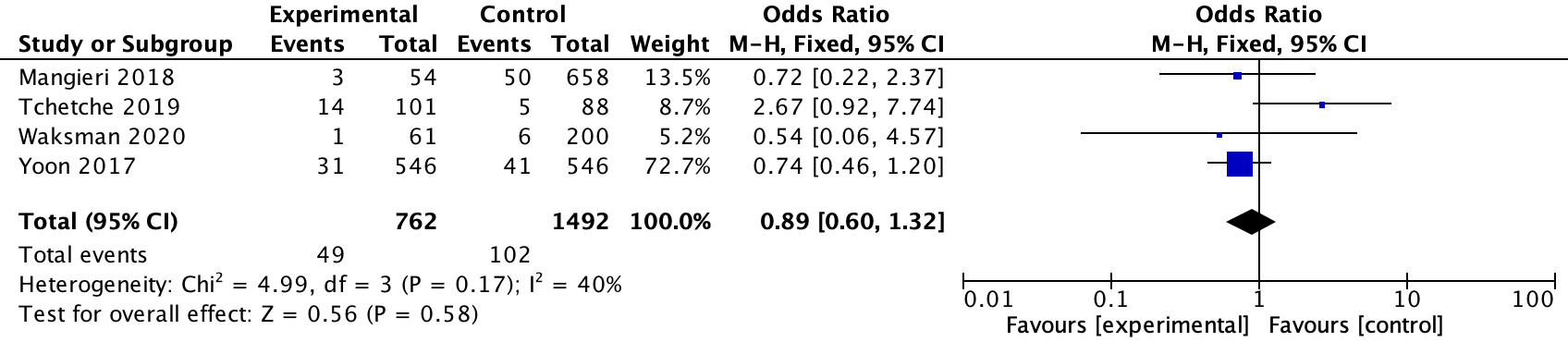


Early generation THVs

NA

New generation THVs


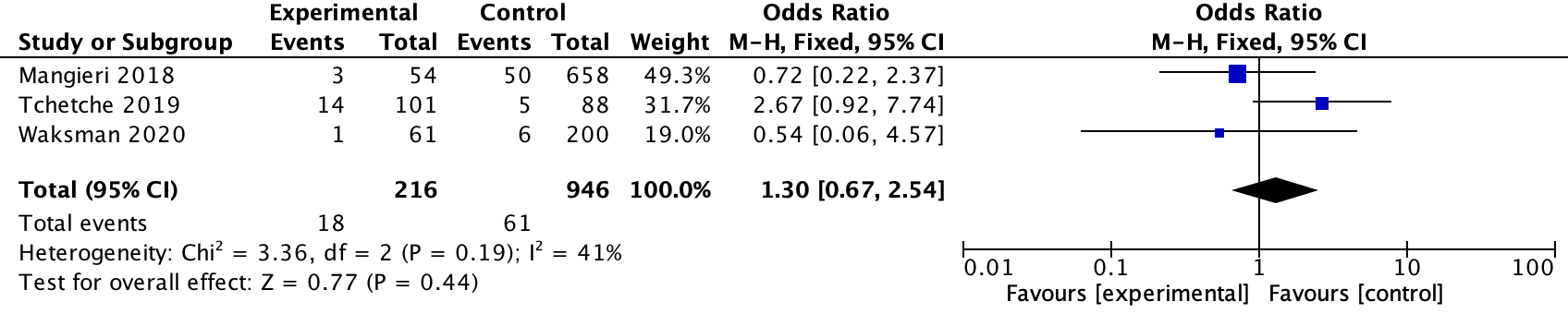


2.5 Major vascular complication

All THVs


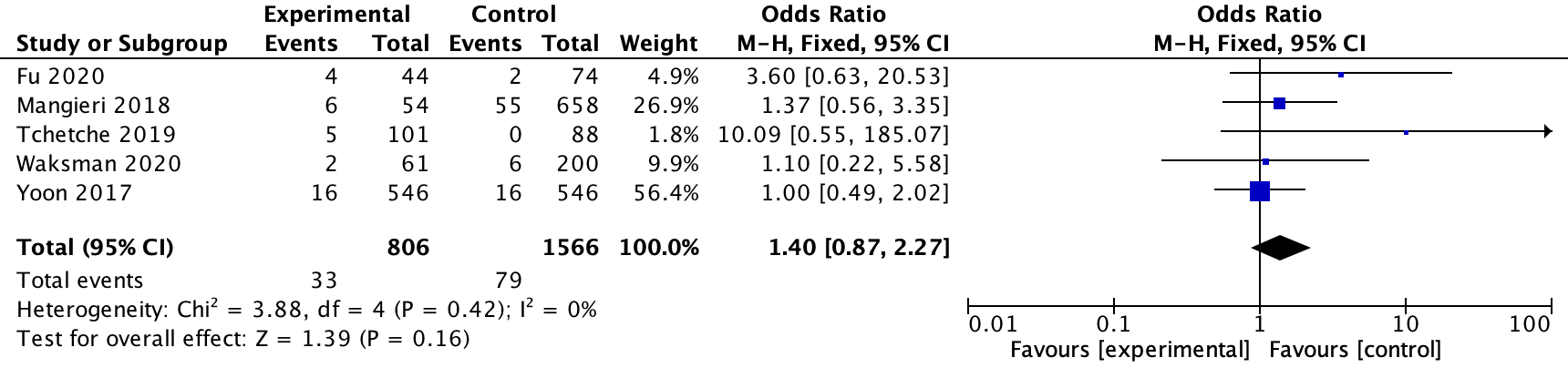


Early generation THVs


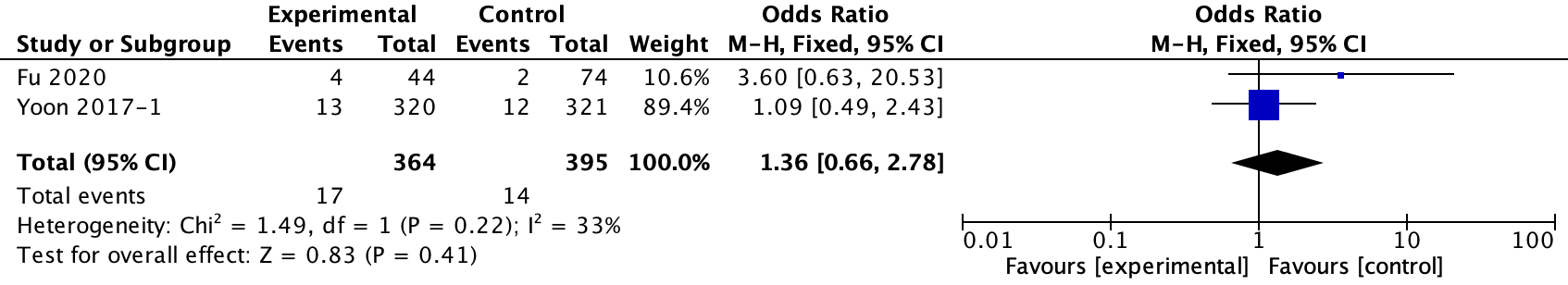


New generation THVs


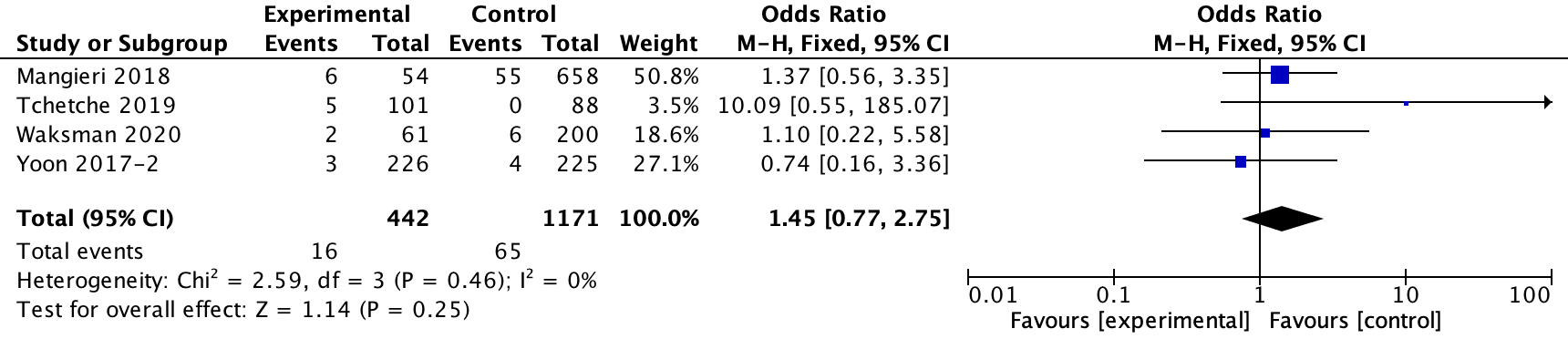


2.6 AKI

All THVs


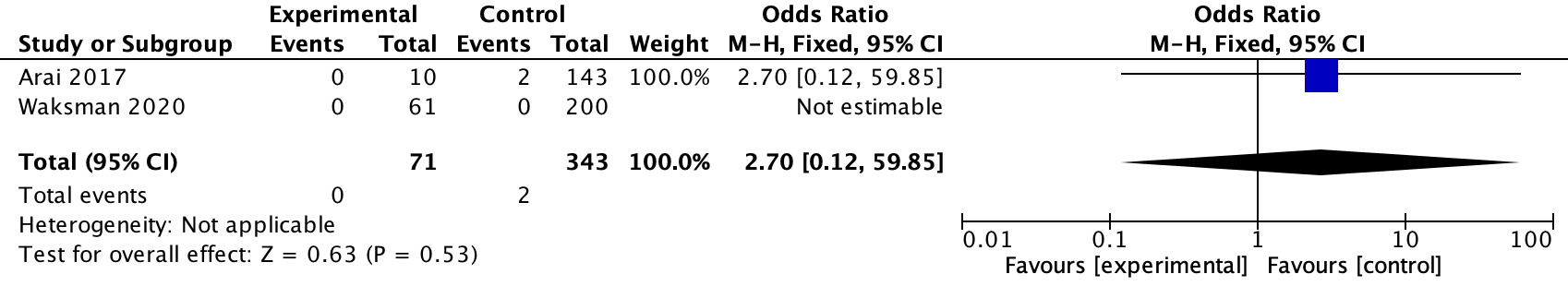


Early generation THVs

NA

New generation THVs


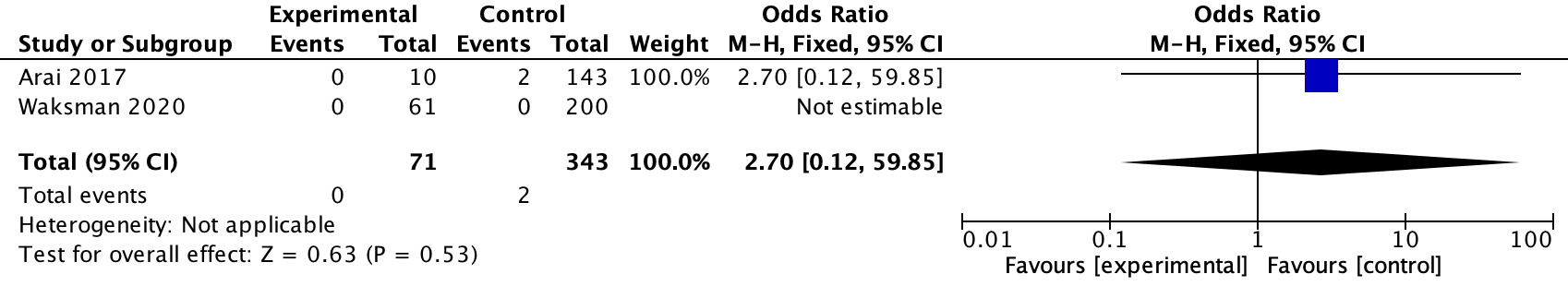


2.7 MI

All THVs


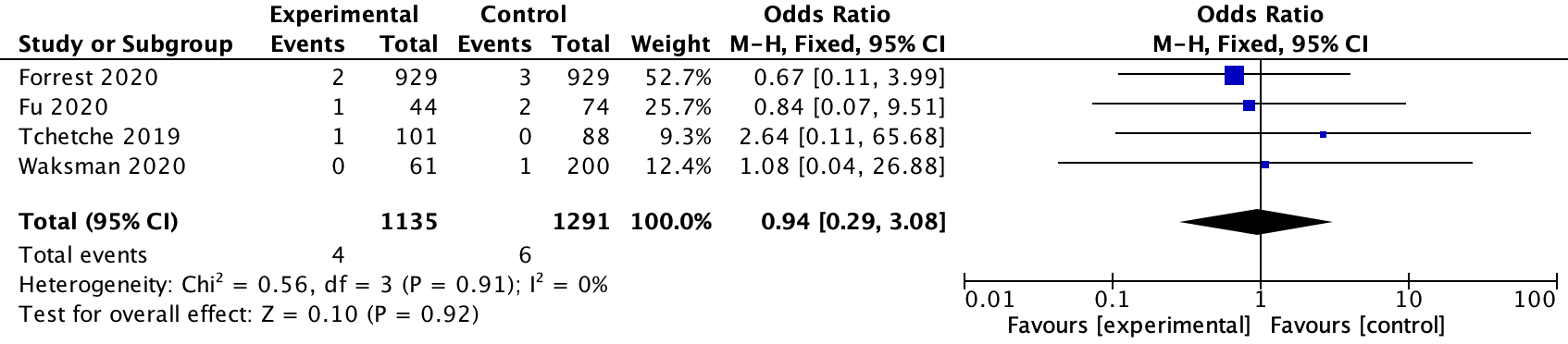


Early generation THVs


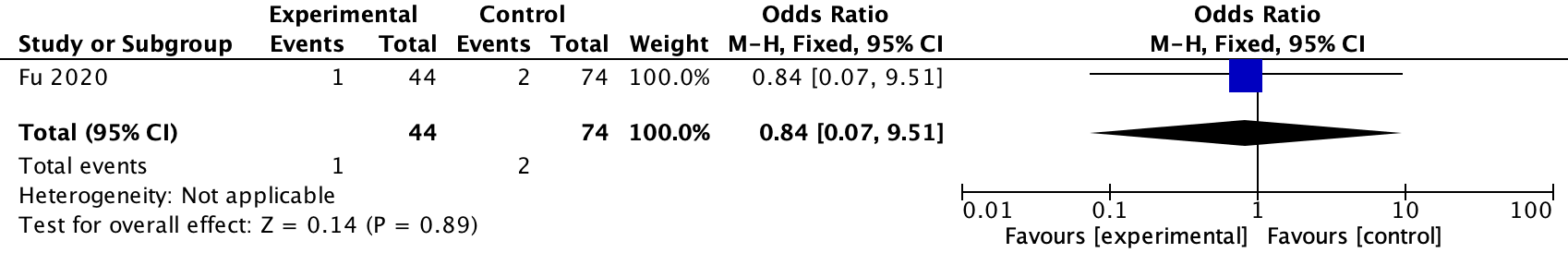


New generation THVs


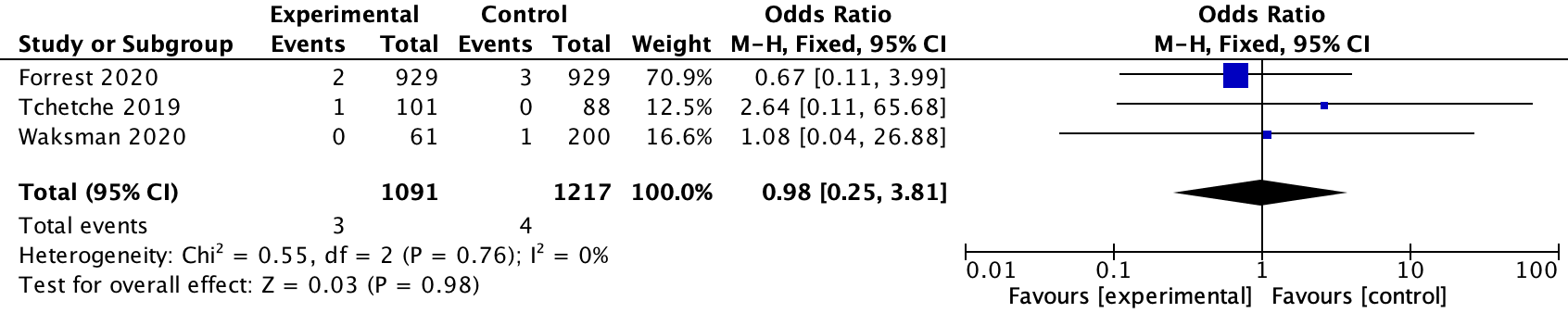


2.8 30-day mortality

All THVs


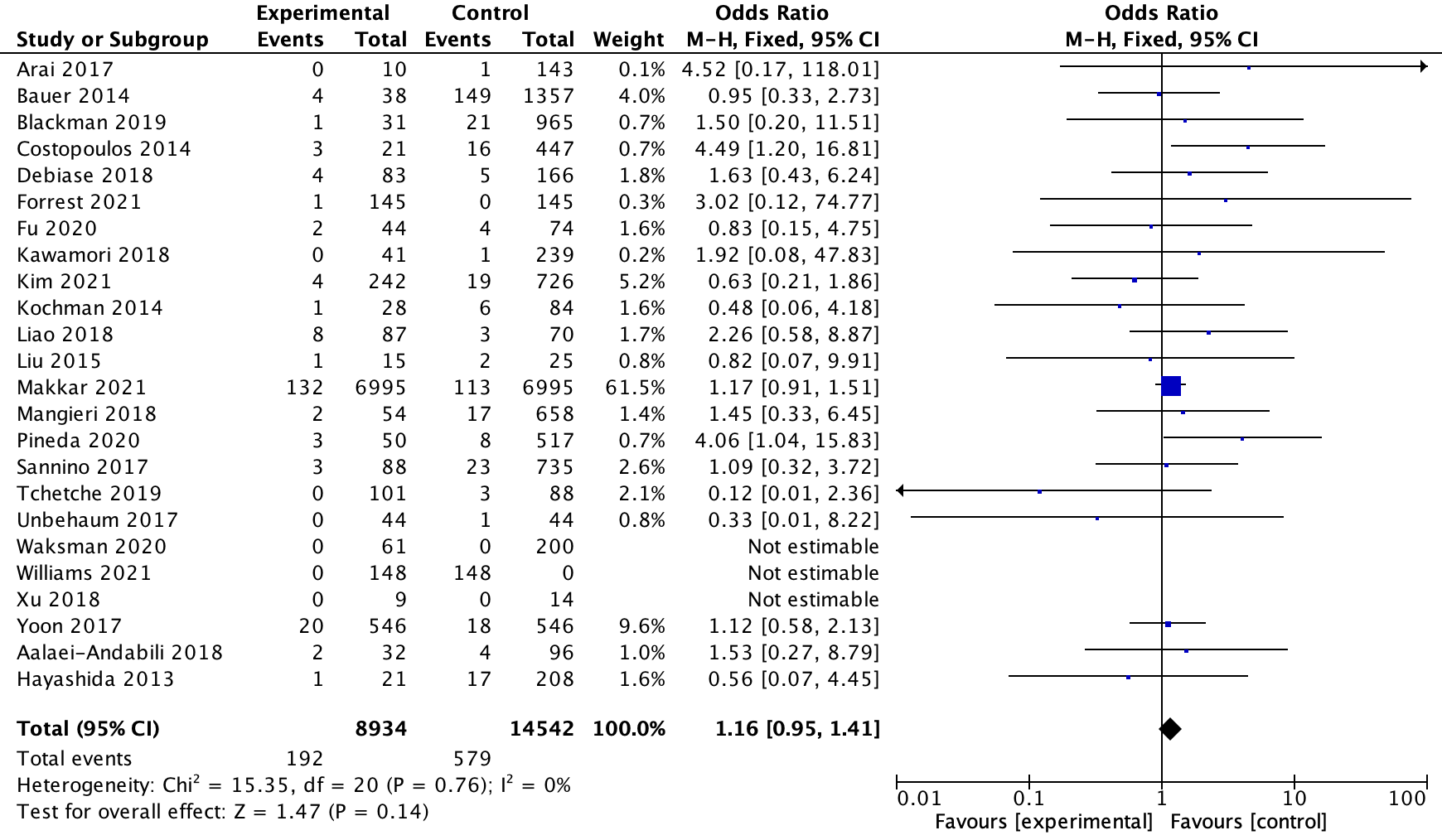


Early generation THVs


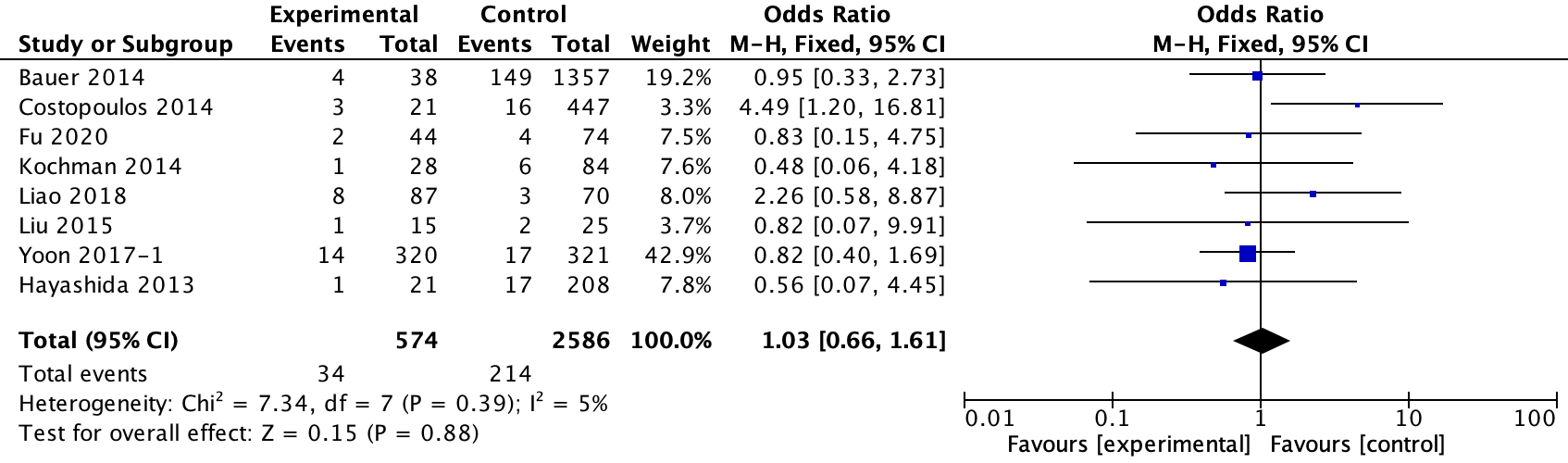


New generation THVs


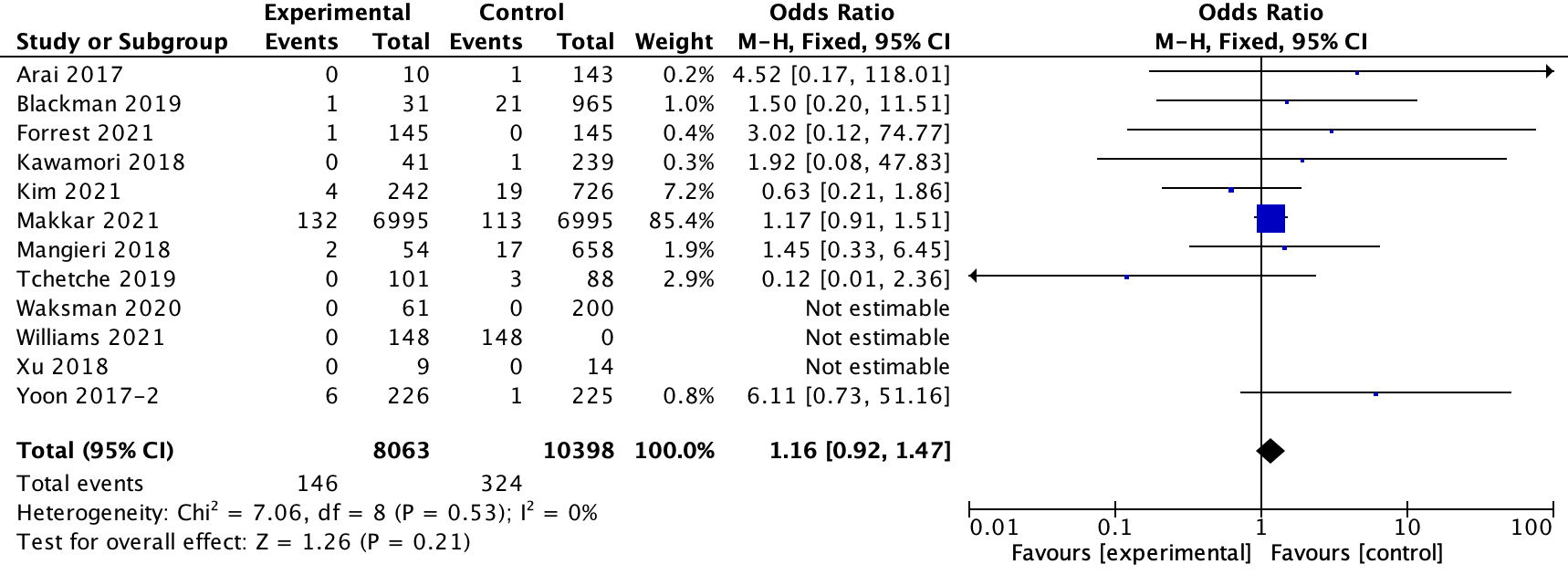


3. 1-year follow-up

3.1 Moderate or Severe PVL

All THVs


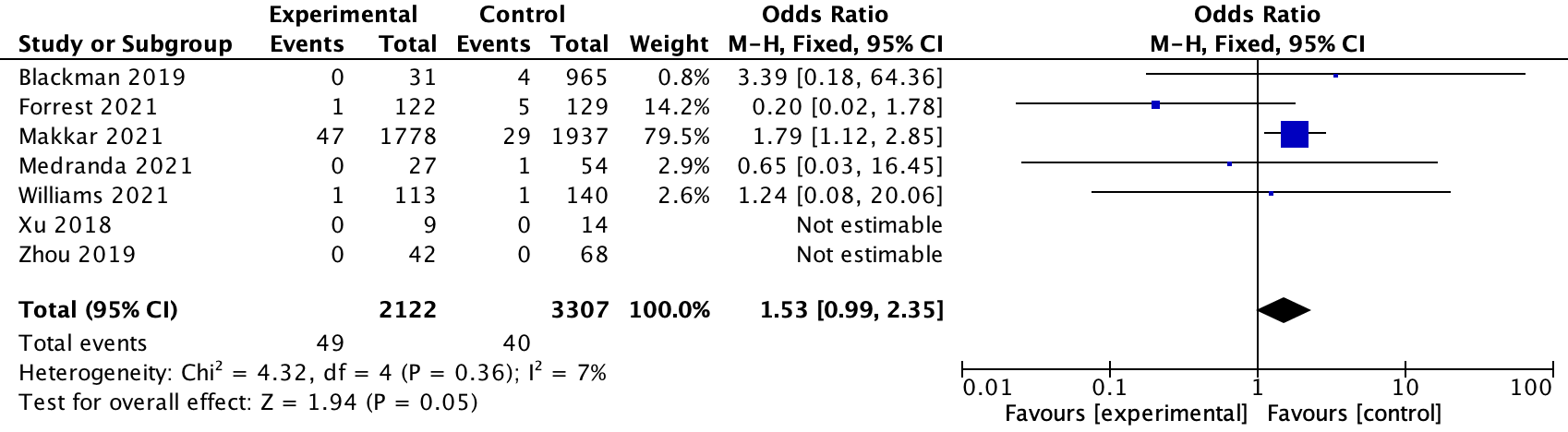


Early generation THVs


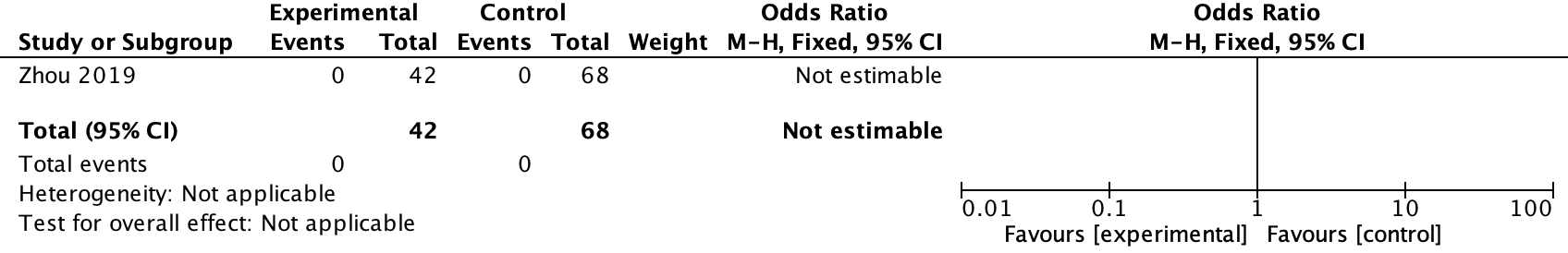


New generation THVs


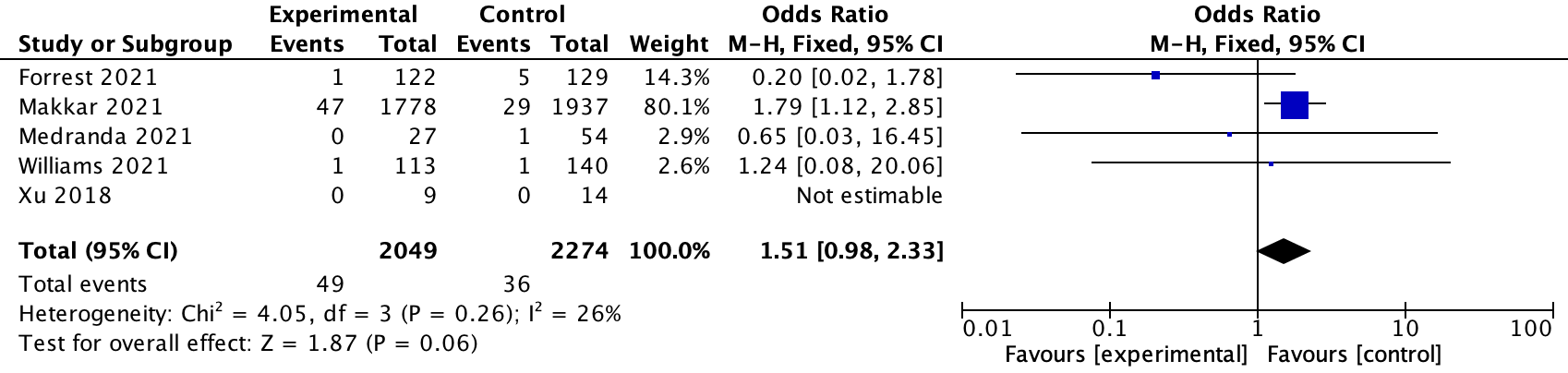


3.2 Stroke

All THVs


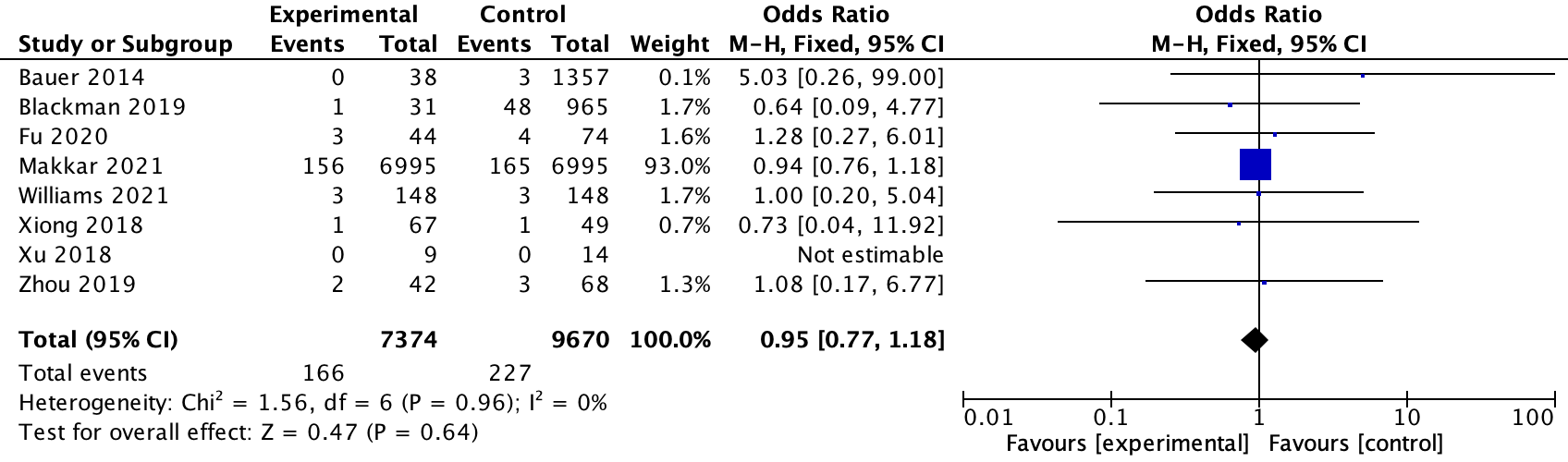


Early generation THVs


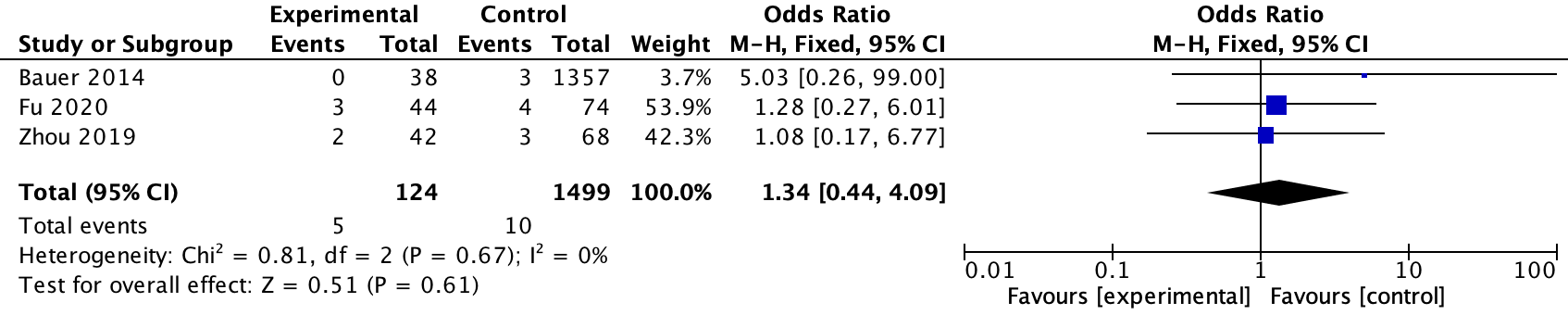


New generation THVs


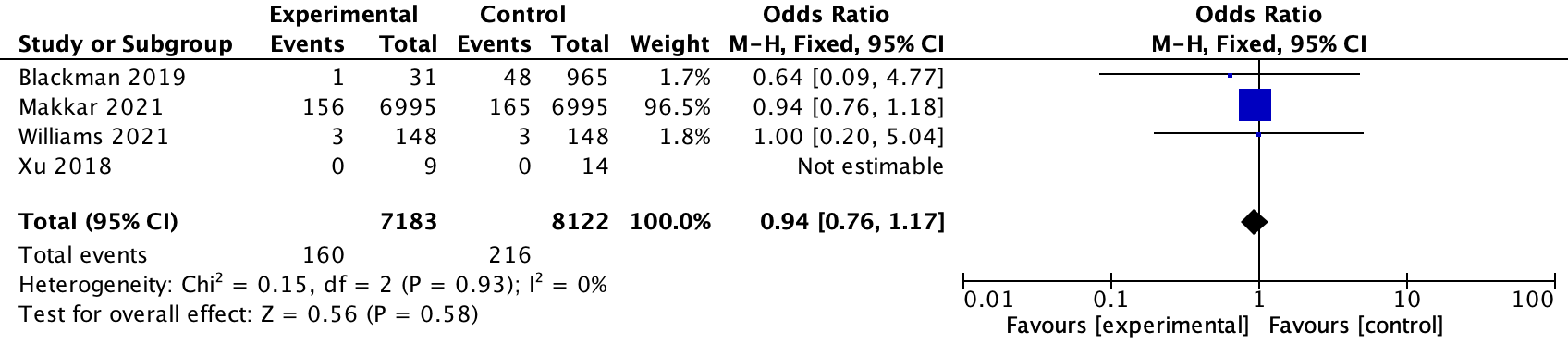


3.3 New PPI

All THVs


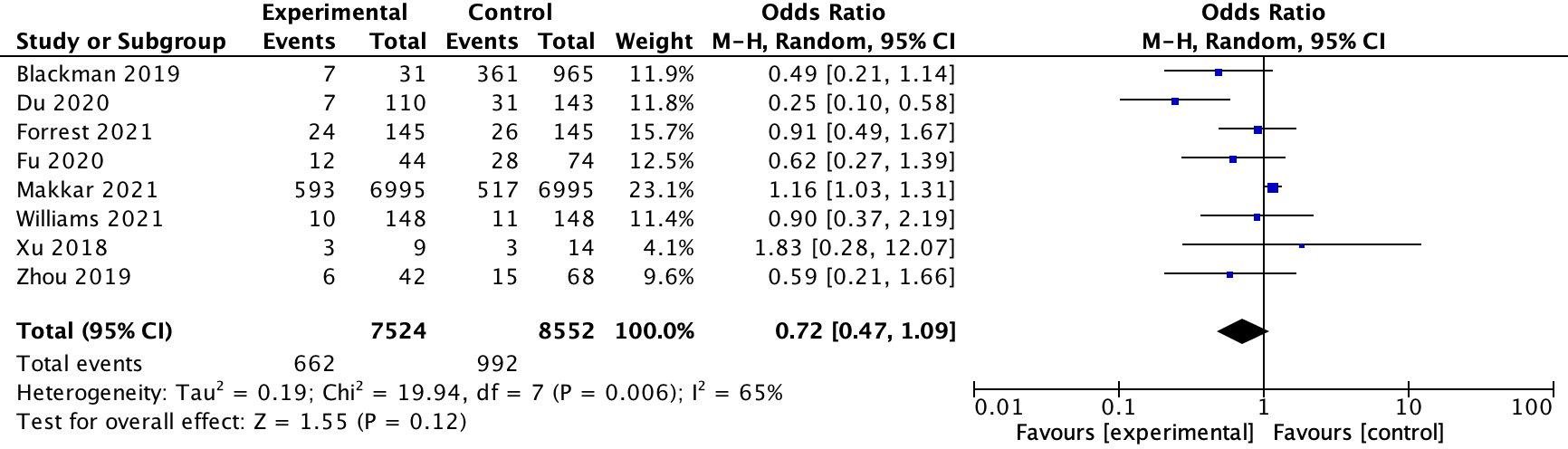


Early generation THVs


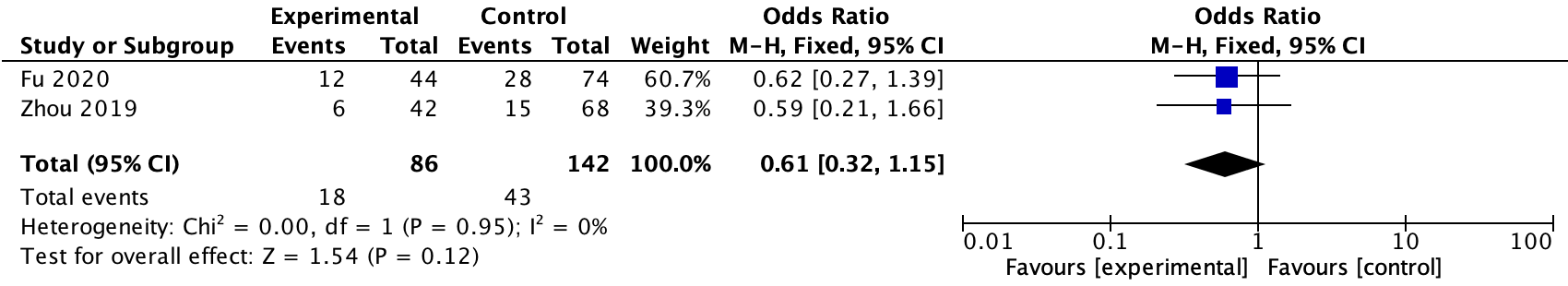


New generation THVs


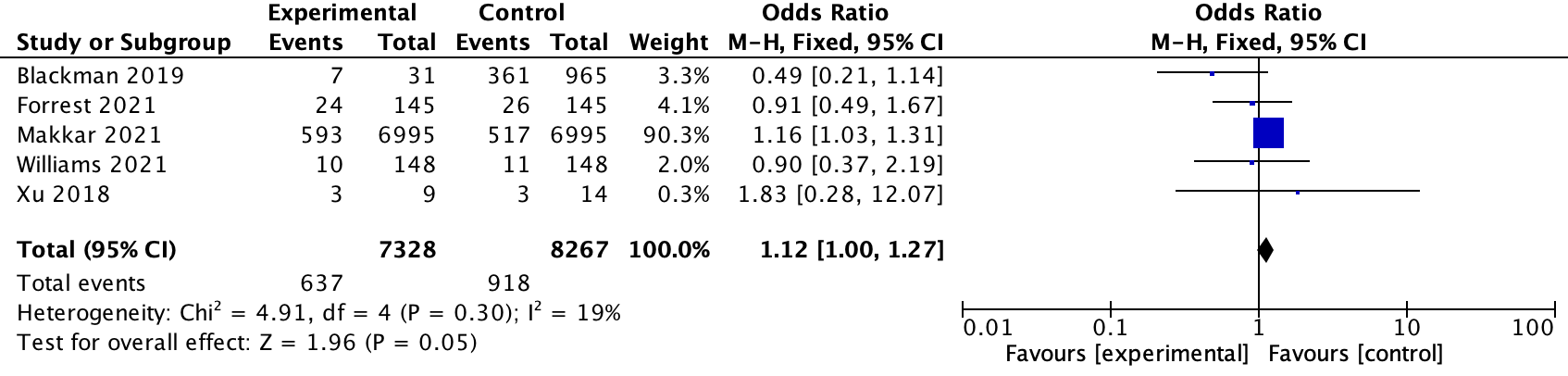


3.4 MI

All THVs


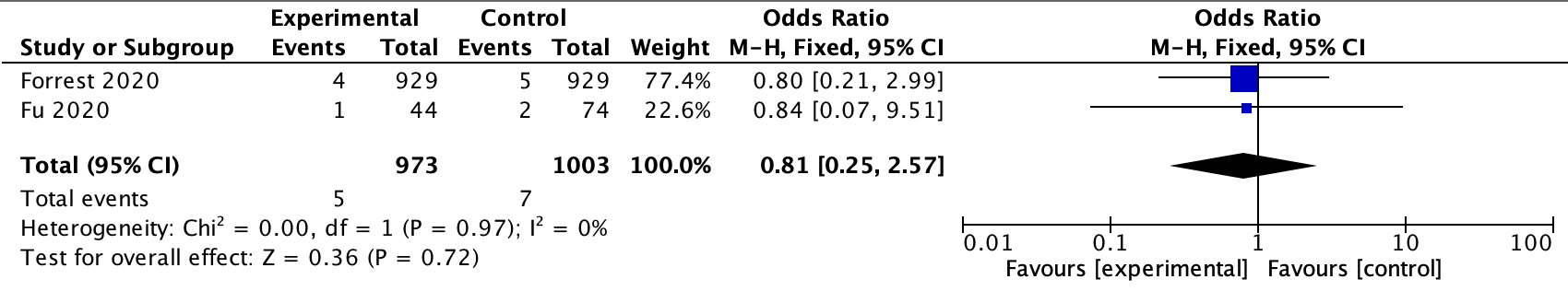


Early generation THVs


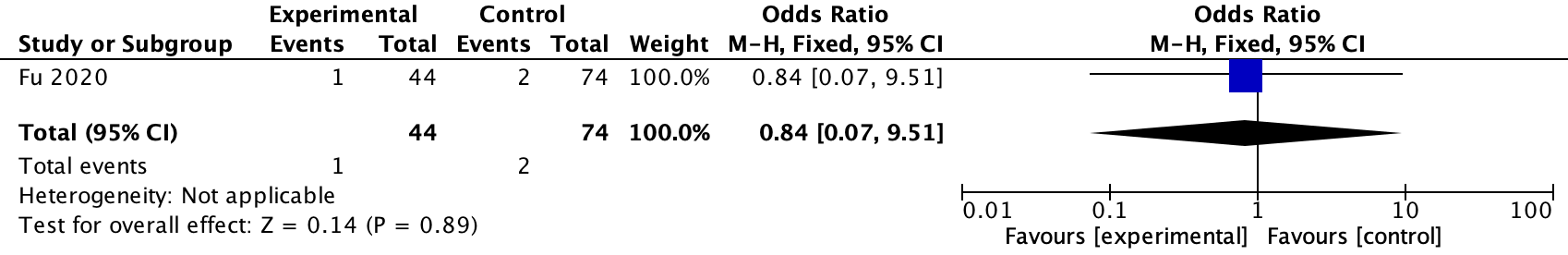


New generation THVs


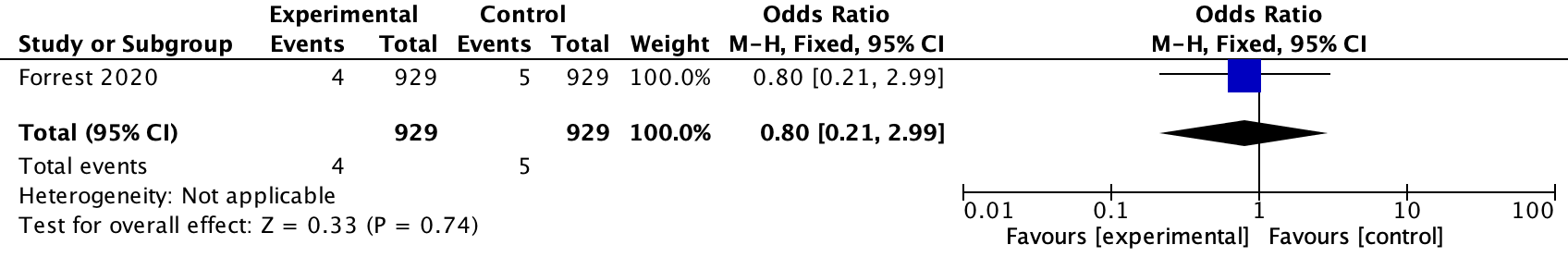


3.5 1-year mortality

All THVs


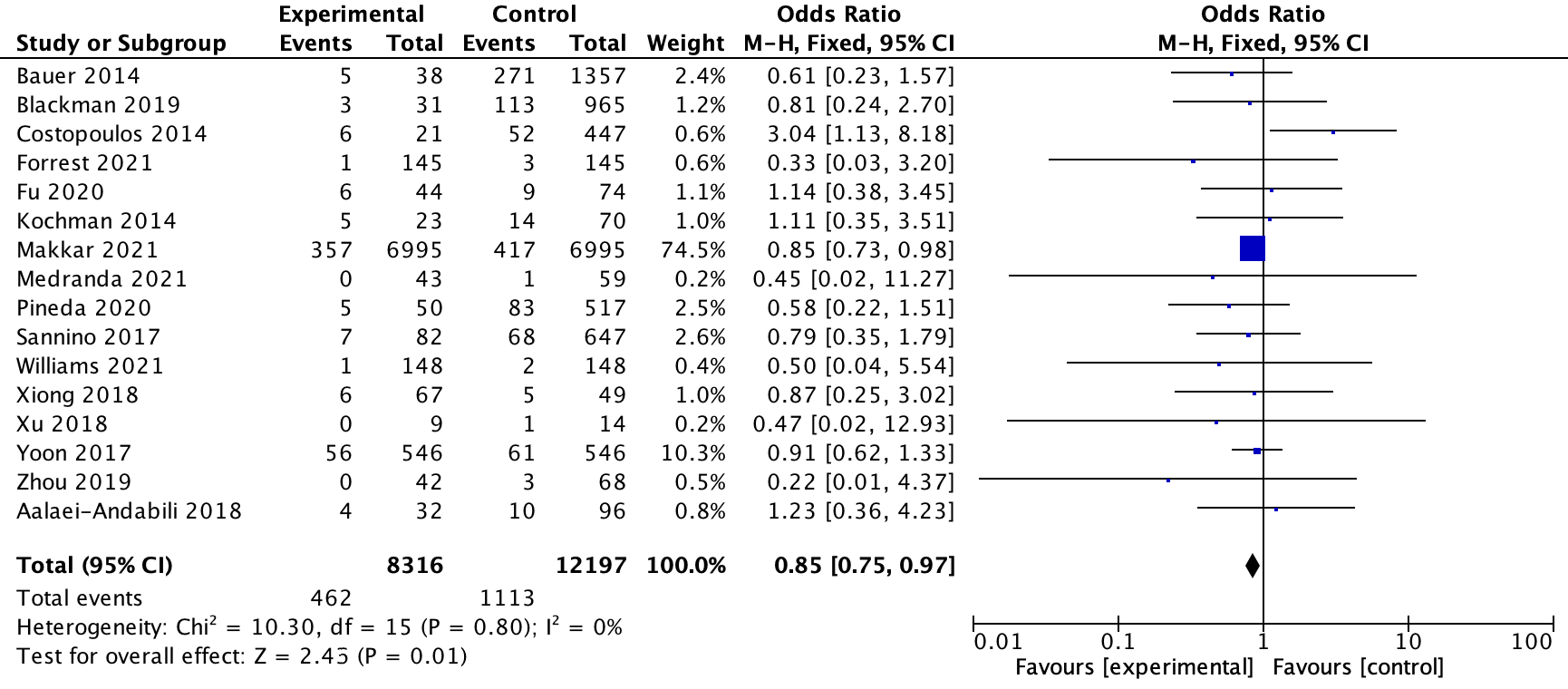


Early generation THVs


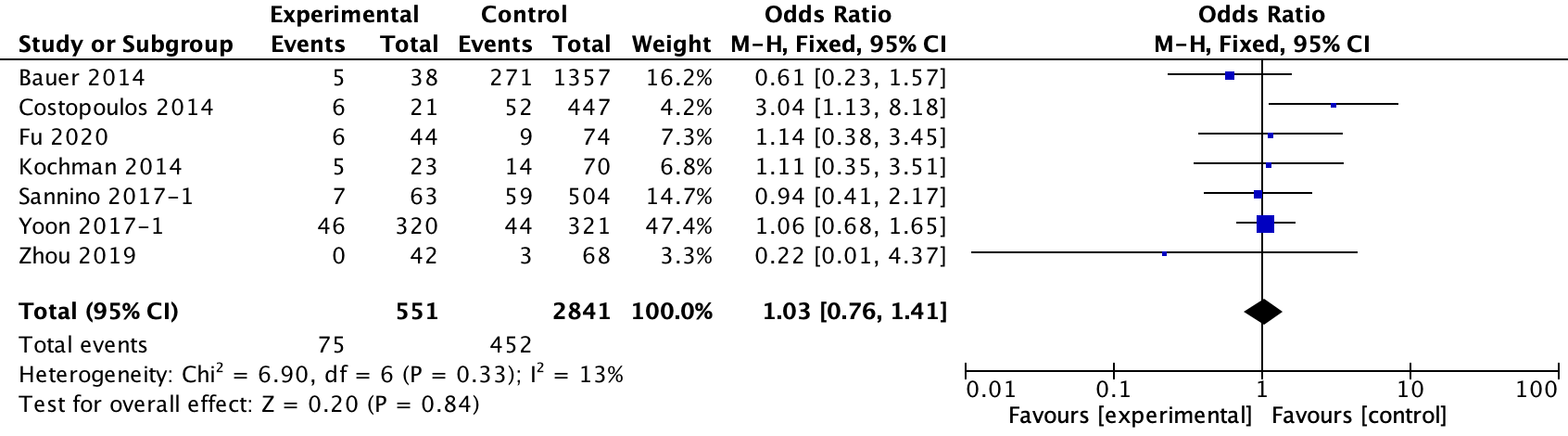


New generation THVs


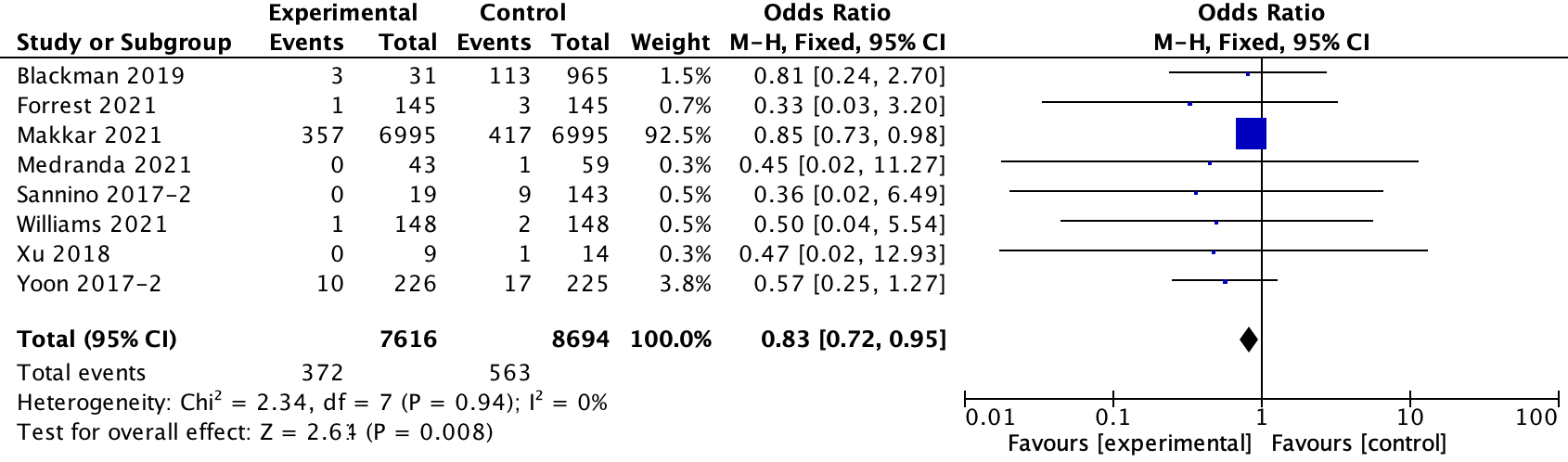


Figure 2. Original records of meta-analysis of comparisons between **BE and SE THVs in BAV patients.**

1. In-hospital outcomes

1.1 Conversion to SAVR


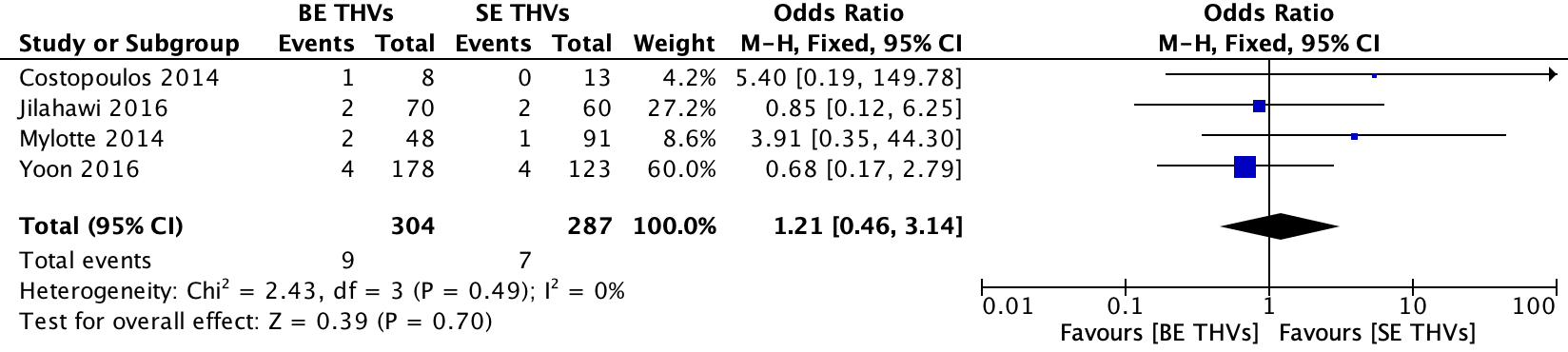


Early generation THVs


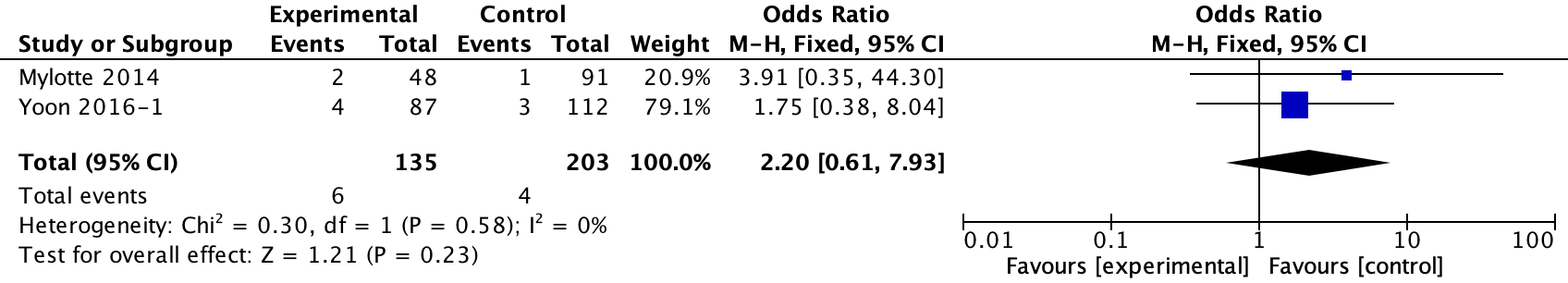


New generation THVs


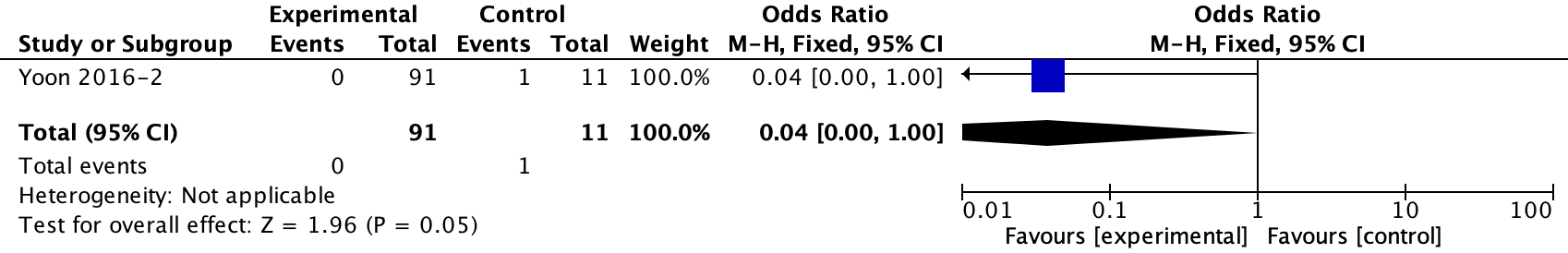
1.2 The need of a second valve


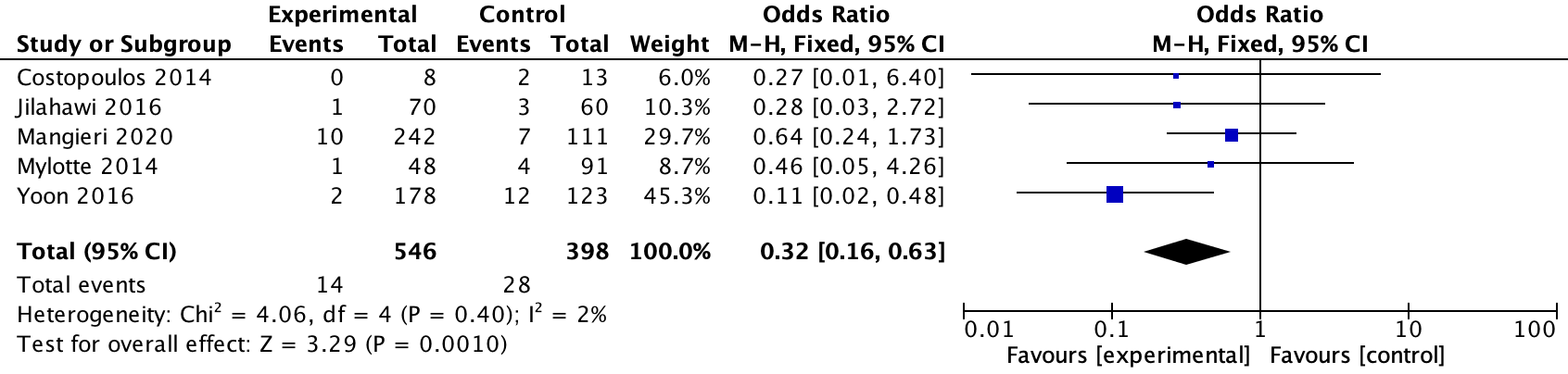


Early generation THVs


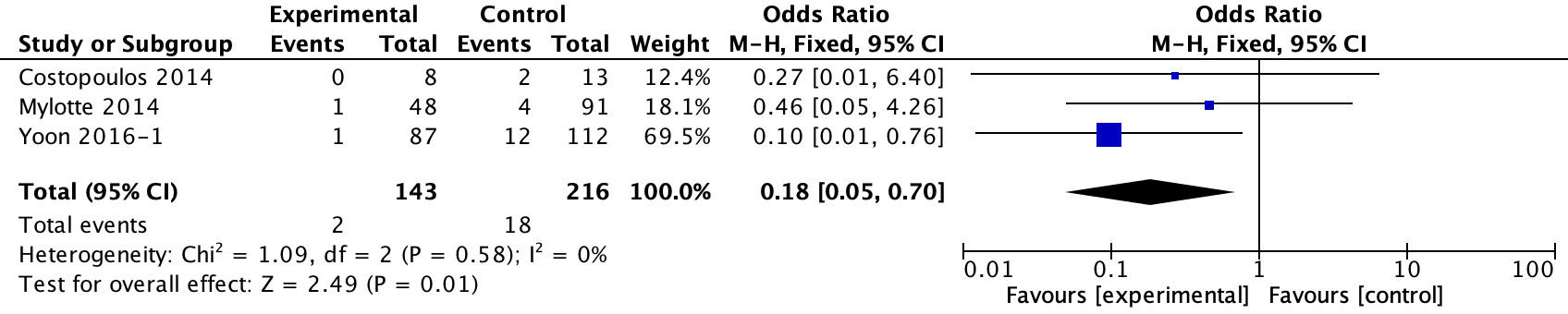


New generation THVs


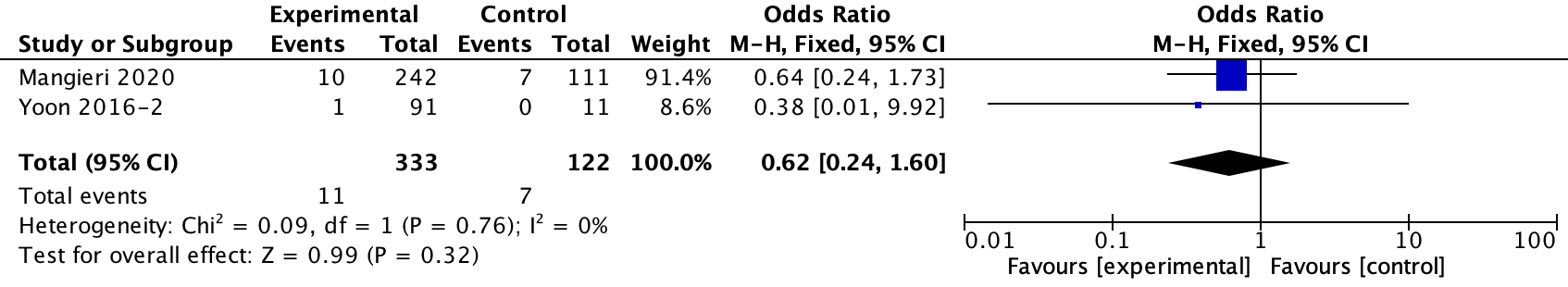


1.3 Moderate or severe PVL


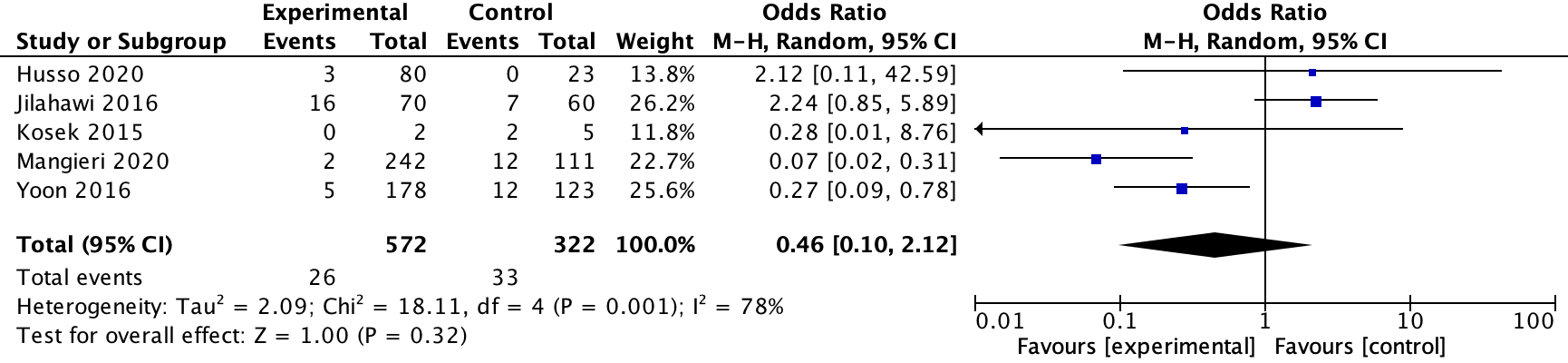


Early generation THVs


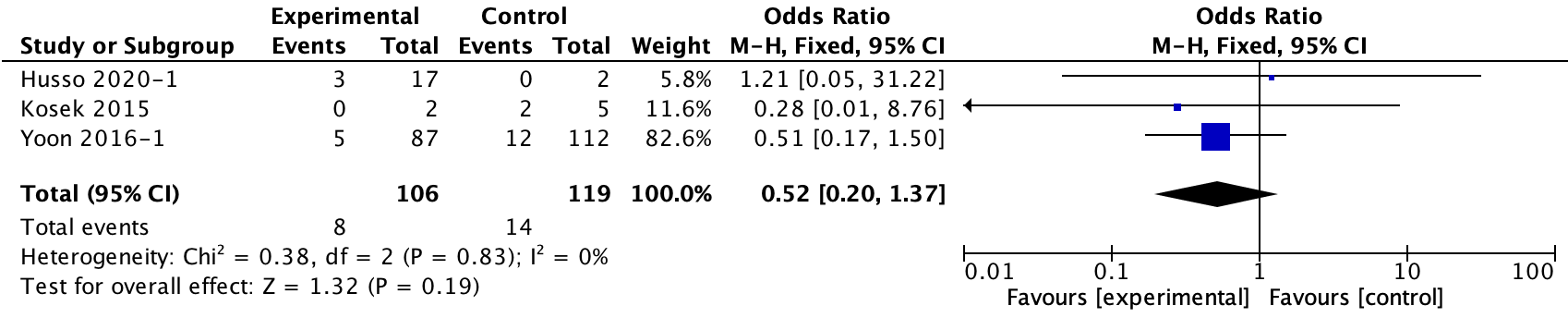


New generation THVs


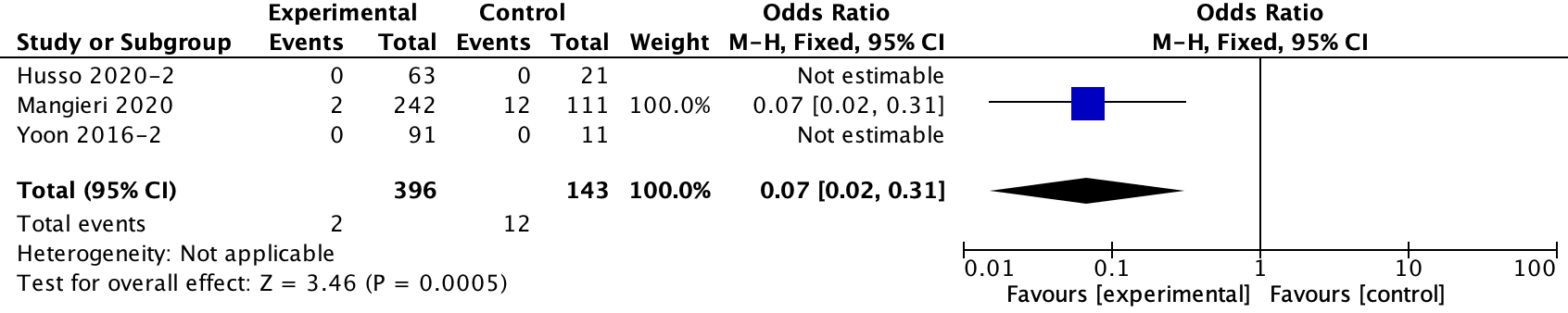


1.4 Vascular complication


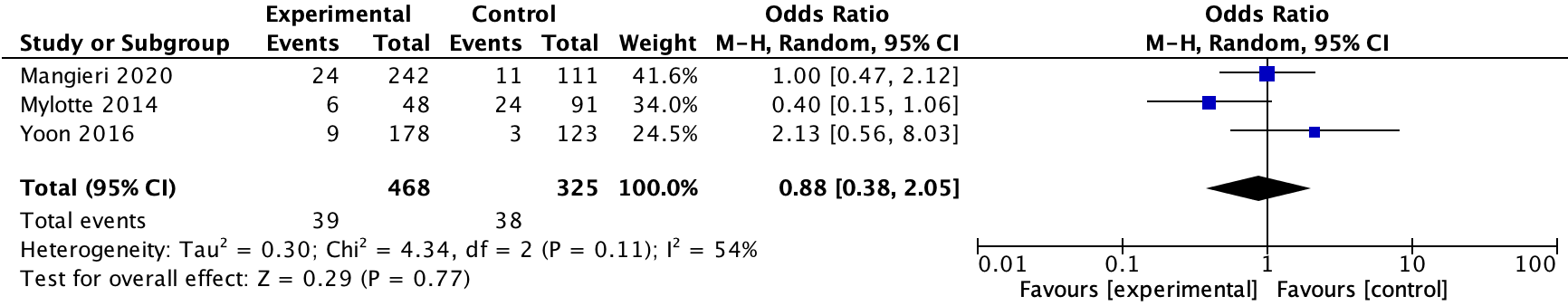


Early generation THVs


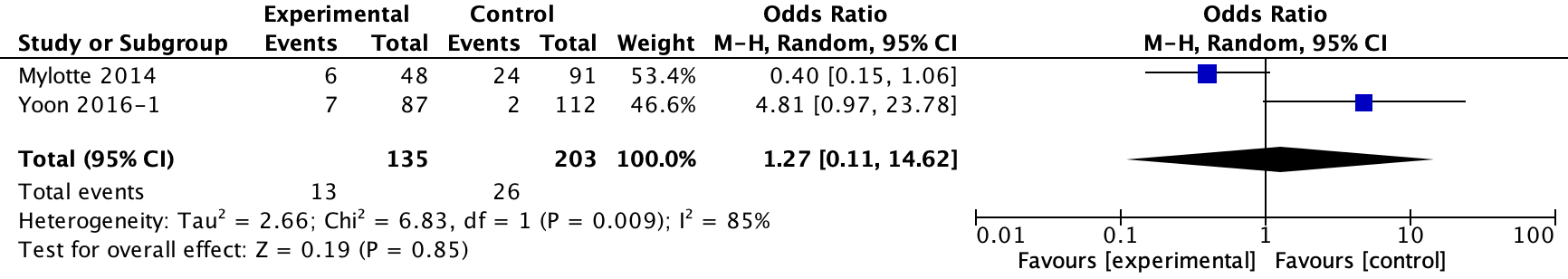


New generation THVs


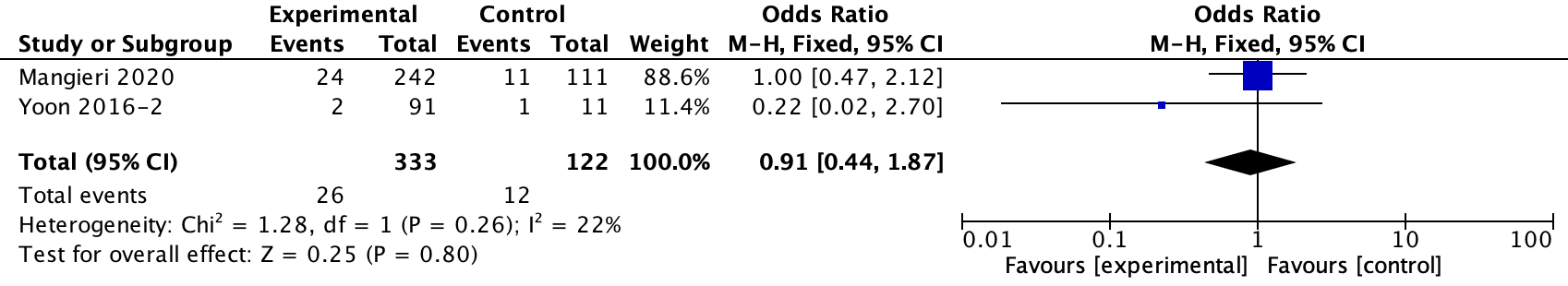


1.5 Device failure


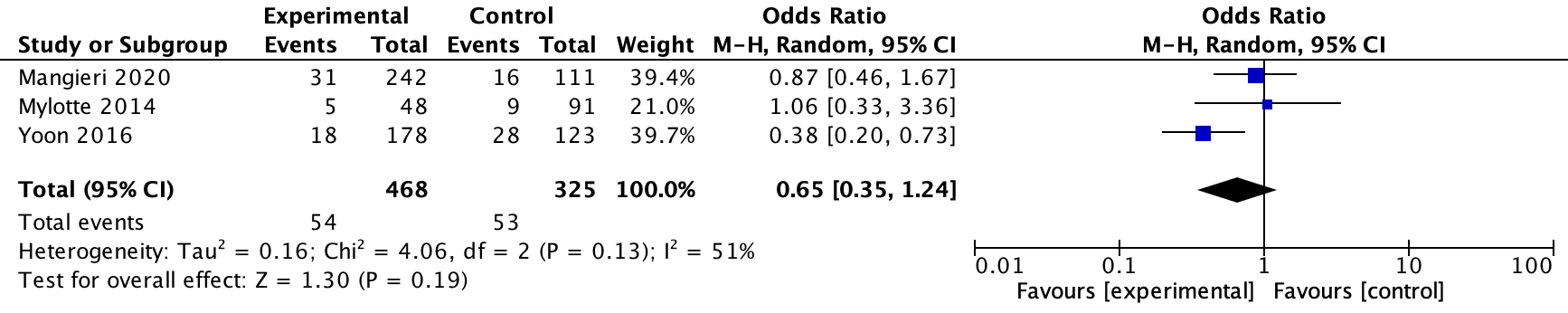


Early generation THVs


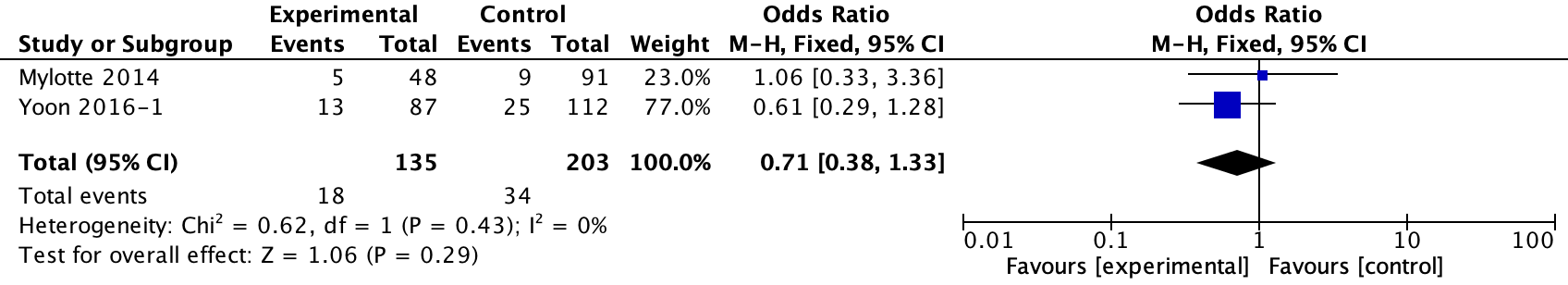


New generation THVs


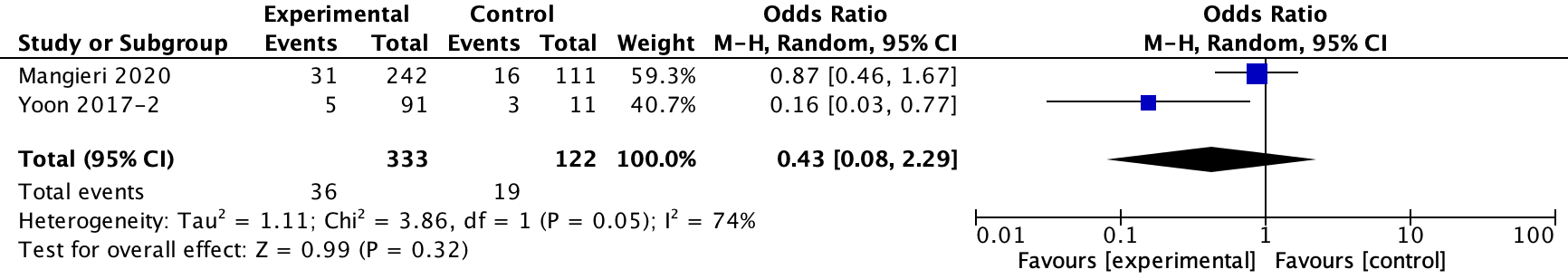


1.6 Annulus rupture


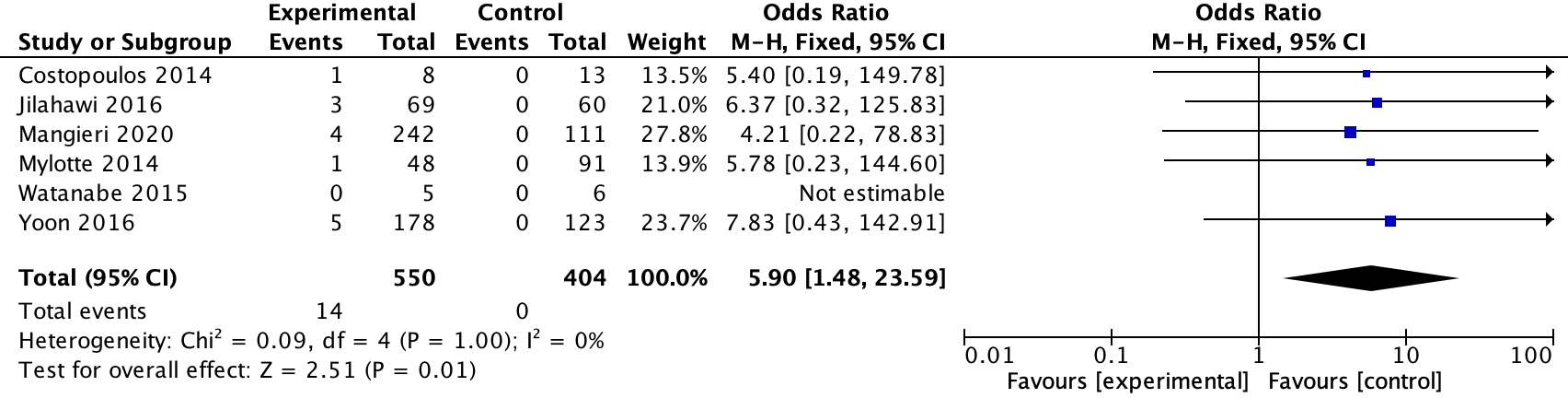


Early generation THVs


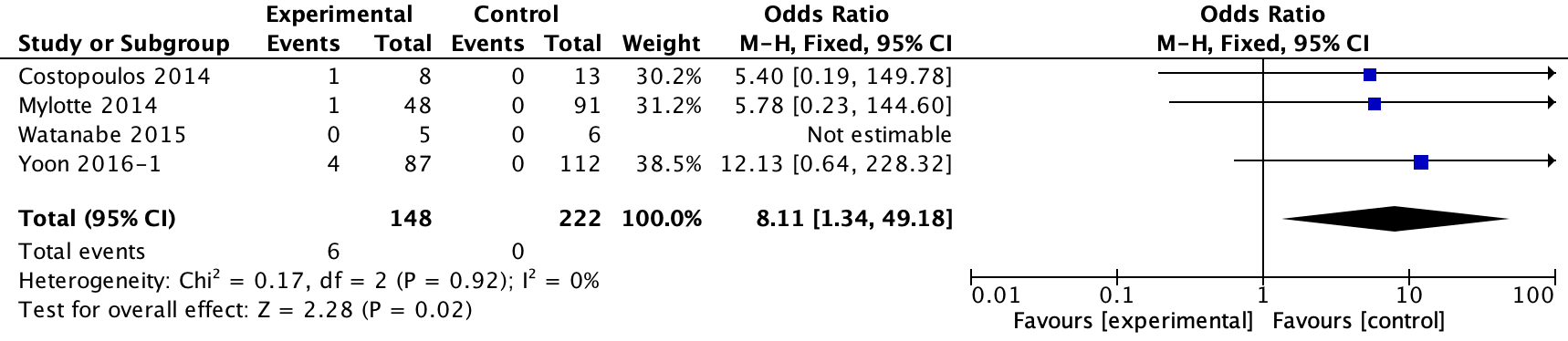


New generation THVs


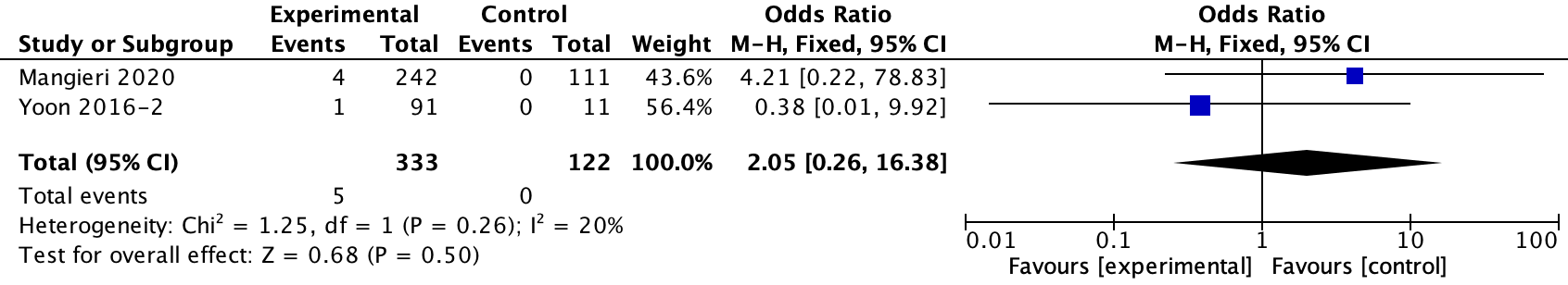


Life-threatening or major bleeding


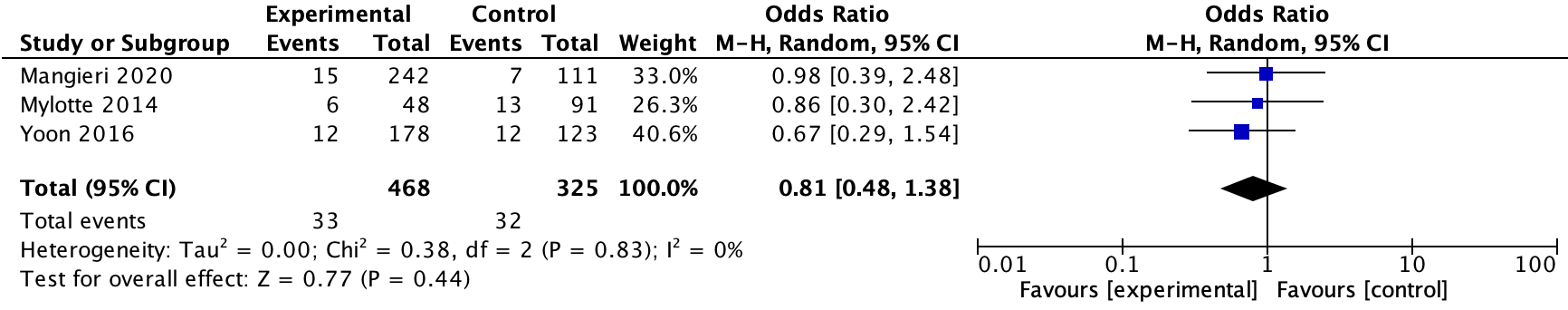


Early generation THVs


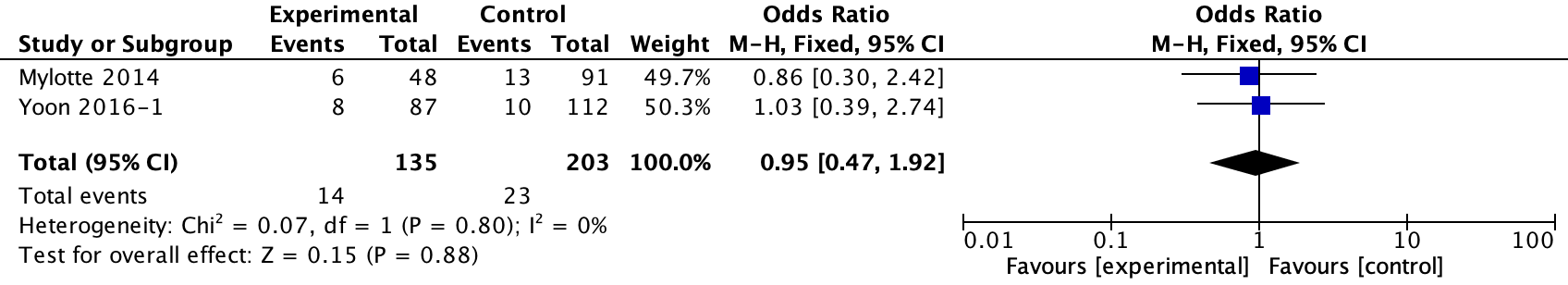


New generation THVs


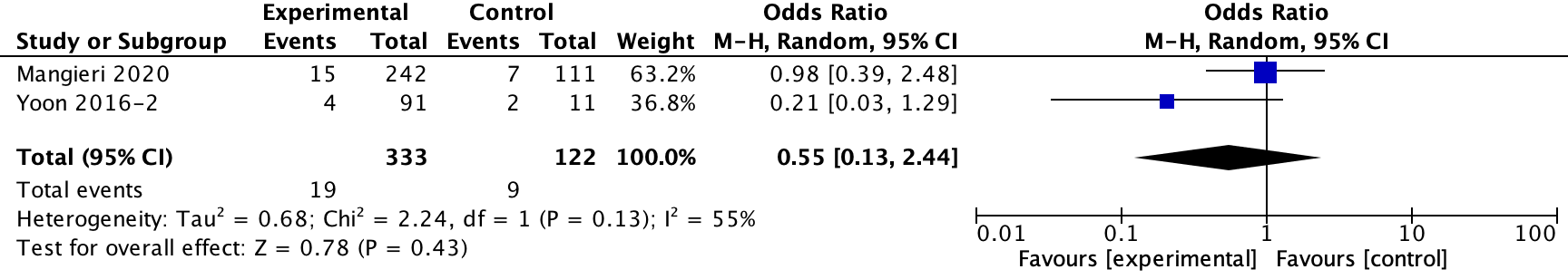


1.8 New PPI


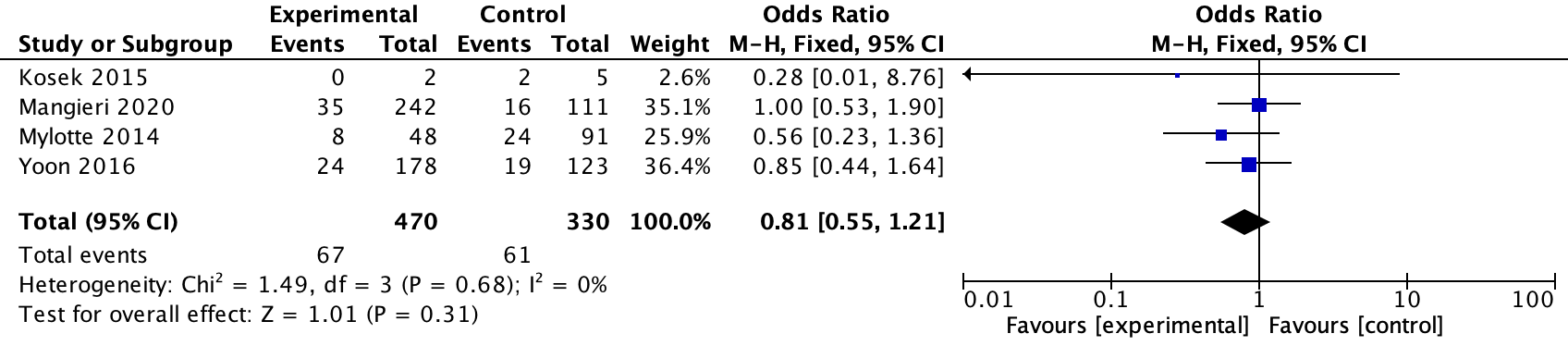


Early generation THVs

New generation THVs

1.9 Coronary obstruction

Early generation THVs

New generation THVs

1.10 In-hospital all-cause mortality

Early generation THVs

New generation THVs

2. 30-day and 1-year outcomes

2.1 30-day all-cause mortality

Early generation THVs

New generation THVs

2.2 30-day stroke

Early generation THVs

New generation THVs

2.3 30-day new PPI

Early generation THVs

New generation THVs

NA

2.4 1-year all-cause mortality

Early generation THVs

New generation THVs

Figure 3. Funnel plots of publication bias of main meta-analyses of **TAV versus BAV** **patients**.

1. In-hospital analysis
   1. Conversion to SAVR

- 1. Coronary obstruction

- 1. The need of a second valve

- 1. Moderate or severe PVL

- 1. Major vascular complication

- 1. Device failure

- 1. AKI

- 1. Annulus rupture

- 1. Life-threatening or major bleeding

- 1. MI

1.11 NO-AF

1.12 New PPI

1.13 Stroke

1.14 In-hospital all-cause mortality

1. 30-day analysis

2.1 Moderate or Severe PVL

2.2 Stroke

2.3 New PPI

2.4 Life-threatening or major bleeding

2.4 Major vascular complication

2.5 AKI

2.6 MI

2.7 30-day mortality

1. 1-year

3.1 Moderate or Severe PVL

3.2 Stroke

3.3 New PPI

3.4 MI

3.5 1-year mortality

Figure 4. Funnel plots of publication bias of main meta-analyses of **BE versus SE in BAV** patients.

1. In-hospital analysis

1.1 Conversion to SAVR

1.2 The need of a second valve

1.3 Moderate or severe PVL

1.4 Vascular complication

1.5 Device failure

1.6 Annulus rupture

1.7 Life-threatening or major bleeding

1.8 New PPI

1.9 Coronary obstruction

1.10 In-hospital all-cause mortality

1. 30-day and 1-year analysis

2.1 30-day all-cause mortality

2.2 Stroke

2.3New PPI

2.4 1-year all-cause mortality

Figure 5. Funnel plots of publication bias of meta-analysis of **THV morphology between BAV and TAV patients**.

1. THV expansion
   1. BE-inflow

1.2 BE-annulus

1.3 BE-outflow

1. THV implantation depth

2.1 Left coronary

2.2 Right coronary

2.3 Non-coronary

1. THV eccentricity index

3.1 BE-inflow

3.2 BE-annulus

3.3 BE-outflow

Supplementary Table 1. Studies included in the comparison of TAVR between **BAV** vs **TAV** patients.

|  | **Year** | **Design*** | **Center** | **Country** | **Age ± SD or [IQR] (years)** | | **Risk Score ± SD or [IQR] (%)** | | **n. of patients*** | |
| --- | --- | --- | --- | --- | --- | --- | --- | --- | --- | --- |
|  |  |  |  |  | **BAV** | **TAV** | **BAV** | **TAV** | **BAV** | **TAV** |
| Hayashida et al. | 2013 | OBS | 1 | France | 82.0 ± 7.0 | 83.2 ± 6.5 | EuroSCORE 19.9 ± 11.9 | EuroSCORE 20.1 ± 11.4 | 21 - EG | 208 - EG |
| Bauer et al. | 2014 | PSM | 22 | German | 80.7 ± 7.0 | 83.2 ± 6.5 | EuroSCORE 18 ± 10 | EuroSCORE 20 ± 13 | 38 - EG | 1357 - EG |
| Costopoulos et al. | 2014 | OBS | 1 | Italy | 76.7 ± 7.1 | 79.8 ± 7.4 | EuroSCORE 23.9 ± 12.0 | EuroSCORE 24.4 ± 17.3 | 21 - EG | 447 - EG |
| Kochman et al. | 2014 | RCT | 5 | Poland | 77.6 ± 5.5 | 79.1 ± 6.8 | EuroSCORE 19.2 ± 9.0 | EuroSCORE 18.8 ± 8.7 | 28 - EG | 84 - EG |
| Liu et al. | 2015 | OBS | 1 | China | 75.4 ± 5.7 | 75.8 ± 5.5 | STS-PROM 5.6 ± 4.1 | STS-PROM 7.5 ± 5.9 | 15 - EG | 25 - EG |
| Watanabe et al. | 2015 | OBS | 1 | France | 82.5 ± 4.6 | 84.4 ± 6.0 | EuroSCORE 21.3 11.6 | EuroSCORE 21.2 12.6 | 11 - EG | 56 - NG |
| Yoon et al. | 2017 | PSM | 33 | Europe,North America, and the Asia-Pacific region | 77.2 ± 8.2 | 77.2 ± 8.8 | STS-PROM 4.6 ± 4.6 | STS-PROM 4.3 ± 3.0 | 320 - EG 226 - NG | 226 - EG 225 - NG |
| Arai et al. | 2017 | PSM | 1 | France | 81.3 ± 5.1 | 82.5 ± 6.2 | EuroSCORE 19.0 ± 12.5 | EuroSCORE 18.1 ± 11.0 | 10 - NG | 143 - NG |
| Sannino et al. | 2017 | OBS | 2 | USA | 80.2 ± 8.4 | 81.8 ± 7.9 | STS-PROM 7.4 ± 3.9 | STS-PROM 7.6 ± 3.9 | 66 - EG 22 - NG | 550 - EG 182 - NG |
| Unbehaun et al. | 2017 | PSM` | 1 | Germany | - | - | - | - | 44 | 44 |
| Kawamori et al. | 2018 | OBS | 1 | USA | 80.0 [70.5-83.0] | 83.0 [78.0-87.0] | NA | NA | 41 - NG | 239 - NG |
| Xu et al. | 2018 | OBS | 1 | China | 71.2 ± 4.8 | 76.9 ± 5.7 | STS-PROM 5.5 ± 3.0 | STS-PROM 9.8 ± 7.8 | 9 - NG | 14 - NG |
| Liao et al. | 2018 | OBS | 1 | China | 73.4 ± 6.4 | 74.3 ± 7.0 | STS-PROM 7.9 ± 4.0 | STS-PROM 8.6 ± 4.4 | 87 - EG | 70 - EG |
| Mangieri et al. | 2018 | OBS | 7 | Italy | 80.0 ± 5.3 | 82.1 ± 4.3 | STS-PROM 4.7 ± 2.7 | STS-PROM 5.1 ± 3.6 | 54 - NG | 658 - NG |
| Debiase et al. | 2018 | PSM | 1 | France | 81.4 ± 7.6 | 82.9 ± 5.7 | STS-PROM 5.1 ± 3.3 | STS-PROM 5.1 ± 2.9 | 83 | 166 |
| Aalaei-Andabili et al. | 2018 | OBS | 1 | USA | 68.6 ± 11.1 | 74.0 ± 10.8 | STS-PROM 6.0 ±3.4 | STS-PROM 6.1 ±3.8 | 32 | 96 |
| Nagaraja et al. | 2018 | PSM | NIS database | USA | 68.01 ± 13.42 | 68.15 ± 12.71 | NA | NA | 359 | 359 |
| Xiong et al. | 2018 | OBS | 1 | China | 74.0 [68.0, 77.0] | 75.0 [68.0, 79.0] | STS-PROM 6.5 [4.4, 9.3] | STS-PROM 8.3 [5.2, 9.5] | 67 | 49 |
| Zhou et al. | 2019 | OBS | 11 | China | 76.4 ± 4.6 | 78.6 ± 4.8 | STS-PROM 7.4 ±3.9 | STS-PROM 9.7 ±6.3 | 42 - EG | 68 - EG |
| Blackman et al. | 2019 | OBS | 41 | Europe, New Zealand, and  Latin America | 76.4 ± 7.9 | 80.9 ± 6.4 | STS-PROM 6.0 ±10.2 | STS-PROM 5.9 ±6.5 | 31 - NG | 965 - NG |
| Tchetche et al. | 2019 | OBS | 8 | Europe and Israel | 78.2 ± 10.1 | 83.1 ± 5.7 | STS-PROM 11.3 ±8.5 | STS-PROM 7.6 ± 4.4 | 101 - NG | 88 - NG |
| Elbadawi et al. | 2019 | PSM | NIS database | USA | 68.4 ± 11.9 | 68.8 ± 12.2 | NA | NA | 1035 | 1035 |
| Forrest et al. | 2020 | PSM | STS/ACC TVT Registry | USA | 73.0 ± 10.3 | 72.6 ± 10.8 | STS-PROM 5.3 ± 4.2 | STS-PROM 5.2 ± 4.1 | 929 - NG | 929 - NG |
| Waksman et al. | 2020 | OBS | 6 | USA | 68.6 ± 7.4 | 73.6 ± 6.1 | STS-PROM 1.5 ± 0.6 | STS-PROM 1.8 ± 0.5 | 61 - NG | 200 - NG |
| Fu et al. | 2020 | OBS | 2 | China | 73.6 ± 6.3 | 75.7 ± 7.3 | STS-PROM 6.7 ± 3.0 | STS-PROM 7.6 ±5.6 | 44 - EG | 74 - EG |
| Halim et al. | 2020 | OBS | STS/ACC TVT Registry | USA | 73.0 [65.0-81.0] | 82.0 [76.0-87.0] | STS-PROM 3.6 [2.2-5.8] | STS-PROM 5.3 [3.5-8.3] | 3705 - NG | 104818 - NG |
| Pineda | 2020 | OBS | 1 | USA | 70 [64-74] | 81 [75-86] | STS-PROM 4.6 [3.0-7.7] | STS-PROM 6.7 [4.4-9.9] | 50 | 517 |
| Ahuja et al. | 2020 | PSM | Nationwide Readmission Database | USA | NA | NA | NA | NA | 394 | 1182 |
| Du et al. | 2020 | OBS | 1 | China | NA | NA | NA | NA | 110 | 143 |
| Hamdan et al. | 2020 | PSM | 5 | Israel | 77.0 ± 8.8 | 76.8 ± 9.4 | NA | NA | 67 | 67 |
| Kim et al. | 2021 | PSM | 1 | Germany | 80.0 [75.2-83.5] | 80.4 [76.7-83.5] | EuroSCORE 16.6 [10.2-24.4] | EuroSCORE 17.4 [10.7-25.5] | 242 - NG | 726 - NG |
| Williams et al. | 2021 | PSM | 85 | USA, New Zealand, Japan, Australia, Canada | 71 | 72 | 1.4 | 1.5 | 148 - NG | 148 - NG |
| Forrest et al. | 2021 | PSM | 25 | USA | NA | NA | NA | NA | 145 - NG | 145 - NG |
| Medranda et al. | 2021 | OBS | 6 | USA | 68.4 ± 7.8 | 76.0 ± 5.7 | STS-PROM 1.5 ± 0.6 | STS-PROM 1.9 ± 0.5 | 47 - NG | 60 - NG |
| Makkar et al. | 2021 | PSM | STS/ACC TVT Registry | USA | 71.9 ± 10.0 | 71.9 ± 10.5 | STS-PROM 4.0 ± 3.7 | STS-PROM 4.0 ± 3.5 | 6995 - NG | 6995 - NG |

*EG represents prosthesis of Early Generation, NG represents prosthesis of New Generation.

Abbreviations: OBS = observational study, PSM = propensity score matching, STS-PROM, Society of Thoracic Surgeons Predicted Risk of Mortality; EuroSCORE, European System for Cardiac Operative Risk Evaluation.

Supplementary Table 2. Assessment of risk bias with ROBINS-I tool in the studies comparing **BAV with TAV** in TAVR

| Study | Bias due to confounding | Bias in selection of participants into the study | Bias in classification of interventions | Bias due to deviations from intended interventions | Bias due to missing data | Bias in measurement of outcomes | Bias in selection of the reported result | Overall bias |
| --- | --- | --- | --- | --- | --- | --- | --- | --- |
| Hayashida et al. | Low | Low | Low | Low | Low | Low | Moderate | Low |
| Bauer et al. | Low | Low | Low | Low | Moderate | Low | Moderate | Low |
| Costopoulos et al. | Low | Low | Low | Low | Low | Low | Moderate | Low |
| Kochman et al. | Low | Low | Low | Low | Low | Low | Moderate | Low |
| Liu et al. | Low | Low | Low | Low | Low | Moderate | Moderate | Low |
| Watanabe et al. | Low | Low | Low | Low | Low | Moderate | Low | Low |
| Yoon et al. | Low | Low | Low | Low | Moderate | Moderate | Moderate | Moderate |
| Arai et al. | Low | Low | Low | Low | Low | Moderate | Low | Low |
| Sannino et al. | Low | Low | Low | Low | Low | Low | Moderate | Low |
| Unbehaun et al. | Low | Low | Low | Low | Low | Moderate | Low | Low |
| Kawamori et al. | Low | Low | Low | Low | Low | Moderate | Low | Low |
| Xu et al. | Low | Moderate | Low | Low | Moderate | Low | Low | Low |
| Liao et al. | Low | Low | Low | Low | Moderate | Low | Moderate | Low |
| Mangieri et al. | Low | Moderate | Low | Low | Low | Moderate | Low | Low |
| Debiase et al. | Low | Low | Low | Low | Low | Moderate | Low | Low |
| Aalaei-Andabili et al. | Low | Low | Low | Low | Low | Low | Low | Low |
| Nagaraja et al. | Low | Moderate | Low | Low | Moderate | Low | Low | Low |
| Xiong et al. | Low | Low | Low | Low | Low | Low | Moderate | Low |
| Zhou et al. | Low | Moderate | Low | Low | Moderate | Moderate | Low | Moderate |
| Blackman et al. | Low | Low | Low | Low | Moderate | Low | Low | Low |
| Tchetche et al. | Low | Moderate | Low | Low | Moderate | Moderate | Low | Moderate |
| Elbadawi et al. | Low | Low | Low | Low | Moderate | Low | Low | Low |
| Forrest et al. | Low | Moderate | Low | Low | Moderate | Low | Low | Low |
| Waksman et al. | Low | Low | Low | Low | Moderate | Moderate | Low | Low |
| Fu et al. | Low | Low | Low | Low | Low | Low | Low | Low |
| Halim et al. | Low | Low | Low | Low | Low | Low | Moderate | Low |
| Pineda | Low | Low | Low | Low | Low | Low | Moderate | Low |
| Ahuja et al. | Low | Moderate | Low | Low | Low | Low | Low | Low |
| Du et al. | Low | Low | Low | Low | Moderate | Low | Low | Low |
| Hamdan et al. | Low | Low | Low | Low | Moderate | Moderate | Low | Low |
| Kim et al. | Low | Low | Low | Low | Moderate | Moderate | Low | Low |
| Williams et al. | Low | Low | Low | Low | Low | Moderate | Low | Low |
| Forrest et al. | Low | Low | Low | Low | Low | Moderate | Low | Low |
| Medranda et al. | Low | Low | Low | Low | Low | Moderate | Low | Low |
| Makkar et al. | Low | Moderate | Low | Low | Moderate | Low | Low | Low |

Supplementary Table 3. Studies included in the comparison of TAVR between **BE vs. SE devices in BAV patients**.

| ***Study*** | **Year** | **Design*** | **Center** | **Country** | | **Age ± SD or [IQR] (years)**** | | **Risk Score ± SD or [IQR] (%) *** | | **n. of patients **** | |
| --- | --- | --- | --- | --- | --- | --- | --- | --- | --- | --- | --- |
|  |  |  |  |  |  | **BE** | **SE** | **BE** | **SE** | **BE** | **SE** |
| Hayashida et al. | 2013 | OBS | 1 | France | | NA | NA | NA | NA | 11 - EG | 10 - EG |
| Costopoulos et al. | 2014 | OBS | 1 | Italy | | NA | NA | NA | NA | 8 - EG | 13 - EG |
| Mylotte et al. | 2014 | OBS | 12 | Europe and Canada | | 77.6 ± 9.7 | 78.2 ± 8.4 | STS-PROM 5.0 ± 3.9 | STS-PROM 4.8 ± 3.1 | 48 - EG | 91 - EG |
| Kosek et al. | 2015 | OBS | 1 | Poland | | 74.0 ± 1.0 | 79.2 ± 4.6 | EuroSCORE 19.0 ± 13.2 | EuroSCORE 20.2 ± 7.8 | 2 - EG | 5 - EG |
| Yousef et al. | 2015 | OBS | 21 | Canada, Spain, Italy, Poland and Singapore | | 74.4 ± 11.7 | 77.0 ± 8.0 | EuroSCORE 16.1 ± 13.8 | EuroSCORE 19.4 ± 11.9 | 61 - EG | 47 - EG |
| Watanabe et al. | 2015 | OBS | 1 | France | 82.5 ± 4.6 | | 84.4 ± 6.0 | EuroSCORE 21.3 11.6 | EuroSCORE 21.2 12.6 | 5 - EG | 6 - EG |
| Yoon et al. | 2016 | OBS | 20 | Europe, North America, and the Asia-Pacific | | 77.3 ± 9.6 | 76.6 ± 8.7 | STS-PROM 5.7 ± 6.1 | STS-PROM 3.6 ± 3.0 | 87 - EG 191 - EG | 112 - EG 11 - NG |
| Jilaihawi et al. | 2016 | OBS | 14 | Canada, China, France, Hong Kong, Italy, Germany, and USA | | 76.2 ± 11.6 | 77.0 ± 9.0 | STS-PROM 4.7 (2.8-7.4) | STS-PROM 4.7 (3.3-7.2) | 62 - EG 8 - NG | 60 - EG 0 - NG |
| Mangieri et al. | 2020 | OBS | 18 | Europe | | 77.4±8.6 | 78.6±7.5 | STS-PROM 4.4±3.3 | STS-PROM 4.2±3.3 | 242 - EG | 111 - EG |
| Husso et al. | 2020 | OBS | 5 | Finland | | NA | NA | NA | NA | 17 - EG 63 - NG | 2 - EG 21 - NG |

*BE represents balloon-expandable valve; SE represents self-expanding valve.

**EG represents prosthesis of Early Generation, NG represents prosthesis of New Generation.

Abbreviations: OBS = observational study, PSM = propensity score matching, STS-PROM, Society of Thoracic Surgeons Predicted Risk of Mortality; EuroSCORE, European System for Cardiac Operative Risk Evaluation.

.

Supplementary Table 4. Assessment of risk bias with ROBINS-I tool in the studies comparing **BE with SE in TAVI for BAV**

| **Study** | **Bias due to confounding** | **Bias in selection of participants into the study** | **Bias in classification of interventions** | **Bias due to deviations from intended interventions** | **Bias due to missing data** | **Bias in measurement of outcomes** | **Bias in selection of the reported result** | **Overall bias** |
| --- | --- | --- | --- | --- | --- | --- | --- | --- |
| Hayashida et al. | Low | Low | Low | Low | Low | Low | Moderate | Low |
| Costopoulos et al. | Low | Low | Low | Low | Low | Low | Moderate | Low |
| Mylotte et al. | Low | Moderate | Low | Low | Low | Low | Low | Low |
| Kosek et al. | Low | Low | Low | Low | Low | Moderate | Low | Low |
| Yousef et al. | Low | Low | Low | Low | Moderate | Low | Low | Low |
| Watanabe et al. | Low | Low | Low | Low | Low | Moderate | Low | Low |
| Yoon et al. | Low | Low | Low | Low | Moderate | Moderate | Low | Low |
| Jilaihawi et al. | Low | Low | Low | Low | Low | Low | Low | Low |
| Mangieri et al. | Low | Moderate | Low | Low | Low | Moderate | Low | Low |
| Husso et al. | Low | Moderate | Low | Low | Low | Low | Low | Low |

| **Study** | **Year** | **Design*** | **Center** | **Country** | **Age ± SD or [IQR] (years)** | | **Risk Score ± SD or [IQR] (%)** | | **n. of paitients*** | |
| --- | --- | --- | --- | --- | --- | --- | --- | --- | --- | --- |
|  |  |  |  |  | **BAV** | **TAV** | **BAV** | **TAV** | **BAV** | **TAV** |
| Watanabe et al. | 2015 | OBS | 1 | France | 82.5 ± 4.6 | 84.4 ± 6.0 | EuroSCORE 21.3 ± 11.6 | EuroSCORE 21.2 ± 12.6 | 11  Corevalve & Sapien | 56  Corevalve & Sapien |
| Kawamori et al. | 2018 | PSM | 1 | USA | 80 (70.5–83.0) | 83 (78.0–87.0) | - | - | 41  Sapien 3 | 236  Sapien 3 |
| Breirbart et al. | 2021 | PSM | 1 | Germany | 78.3 ± 5.7 | 81.8 ± 4.1 | STS-PROM 3.5 ± 2.6 | STS-PROM 4.1 ± 5.2 | 50  Sapien 3 | 50  Sapien 3 |
| Medranda et al. | 2021 | OBS | 6 | USA | 68.4 ± 7.8 | 76.0 ± 5.7 | STS-PROM 1.5 ± 0.6 | STS-PROM 1.9 ± 0.5 | 47  Sapien 3 | 60  Sapien 3 |

**Supplementary Table 5. Studies included in the comparison of TAVR between BAV vs TAV patients in post-procedural THV morphology.**

*EG represents prosthesis of Early Generation Valves , NG represents prosthesis of New Generation Valves.

Abbreviations: OBS = observational study, PSM = propensity score matching, STS-PROM, Society of Thoracic Surgeons Predicted Risk of Mortality; EuroSCORE, European System for Cardiac Operative Risk Evaluation.

.

Supplementary Table 6. Assessment of risk bias with ROBINS-I tool in the studies comparing THV morphology in TAVI for **BAV versus TAV** patients.

| **Study** | **Bias due to confounding** | **Bias in selection of participants into the study** | **Bias in classification of interventions** | **Bias due to deviations from intended interventions** | **Bias due to missing data** | **Bias in measurement of outcomes** | **Bias in selection of the reported result** | **Overall bias** |
| --- | --- | --- | --- | --- | --- | --- | --- | --- |
| **Watanabe et al.** | Low | Low | Low | Low | Low | Moderate | Low | Low |
| **Kawamori et al.** | Low | Low | Low | Low | Low | Moderate | Low | Low |
| **Breirbart et al.** | Low | Moderate | Low | Low | Low | Low | Low | Low |
| **Medranda et al.** | Low | Low | Low | Low | Low | Moderate | Low | Low |

**Supplementary Table 7. Rates of procedural complications and outcomes in BAV patients**

|  | Early generation devices | | New generation devices | |
| --- | --- | --- | --- | --- |
|  | Pooled rate (%) | 95% CI | Pooled rate (%) | 95% CI |
| Conversion to SAVR | 0.01 | 0-0.02 | 0 | 0-0.01 |
| Coronary obstruction | 0.02 | 0-0.03 | 0 | 0 |
| Need of a second valve | 0.06 | 0.02-0.10 | 0.02 | 0-0.04 |
| Moderate or severe PVL | 0.09 | 0.02-0.10 | 0 | 0-0.02 |
| Major vascular complication | 0.06 | 0.03-0.09 | 0.02 | 0-0.06 |
| Device failure | 0.11 | 0.05-0.20 | 0.07 | 0-0.18 |
| AKI | 0.07 | 0-0.20 | 0 | 0-0.02 |
| Life-threatening or major bleeding | 0.14 | 0.09-0.19 | 0.04 | 0.03-0.04 |
| MI | 0.01 | 0-0.03 | 0 | 0 |
| New PPI | 0.17 | 0.14-0.20 | 0.09 | 0.06-0.13 |
| Stroke | 0.01 | 0-0.03 | 0.01 | 0-0.01 |
| In-hospital mortality | 0.04 | 0.01-0.07 | 0 | 0 |
| 30-day stroke | 0.02 | 0.01-0.03 | 0.01 | 0-0.02 |
| 30-day new PPI | 0.19 | 0.12-0.25 | 0.11 | 0.07-0.16 |
| 30-day major vascular complication | 0.04 | 0.02-0.06 | 0.03 | 0-0.08 |
| 30-day MI | 0.03 | 0.01-0.07 | 0 | 0 |
| 30-day mortality | 0.05 | 0.04-0.07 | 0.01 | 0-0.01 |
| 1-year stroke | 0.03 | 0-0.07 | 0.01 | 0.01 |
| 1-year new PPI | 0.2 | 0.09-0.34 | 0.12 | 0.07-0.18 |
| 1-year mortality | 0.12 | 0.08-0.17 | 0.03 | 0.01-0.06 |

Abbreviation: SAVR, Surgical Aortic Valve Replacement; PVL, Paravalvular Leakage; AKI, Acute Kidney Injury; MI, Myocardial Infarction; PPI, Permanent Pacemaker Implantation.

**Supplementary Table 8. Rates of procedural complications and outcomes in TAV patients**

|  | Early generation devices | | New generation devices | |
| --- | --- | --- | --- | --- |
|  | Pooled rate | 95% CI | Pooled rate | 95% CI |
| Conversion to SAVR | 0.01 | 0-0.01 | 0 | 0 |
| Need of a second valve | 0.04 | 0.01-0.09 | 0 | 0-0.01 |
| Moderate or severe PVL | 0.06 | 0.01-0.12 | 0.01 | 0-0.02 |
| Major vascular complication | 0.05 | 0.02-0.08 | 0.02 | 0-0.06 |
| Device failure | 0.09 | 0.06-0.12 | 0.06 | 0-0.22 |
| AKI | 0.08 | 0-0.26 | 0 | 0-0.03 |
| Life-threatening or major bleeding | 0.33 | 0.29-0.38 | 0.03 | 0.01-0.05 |
| MI | 0.01 | 0-0.01 | 0 | 0 |
| New PPI | 0.21 | 0.13-0.32 | 0.06 | 0.03-0.11 |
| Stroke | 0.01 | 0-0.02 | 0 | 0-0.02 |
| In-hospital mortality | 0.24 | 0-0.79 | 0 | 0-0.01 |
| 30-day stroke | 0.02 | 0-0.03 | 0.01 | 0.01-0.02 |
| 30-day major vascular complication | 0.03 | 0.02-0.05 | 0.03 | 0-0.07 |
| 30-day mortality | 0.06 | 0.04-0.09 | 0.01 | 0-0.02 |
| 1-year stroke | 0.07 | 0.02-0.13 | 0.03 | 0.01-0.05 |
| 1-year new PPI | 0.21 | 0.05-0.43 | 0.17 | 0.04-0.36 |
| 1-year mortality | 0.12 | 0.08-0.16 | 0.05 | 0.03-0.09 |

Abbreviation: SAVR, Surgical Aortic Valve Replacement; PVL, Paravalvular Leakage; AKI, Acute Kidney Injury; MI, Myocardial Infarction; PPI, Permanent Pacemaker Implantation.

Supplementary Table 9. PRISMA 2020 Checklist.

| **Section and Topic** | **Item #** | **Checklist item** | **Location where item is reported** |
| --- | --- | --- | --- |
| **TITLE** | | |  |
| Title | 1 | Identify the report as a systematic review. | Page 1 |
| **ABSTRACT** | | |  |
| Abstract | 2 | See the PRISMA 2020 for Abstracts checklist. | Page 2 |
| **INTRODUCTION** | | |  |
| Rationale | 3 | Describe the rationale for the review in the context of existing knowledge. | Page 3 |
| Objectives | 4 | Provide an explicit statement of the objective(s) or question(s) the review addresses. | Page 3 |
| **METHODS** | | |  |
| Eligibility criteria | 5 | Specify the inclusion and exclusion criteria for the review and how studies were grouped for the syntheses. | Page 3 & 4 |
| Information sources | 6 | Specify all databases, registers, websites, organisations, reference lists and other sources searched or consulted to identify studies. Specify the date when each source was last searched or consulted. | Page 3 |
| Search strategy | 7 | Present the full search strategies for all databases, registers and websites, including any filters and limits used. | Page 3 |
| Selection process | 8 | Specify the methods used to decide whether a study met the inclusion criteria of the review, including how many reviewers screened each record and each report retrieved, whether they worked independently, and if applicable, details of automation tools used in the process. | Figure 1 |
| Data collection process | 9 | Specify the methods used to collect data from reports, including how many reviewers collected data from each report, whether they worked independently, any processes for obtaining or confirming data from study investigators, and if applicable, details of automation tools used in the process. | Page 4 & 5 |
| Data items | 10a | List and define all outcomes for which data were sought. Specify whether all results that were compatible with each outcome domain in each study were sought (e.g. for all measures, time points, analyses), and if not, the methods used to decide which results to collect. | Page 4 & 5 |
|  | 10b | List and define all other variables for which data were sought (e.g. participant and intervention characteristics, funding sources). Describe any assumptions made about any missing or unclear information. | Page 4 |
| Study risk of bias assessment | 11 | Specify the methods used to assess risk of bias in the included studies, including details of the tool(s) used, how many reviewers assessed each study and whether they worked independently, and if applicable, details of automation tools used in the process. | Page 5 |
| Effect measures | 12 | Specify for each outcome the effect measure(s) (e.g. risk ratio, mean difference) used in the synthesis or presentation of results. | Page 5 |
| Synthesis methods | 13a | Describe the processes used to decide which studies were eligible for each synthesis (e.g. tabulating the study intervention characteristics and comparing against the planned groups for each synthesis (item #5)). | Page 4 |
|  | 13b | Describe any methods required to prepare the data for presentation or synthesis, such as handling of missing summary statistics, or data conversions. | Page 5 |
|  | 13c | Describe any methods used to tabulate or visually display results of individual studies and syntheses. | Page 5 |
|  | 13d | Describe any methods used to synthesize results and provide a rationale for the choice(s). If meta-analysis was performed, describe the model(s), method(s) to identify the presence and extent of statistical heterogeneity, and software package(s) used. | Page 5 |
|  | 13e | Describe any methods used to explore possible causes of heterogeneity among study results (e.g. subgroup analysis, meta-regression). | No information |
|  | 13f | Describe any sensitivity analyses conducted to assess robustness of the synthesized results. | No information |
| Reporting bias assessment | 14 | Describe any methods used to assess risk of bias due to missing results in a synthesis (arising from reporting biases). | Page 5 |
| Certainty assessment | 15 | Describe any methods used to assess certainty (or confidence) in the body of evidence for an outcome. | Page 5 |
| **RESULTS** | | |  |
| Study selection | 16a | Describe the results of the search and selection process, from the number of records identified in the search to the number of studies included in the review, ideally using a flow diagram. | Page 5-7 (Figure 1) |
|  | 16b | Cite studies that might appear to meet the inclusion criteria, but which were excluded, and explain why they were excluded. | No information |
| Study characteristics | 17 | Cite each included study and present its characteristics. | Page 5 and Supplementary Table 1, 3 and 5 |
| Risk of bias in studies | 18 | Present assessments of risk of bias for each included study. | Page 5-7 and Supplementary Table 2, 4 and 6 |
| Results of individual studies | 19 | For all outcomes, present, for each study: (a) summary statistics for each group (where appropriate) and (b) an effect estimate and its precision (e.g. confidence/credible interval), ideally using structured tables or plots. | Page 5-7 and Supplementary Figure 1 and 2 |
| Results of syntheses | 20a | For each synthesis, briefly summarise the characteristics and risk of bias among contributing studies. | Page 7 and 8 |
|  | 20b | Present results of all statistical syntheses conducted. If meta-analysis was done, present for each the summary estimate and its precision (e.g. confidence/credible interval) and measures of statistical heterogeneity. If comparing groups, describe the direction of the effect. | Page 5-7 and Supplementary Figure 1 and 2 |
|  | 20c | Present results of all investigations of possible causes of heterogeneity among study results. | Page 5-7 |
|  | 20d | Present results of all sensitivity analyses conducted to assess the robustness of the synthesized results. | No information |
| Reporting biases | 21 | Present assessments of risk of bias due to missing results (arising from reporting biases) for each synthesis assessed. | Page 5-7 and Supplementary Table 2, 4 and 6 |
| Certainty of evidence | 22 | Present assessments of certainty (or confidence) in the body of evidence for each outcome assessed. | Page 5-7 and Supplementary Figure 1 and 2 |
| **DISCUSSION** | | |  |
| Discussion | 23a | Provide a general interpretation of the results in the context of other evidence. | Page 7 |
|  | 23b | Discuss any limitations of the evidence included in the review. | Page 10 |
|  | 23c | Discuss any limitations of the review processes used. | Page 10 |
|  | 23d | Discuss implications of the results for practice, policy, and future research. | Page 9-10 |
| **OTHER INFORMATION** | | |  |
| Registration and protocol | 24a | Provide registration information for the review, including register name and registration number, or state that the review was not registered. | Page 1 |
|  | 24b | Indicate where the review protocol can be accessed, or state that a protocol was not prepared. | Page 1 |
|  | 24c | Describe and explain any amendments to information provided at registration or in the protocol. | Page 1 |
| Support | 25 | Describe sources of financial or non-financial support for the review, and the role of the funders or sponsors in the review. | Page 1 |
| Competing interests | 26 | Declare any competing interests of review authors. | Page 1 |
| Availability of data, code and other materials | 27 | Report which of the following are publicly available and where they can be found: template data collection forms; data extracted from included studies; data used for all analyses; analytic code; any other materials used in the review. | Page 4 |

*From:*  Page MJ, McKenzie JE, Bossuyt PM, Boutron I, Hoffmann TC, Mulrow CD, et al. The PRISMA 2020 statement: an updated guideline for reporting systematic reviews. BMJ 2021;372:n71. doi: 10.1136/bmj.n71
